# Synthesis, *in vitro* Antileishmanial Efficacy and Hit/Lead Identification of Nitrofurantoin‐Triazole Hybrids

**DOI:** 10.1002/cmdc.202200023

**Published:** 2022-05-02

**Authors:** Nonkululeko H. Zuma, Janine Aucamp, Maryna Viljoen, David D. N'Da

**Affiliations:** ^1^ Centre of Excellence for Pharmaceutical Sciences (Pharmacen) Faculty of Health Sciences North-West University 11 Hoffmann Street Potchefstroom 2520 South Africa; ^2^ School of Pharmacy, Faculty of Health Sciences North-West University 11 Hoffmann Street Potchefstroom 2520 South Africa

**Keywords:** amastigote, hybrid, leishmaniasis, nitrofurantoin, triazoles

## Abstract

Leishmaniasis is a vector‐borne neglected parasitic infection affecting thousands of individuals, mostly among populations in low‐ to moderate‐income developing countries. In the absence of protective vaccines, the management of the disease banks solely on chemotherapy. However, the clinical usefulness of current antileishmanial drugs is threatened by their toxicity and the emergence of multidrug‐resistant strains of the causative pathogens. This emphasizes the imperative for the development of new and effective antileishmanial agents. In this regard, we synthesized and evaluated *in vitro* the antileishmanial activity and cytotoxicity profile of a series of nitrofurantoin‐triazole hybrids. The nitrofurantoin derivative **1** featuring propargyl moiety was distinctively the most active of all, was nontoxic to human cells and possessed submicromolar cellular activity selectively directed towards the pathogens of the life threatening visceral leishmaniasis. Hence it was identified as potential antileishmanial lead for further investigation into its prospective to act as alternative to therapies.

## Graphical abstract

**In the absence** of protective vaccines, the management of leishmaniasis depends solely on chemotherapy. We synthesized nitrofurantoin‐triazole hybrids and screened them for antileishmanial activity. Compound **1** was identified as an early lead.

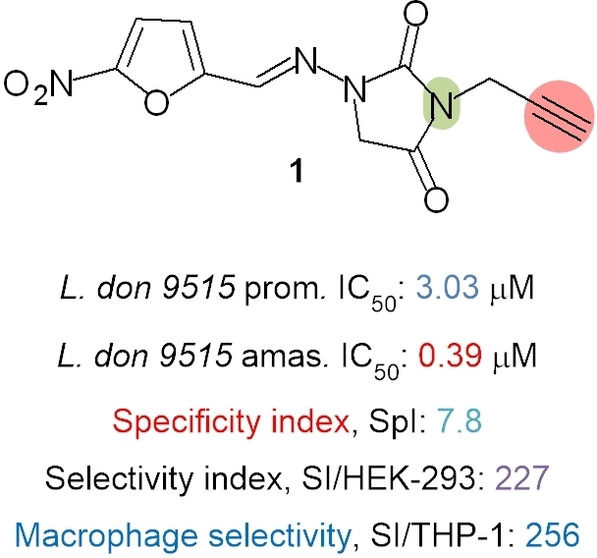

## Introduction

Leishmaniasis is a neglected tropical disease (NTD) that presents a major health challenge in many developing countries. It is endemic to tropical and sub‐tropical countries in Asia, Africa, the Americas, and the Mediterranean region.^[1]^ Over twenty *Leishmania* species communicate three clinical forms of the disease: cutaneous leishmaniasis (CL), mucocutaneous leishmaniasis (MCL) and visceral leishmaniasis (VL). The world Health Organization (WHO) estimates the global occurrence of 50 000–90 000 new cases of VL and 700 000–1 million new cases of CL annually.^[1]^

Due to challenges in vector control and the lack of protective vaccines, the management of leishmaniasis relies solely on chemotherapy. Only a handful of clinical drugs, including pentavalent antimonials, pentamidine, miltefosine, paromomycin, and liposomal amphotericin B, are available for use either as monotherapy or in combination to treat this infection.^[2]^ However, these drugs are toxic, and their overuse has resulted in the emergence of multidrug‐resistant strains of the causative pathogen, hence the imperative to develop new, effective, and cost‐efficient antileishmanial agents.^[3]^

Traditional drug development, referred to as *de novo* drug discovery and development, is an expensive and laborious task. The process from laboratory design to clinical drug can take an estimated thirteen years.^[4]^ An alternative to this costly and lengthy process is drug repositioning or repurposing, whereby new applications for existing or abandoned drugs are discovered and refined.^[4]^ The drug may be repurposed as is or modified by either molecular derivatization or hybridization. The former involves exploitation of the structure‐activity relationship (SAR) of clinical drugs to deliver analogues with improved therapeutic profiles.^[5]^ The latter is the assemblage of two or more different drugs or pharmacologically active moieties into a single chemical entity by chemical reactions.^[6]^ The advantage of drug repurposing is that the properties, such as pharmacokinetic and safety profiles, of the drug of interest are already known, consequently shortening the drug development process.^[7]^

**Figure 1:**
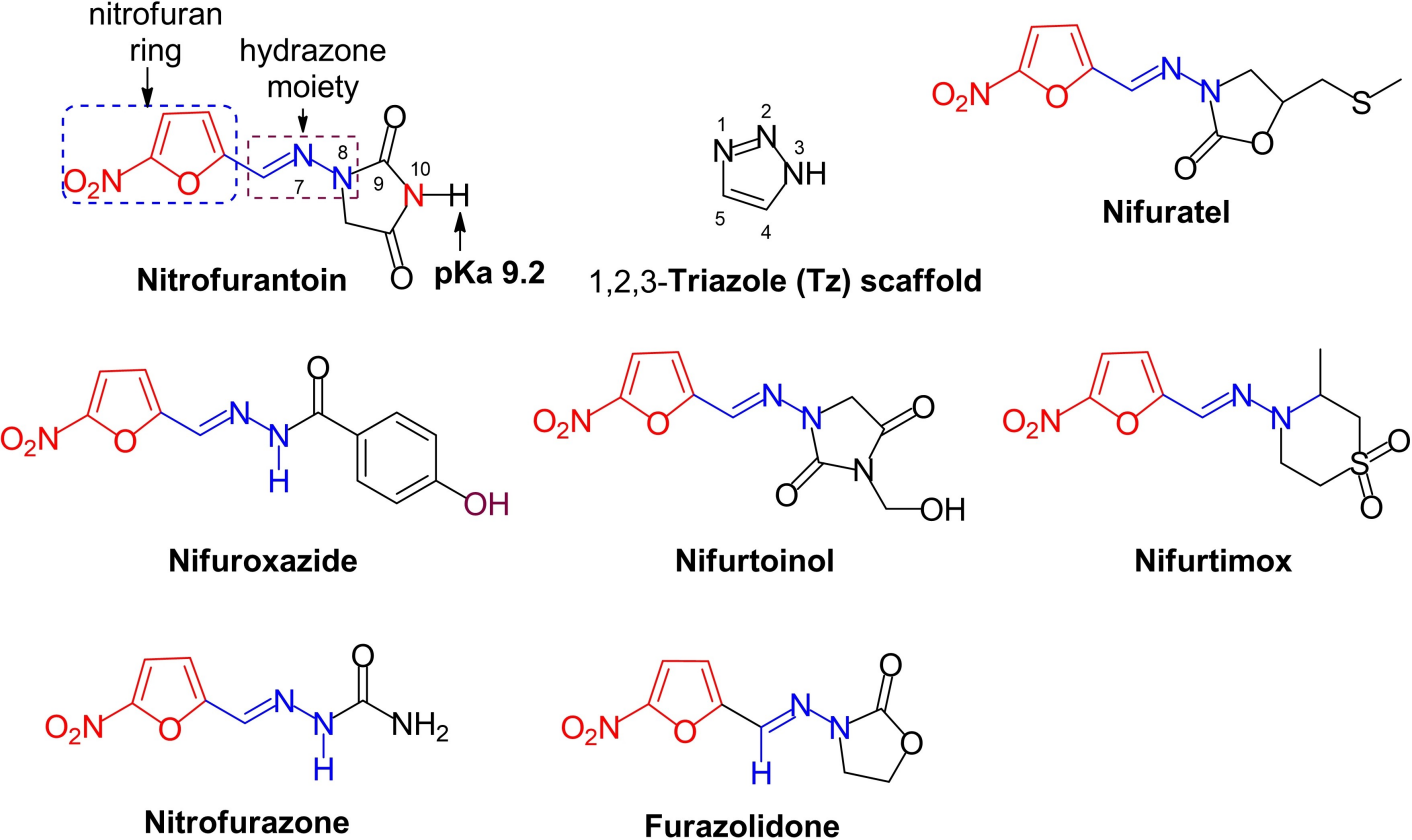
Anti‐infective clinical nitrofuran drugs and 1,2,3‐triazole core.

Nitrofuran drugs (NFs) (Figure 1) play a significant role in the management of kinetoplastid (flagellated protozoan parasite) infections. Indeed, nifurtimox has been an antichagasic drug over five decades^[8]^ and is used, in combination with eflornithine (NECT), for the treatment of late‐stage human African trypanosomiasis (HAT) infection caused by *Trypanosoma brucei* (*T. b*.) *gambiense*.^[9]^ Nifuroxazide and nitrofurazone demonstrate potent antileishmanial activity.^[10]^ Furthermore, the taxonomic similarities among kinetoplastid parasites of the of the *Trypanosomatidae* family (e. g., *Leishmania* and *Trypanosoma*)^[11]^ may further promote potential dual activity, i. e., antitrypanosomal drugs acting as potential antileishmanial therapy and vice versa.

Nitrofurantoin (NFT, Figure 1) is a cheap clinical antibiotic belonging to the class of NF drugs, that has been found with antimicrobial activities against aerobic and anaerobic infective pathogens,^[12]^ and has been used in the treatment of several animal and human infections. As a NF derivative, NFT exerts its biological activities through two environment‐specific mechanism of actions involving the primary pharmacophore, i. e., nitro group.^[13]^ These include nitroreduction type I (NTR−I) that occurs under anaerobic conditions, and nitroreduction type II (NTR‐II) that takes place in an aerobic environment. Both mechanisms are catalyzed by pathogen‐specific nitroreductases^[14]^ and result in the generation of toxic molecular species, e. g., nitroso, hydronitroso and hydroxylamine in NTR−I,^[15]^ and reactive oxygen species (ROS), e. g., superoxide ion radical (⋅O_2_) and hydroxyl radical (⋅OH)^[16]^ in NTR‐II, that cause parasite death by oxidative stress. This multi‐targeted activity promotes the prevention of pathogen resistance, a common issue experienced by many therapeutic drugs used in the treatment of infectious diseases.^[17]^ However, the pharmacological usefulness of NFT is limited by its poor water solubility and oral bioavailability,^[18]^ with 33 % being excreted unchanged during therapy.^[19]^

On the other hand, triazoles are the building blocks for different active anti‐infective compounds. In particular, 1,2,3‐Triazole (Tz core, Figure 1), a five‐member *N*‐heterocyclic scaffold, has been found to endow various biological properties when incorporated into synthetic compounds, including antileishmanial^[20]^ and trypanosomal.^[21]^ These properties are imparted as result of Tz possessing favourable physicochemical features such as hydrogen bonding capability, moderate dipole, rigidity^[22]^ and high water solubility.^[23]^ Some of these triazolyl compounds exert their biological action by inhibiting microbial cell wall synthesis through blocking of lipid biosynthesis,[^22^, ^24^] hence the often use of Tz in molecular hybridization drug design.^[25]^

Altogether, these facts motivated the current investigation of nitrofurantoin‐1,2,3‐triazole hybrids with the intentions to improve the physicochemical (e. g., water solubility) and pharmacokinetic (e. g., oral bioavailability) shortcomings and ultimately enhance the clinical stand of NFT as a viable alternative to the existing antileishmanial drugs. We herein portray the synthesis, the biological activities and safety profile of these triazolyl NFT derivatives.

## Results and Discussion

### Chemistry

NFT is poorly soluble in most organic solvents as well as in water (0.19 mg/mL) with the latter improving under basic conditions.^[26]^ DMSO (25 mg/mL) and DMF (50 mg/mL) are the only solvents in which the drug has moderate solubility. However, in this study the synthesis did not proceed in DMSO, hence DMF was the solvent choice. No further special conditions and precautions, viz., inert conditions or high/low temperature, were necessary for the synthesis. The molecular hybridization strategy, owing to the high hydrophilicity of the 1,2,3‐triazole moiety,^[23]^ resulted in hybrids with better solubility in organic solvents such as ethyl acetate and ethanol. The hybrids required extra effort to purify, hence they were recrystallized at least twice in ethanol/water (1 : 1, v/v) mixture.

Furthermore, to exert pharmacological activity, a drug must permeate through several biological membranes to reach its site action. Permeability is governed by a multifunctional interaction of factors including molecular size, lipophilicity, polar van der Waals surface, molecular flexibility and protein binding.^[27]^ Thus, hybrids containing a single carbon, i. e., methylene linker, were targeted to ensure minimal flexibility between the pharmacophores. Additionally, to diversify the chemical structures of the hybrids, *n*‐alkyl (*n*=5–12) or benzyl groups (R) were substituted on the 1,2,3‐triazole ring.

To establish if electronic effect could influence the biological activity, electron donating groups (EDGs), (CH_3_, *i*Pr and *t*‐Bu) and electron withdrawing groups (EWGs) (F, Br, CF_3_ and NO_2_), were added to further diversify the substituents on the benzyl group moiety.

**Scheme 1:**
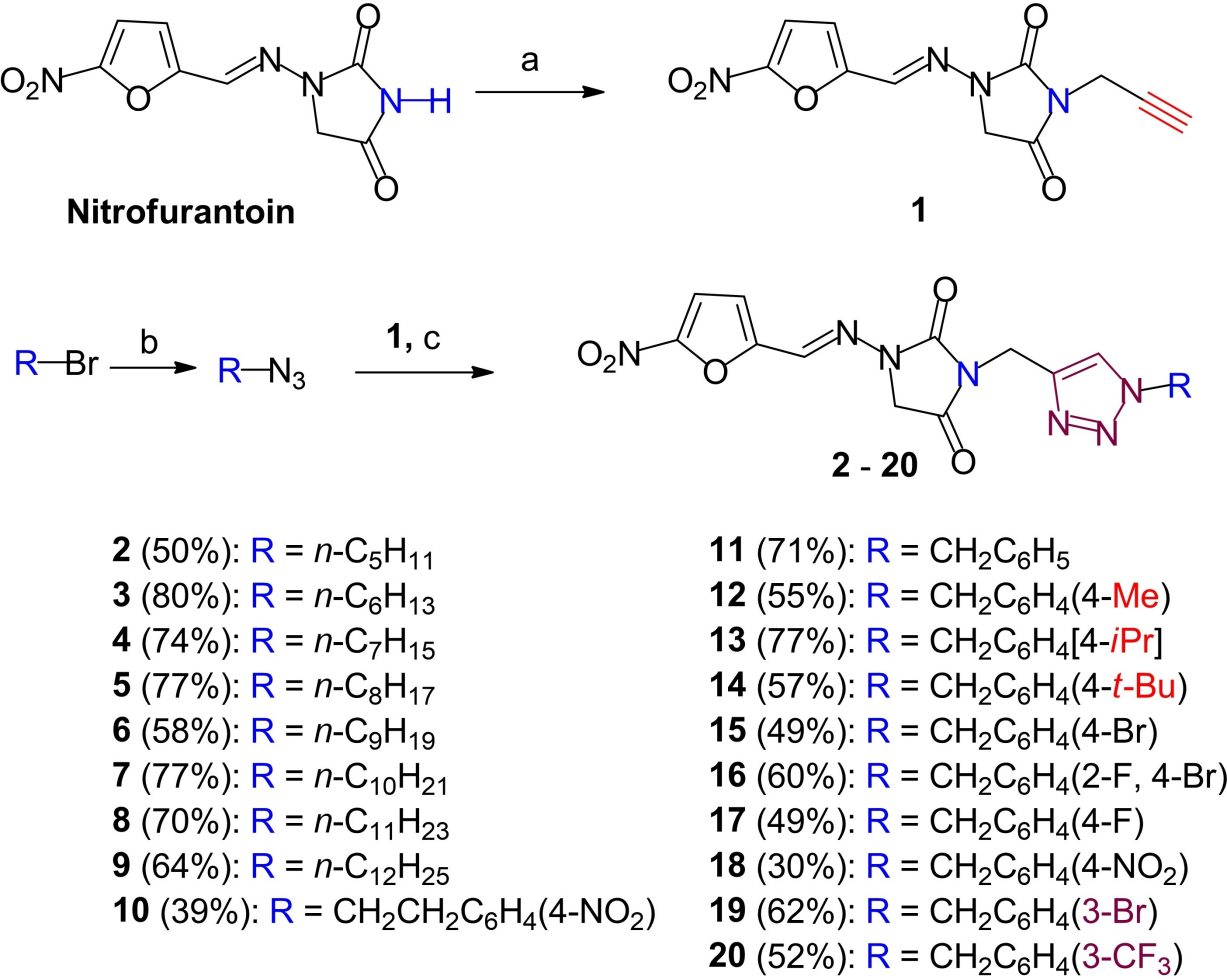
Three‐step synthesis of nitrofurantoin‐triazole hybrids. *Reagents and conditions*: a. propargyl bromide (1.5 equiv.), TEA (1.5 equiv), anhydrous DMF, rt, 24 h; b. alkyl/benzyl bromide, sodium azide (1.5 equiv), DMSO, rt, overnight;^[28]^ c. alkyl/benzyl azide (1.5 equiv), *β*‐cyclodextrin (0.02 equiv), sodium ascorbate (0.3 equiv) and copper sulphate pentahydrate (0.1 equiv), DMSO:H_2_O (4 : 1, v/v), rt, 48 h.

The hybrids were successfully synthesized through a three‐step process involving two nucleophilic substitutions (S_N_2) and a copper‐catalyzed alkyne‐azide cycloaddition (CuCAAC) reaction (Scheme 1). First, nitrogen atom N‐10 bearing the weakly acidic proton H‐10 (pKa 9.2 as calculated on MarvinSketch Version 19.4, ChemAxon Software, www.ChemAxon.com, Figure 1) was easily deprotonated with a mild base, triethylamine (TEA) (pKa 10.2), and was reacted with propargyl bromide to afford the propargylated NFT intermediate **1** (Scheme 1a). Second, in parallel, alkyl/benzyl bromides were treated with sodium azide in another S_N_2 reaction^[28]^ to provide alkyl/benzyl azide intermediates (Scheme 1b). Third, CuAAC reaction, or otherwise referred to as click‐chemistry (Scheme 1c), involving both intermediates, afforded the target hybrids^[29]^ in moderate (50 %) to good (80 %) yields except for hybrid **19**, which had a poor yield (30 %). In this last reaction, β‐cyclodextrin was used as phase transfer catalyst (PTC), facilitating the transport of lipophilic reagents, e. g., *n*‐alkyl/aryl azide and alkynes, into the aqueous phase^[29]^ where the reaction took place.

The structures of all hybrids were confirmed by ^1^H and ^13^C nuclear magnetic resonance (NMR) as well as high resolution mass spectrometry (HRMS) and infrared (IR). HPLC purity determination indicated good purity margins (>90 %) for majority of the compounds. The molecular hybridization resulted in hybrids with better solubility in organic solvents, such as ethyl acetate and ethanol.

The molecular integrity of the NFT scaffold was confirmed by markers, such as: (i) resonance of hydantoin ring H‐12 proton which was consistently assigned to the singlet *ca*. δ 4.50 ppm; (ii) the singlet associated with the hydrazine vinyl H‐6 proton appearing in the δ 8.65–7.99 ppm region, and (iii) the preservation of the furan ring confirmed by the two distinctive doublets associated with the resonance of the aromatic protons H‐3 and H‐4 in the δ 7.17–7.03 ppm and δ 7.91–7.70 ppm regions, respectively.

Successful hybridization was confirmed by the disappearance of the singlet associated with H‐10 ca. δ 11.50 ppm from the ^1^H spectra of compounds **1**–**20**, in addition to the appearance of singlets assigned the methylene (CH_2_) of protons H‐1’ and H‐1’’ (benzyl) in the δ 3.5–4.5 and 5.60–5.52 ppm regions, respectively. Furthermore, the singlet that appeared ca. δ 8.3–7.9 ppm was attributed to the resonance of the triazolyl proton H‐3’. All protons of each structure were accounted for.

The IR spectra confirmed the presence of the carbonyl groups (C=O) C‐9 and C‐11 of the hydantoin ring, which occurred in the regions of 1790–1768 and 1730–1703 cm^−1^, respectively, and the presence of the Tz (C−N) as indicated by the peak in 1340–1388 cm^−1^. Similarly, HRMS attested of the presence of the molecular ion fragment of each hybrid.

### In Silico Molecular and ADME Properties

Good solubility improves drug permeability through biological membranes, which in turn enhances drug absorption and consequently drug bioavailability.^[30]^ Therefore, the lipophilicity and hydrophilicity balance (by inference log*P*) are crucial for a drug's ultimate efficacy. High lipophilicity is generally associated with drug toxicity, whereas high hydrophilicity indicates poor permeability and hence low absorption.^[31]^ The Lipinski's rule of five for drug‐like properties indicates that log*P*, which is defined as the octanol/water partition coefficient, should be <5.^[32]^ The log*P* offers a reliable measure of the balance between lipophilicity and aqueous solubility. Although not a true reflection of experimental values, clog*P* provides a good estimate of the balance between lipophilicity and hydrophilicity. For validated hit and lead compounds intended for the treatment of infectious diseases, the criteria are log*P*<5, and ideally <3.^[33]^

Furthermore, to date, miltefosine is the only oral antileishmanial in clinical use. However, the very use of it is jeopardized by pathogenic resistance, hence the need for alternative oral drugs. Accordingly, the molecular (e. g., lipophilicity and aqueous solubility) and pharmacokinetic (ADME: absorption, metabolism, distribution, and excretion) properties were predicted for all the synthesized hybrids and clinical NF drugs using SwissADME web tool. Lipinski's rule of five (RO5)^[32]^ was then used to predict the drug‐likeness and oral activity likeness of the compounds in humans. The data are reported in Table 1.

**Table 1:** Molecular and ADME properties of synthesized nitrofurantoin‐triazole hybrids and clinical nitrofuran drugs as predicted by SwissADME web tool, http://www.swissadme.ch.

Cpd.	MW^[a]^ [g/mol]	Log*P*_o/w_^[b]^	Log *S* ^[c]^		RB^[f]^	HBD^[g]^	HBA^[h]^	PSA [Å^2^]^[i]^	Lipinski's violation^[j]^	GI absorption^[k]^	Drug‐likeness^[l]^
			ESOL^[d]^	Ali^[e]^							
NFT	238.16	‐0.50	−1.04	−1.60	3	1	6	120.73	0	High	Yes
1	276.21	0.22	−1.37	−1.37	4	0	6	111.94	0	High	Yes
2	389.37	0.72	−2.89	−4.14	9	0	8	142.65	1	Low	Yes
3	403.39	1.19	−3.25	−4.71	10	0	8	142.65	1	Low	Yes
4	417.42	1.54	−3.60	−5.27	11	0	8	142.65	1	Low	Yes
5	431.45	1.84	−3.95	−5.83	12	0	8	142.65	1	Low	Yes
6	445.47	2.17	−4.31	−6.39	13	0	8	142.65	1	Low	Yes
7	459.50	2.55	−4.66	−6.95	14	0	8	142.65	1	Low	Yes
8	473.53	2.94	−5.02	−7.52	15	0	8	142.65	1	Low	Yes
9	487.55	3.33	−5.38	−8.08	16	0	8	142.65	1	Low	Yes
10	468.38	0.42	−3.46	−5.10	9	0	10	188.47	1	Low	Yes
11	409.36	0.89	−3.20	−4.01	7	0	8	142.65	1	Low	Yes
12	423.38	1.03	−3.50	−4.38	7	0	8	142.65	1	Low	Yes
13	451.44	1.60	−4.06	−5.17	8	0	8	142.65	1	Low	Yes
14	465.46	1.88	−4.49	−5.74	8	0	8	142.65	1	Low	Yes
15	488.25	1.55	−4.11	−4.73	7	0	8	142.65	1	Low	Yes
16	506.24	1.84	−4.27	−4.83	7	0	9	142.65	2	Low	No
17	427.35	1.19	−3.36	−4.11	7	0	9	142.65	1	Low	Yes
18	454.35	0.20	−3.26	−4.79	8	0	10	188.47	1	Low	Yes
19	466.25	0.89	−3.47	−4.21	8	0	8	142.65	1	Low	Yes
20	477.35	1.98	−4.06	−4.92	9	0	11	142.65	1	Low	Yes
FZD	225.16	0.15	−1.24	−1.62	3	0	6	100.86	0	High	Yes
NFZ	198.14	‐0.59	−1.21	−2.45	4	2	5	126.44	0	High	Yes
NFX	275.22	0.90	−2.95	−4.27	5	2	6	120.65	0	High	Yes

[a] Molecular weight. [b] Calculated logP (consensus log P). [c] Predicted aqueous solubility, where log S is the logarithm of the amount of compound (in moles) able to dissolve a liter of water. [d] ESOL=estimated aqueous solubility, calculated using a topological method.^[34]^ [e] Calculated using a topological method^[35]^ with log S scale: insoluble<−10<poorly<−6<moderately<−4<soluble<−2 very soluble <0 highly <. [f] Number of rotatable bonds (RB). [g] Number of hydrogen bond donors (HBD). [h] Number of hydrogen bond acceptors (HBA). [i] Polar surface area (PSA), RB≤10 and PSA≤140 Å^2^ – good oral bioavailability.^[36]^ [j] Determined with reference to Lipinski's rule of five: MW≤500 g/mol; Log*P*≤5; RB≤10; HBD≤5 (NH or OH); HBA≤10 (N or/and O), no more than one violation allowed.^[32]^ [k] According to the white of the BOILED‐Egg.^[37]^ [l] According to Lipinski et al.^[32]^ All values in this table were calculated using SwissADME web tool, http://www.swissadme.ch.^[38]^ NFT: nitrofurantoin; NFA: 5‐nitro‐2‐furaldehyde; FZD: furazolidone; NFZ: nitrofurazone; NFX: nifuroxaxide.

From the results, it can be observed that apart from **16**, all hybrids complied with Lipinski's rules albeit, one violation (N or O>10), and therefore were predicted to be druglike, having the physicochemical properties well within the target ranges.^[32]^ However, their oral bioavailability was predicted to be poor (PSA≥140 Å^2^)^[36]^ due to low GI absorption through passive diffusion, which may be suggestive of their unsuitability for oral administration. On the other hand, the propargyl NFT intermediate **1** and the clinical NFs (NFT, furazolidone, nitrofurazone and nifuroxazide) drugs were confirmed to be druglike and suitable for oral therapies (RB<10 and PSA<140 Å^2^).^[36]^

### Pharmacology

Numerous challenges must be overcome to develop new anti‐infective drugs. This has led experts in the field of drug discovery to establish various strategies to fast‐track this lengthy process. For instance, at early discovery, i. e., basic research stage, Katsuno et al. (2015)^[33b]^ set out a series of criteria to identify hit and lead compounds intended for the treat infectious diseases (e. g., malaria, TB, leishmaniasis and trypanosomiasis) occurring the developing countries. Regarding leishmaniasis, among others, a hit should demonstrate cellular potency with IC_50_/ED_50_<10 μM against intracellular *L. donovani* amastigotes and a 10‐fold selectivity for mammalian (e. g., Vero or HepG2 etc.) cells. A lead, on the other hand, should possess cellular potency with IC_50_/ED_50_<1 μM against *L. donovani* amastigotes with 100‐fold selectivity for mammalian cells. With these criteria in mind, literature‐specific assays (Siqueira‐Neto et al., 2010; De Mulder et al., 2011) were adopted to identify potential antileishmanial hits/lead in this study. The process occurred in two steps. The synthesized compounds were first screened at 10 μM single concentration for parasite growth inhibition using Resazurin assay. Second, compounds exhibiting 70 % and/or 60 % inhibition of promastigote and amastigote, respectively, were selected for concentration‐response study for the determination of antileishmanial IC_50_ values.

The hybrids and clinical NF antibiotics were thus assessed against two *Leishmania* strains, *L. donovani* 9515, an antimonial‐resistant^[39]^ and *L. major* IR‐173. These species were selected to determine the specificity of the compounds against *L. donovani* parasites that instill the life threatening VL^[40]^ and *L. major* that communicate the stigmatizing CL to mammals. The clinical antileishmanial drug amphotericin B (AMB) served as reference drug.

*Leishmania* species have two developmental forms, promastigote (in insect vector) and amastigote (in mammal hosts). The latter instills clinical symptoms of the infection and progression to the disease hence has been the logical targeted form during the process of antileishmanial drug development.^[33b]^ Moreover, studies have shown that antileishmanial drugs that are effective against amastigotes are not always effective against promastigotes^[41]^ while only 4 % of identified antipromastigote hits are confirmed as antiamastigote hits in screening.^[40]^ Therefore, there may be a significant benefit in screening compounds against both parasite forms.

The antileishmanial activity was assessed by determining the half‐maximum inhibitory concentration (IC_50_) value of the compounds against each parasite form, and the specificity index (SpI) which gives account of the preferential form targeted by each compound, was deduced. The basal toxicity profile was determined on human embryonic kidney (HEK‐293) cells and the related selectivity index (SI) value for each compound was deduced. Host cell toxicity was also investigated using macrophages from human acute monocytic leukemia (THP‐1) cell line and the related selectivity indexes were unraveled. The well‐known toxic drug, emetine served as negative control in cytotoxicity assay. All biological results are reported in Tables 2 and 3.

**Table 2:** *In vitro* antileishmanial activities and cytotoxicity data of synthesized nitrofurantoin‐triazole hybrids and nitrofuran reference drugs.

Compd	Cytotoxicity, HEK‐293, IC_50_±SD [μM]	Antipromastigote activity, IC_50_ [μM]±SD		Anti‐amastigote activity, IC_50_ [μM]±SD		Specificity index^[a]^		Selectivity index	
		IR‐173	9515	IR‐173	9515	SpI_1_^[b]^	SpI_2_^[c]^	SI_1_^[d]^	SI_2_^[e]^
NFT	>100	>10	>10	>10	9.92±0.11	–	–	3	10
1	88.49±1.38	0.51±0.05	3. 03±0.06	–	0.39±0.12	–	7.8	–	227
2	>100	0.09±0.00	–	3.95±0.41	–	0.02	–	25	–
3	17.87±1.30	0.48±0.02	–	–	–	–	–	–	–
4	>100	6.85±0.44	0.18±0.05	–	0.38±0.06	–	0.5	–	>263
5	31.87±3.55	0.27±0.02	–	–	–	–	–	–	–
6	43.84±4.13	6.51±1.13	4.68±0.32	–	3.69±0.16	–	1.3	–	12
7	67.13±5.22	0.34±0.02	–	6.72±0.00	6.17±0.47	0.05	–	10	11
8	20.34±3.15	1.24±0.13	1.60±0.08	–	4.49±0.00	–	0.4	–	5
9	1.24±0.10	0.38±0.01	–	–	–	–	–	–	–
10	43.70±3.37	0.17±0.00	–	–	–	–	–	–	–
11	20.97±3.25	0.68±0.13	–	–	–	–	–	–	–
12	41.79±1.21	0.34±0.11	–	–	–	–	–	–	–
13	7.27±0.93	0.57±0.01	–	–	9.13±0.00	–	–	–	1
14	23.22±2.77	0.45±0.10	–	–	1.16±0.02	–	–	–	20
15	32.66±4.19	0.50±0.03	7.59±0.56	5.14±0.67	–	0.1	–	6	–
16	14.38±0.84	0.19±0.01	–	6.42±0.38	9.21±0.00	0.5	–	34	2
17	48.43±1.88	1.05±0.07	–	–	–	–	–	–	–
18	60.20±2.29	0.38±0.00	–	–	–	–	–	–	–
19	12.56±0.46	0.48±0.00	1.75±0.11	–	–	–		–	–
20	15.76±3.69	0.13±0.02	–	–	–	–	–	–	–
FZD	>100	0.37±0.02	0.57±0.05	2.80±0.50	4.11±0.78	0.1	0.1	36	24
NFZ	>100	1.22±0.03	1.82±0.03	5.75±0.75	4.94±0.61	0.2	0.4	17	20
NFX	>100	>10	4.48±0.10	>10	7.29±0.39	–	0.6	–	14
AMB	57.77±3.22	0.03±0.01	0.02±0.00	0.03±0.00	0.05±0.00	1	0.4	1926	1155
EM	0.01±0.00	–	–	–	–	–	–	–	–

**Table 3:** Macrophage selectivity.

Compd	Cytotoxicity, THP‐1, IC_50_±SD (μM)	Selectivity index	
		SI_3_^[f]^	SI_4_^[g]^
NFT	>100	3	10	
1	99.94±0.09	–	256	
2	>100	25	–	
3	–	–	–	
4	>100	–	>263	
5	–	–	–	
6	>100	–	27	
7	>100	15	16	
8	>100	–	22	
9	>100	–	–	
10	>100	–	–	
11	–	–	–	
12	–	–	–	
13	>100	–	11	
14	>100	–	86	
15	>100	19	–	
16	55.73±1.17	9	6	
17	–	–	–	
18	–	–	–	
19	–	–	–	
20	–	–	–	
FZD	>100	36	24	
NFZ	>100	17	20	
NFX	>100	7	14	
AMB	14.86±0.09	495	297	

[a] Specificity index (SpI) <0.4 indicates more antipromastigote activity, 0.4<SpI<2.0 indicates activity against both forms, SpI>2.0 indicates more anti‐amastigote activity.^[41b]^ [b] Specificity index of *L. major* IR‐173: SpI_1_=IC_50_ promastigote/IC_50_ amastigote. [c] Specificity index of *L. donovani* 9515: SpI_2_=IC_50_ promastigote/IC_50_ amastigote. [d] Selectivity Index of *L. major* IR‐173: SI_1_=IC_50_ HEK‐293/IC_50_ amastigote. [e] Selectivity Index of *L. donovani* 9515: SI_2_=IC_50_ HEK‐293/IC_50_ amastigote. [f] Selectivity Index of *L. major* IR‐173: SI_3_=IC_50_ THP‐1/IC_50_ amastigote. [g] Selectivity Index of *L. donovani* 9515: SI_4_=IC_50_ THP‐1/IC_50_ amastigote. HEK‐293: human embryonal kidney cells; THP‐1: Human acute monocytic leukemia; NFT: nitrofurantoin; FZD: furazolidone; NFX: nifuroxazide; NFZ: nitrofurazone; AMB: amphotericin B; EM: Emetine. All reported data were significant at *p*<0.05.

Toxicity accounts for more than a third (>33 %) of drug failure in clinical settings, as well as a significant amount of drug development (clinical trials and post‐marketing) costs.^[42]^ Therefore, the screening for toxicity early in the drug development process is invaluable. Starting from NFT, a low cytotoxicity clinical drug (IC_50_>100 μM),^[43]^ allowed the establishment of an initial benchmark for *in vitro* toxicity comparison. The standard AMB, intermediate **1** and most hybrids possessed mild‐to‐weak basal toxicity to HEK‐293 cells (10<IC_50_<100 μM) apart from **9** and **13** (IC_50_<10 μM), which were significantly toxic.^[44]^ Hybrid **4** and the clinical NF antibiotics were non‐toxic (IC_50_>100 μM). Furthermore, there was no distinctive difference in cytotoxicity between alkyl and aryl hybrids, nor did the increase in *n*‐alkyl chain length or electronegativity have a bearing on the cytotoxicity. Thus, the cytotoxic profiles of the hybrids were structure‐specific. However, it could also be observed that anchoring of the Tz moiety enhanced the basal cytotoxicity of NFT as all the hybrids were found to be consistently more toxic than this parent drug.

NFT showed poor antileishmanial activity regardless of the *Leishmania* strain considered (IC_50_>10 μM), worse than the hybrids and fellow NF antibiotics alike, hence was not identified as hit. All hybrids and reference drugs were active against *L. major* promastigotes but only **2**, **7** and **16** exhibited selective activities (SI_1_>10) against the amastigotes (IC_50_<10 μM). This intrinsic intracellular antiparasitic activity was further confirmed in the presence of host macrophage (SI_3_>10). These hybrids alongside the hit NFs, FZD and NFZ preferentially targeted the promastigotes (S_p_I_1_<0.4) hence were identified as antipromastigote hits with anti‐CL potential.

Although, predicted with drug unlikeness, the good performance of **16** against *L. major* proved otherwise. This discrepancy between theoretical and experimental data is a further confirmation that a software‐based prediction is not a true reflection of an experimental biological assay. Indeed, the prediction accuracy of the SwissADME tool has been shown to range between 72 and 94 %.[^38^, ^45^] Nevertheless, it is still a popular webtool with the predictive capacity equivalent to or better than other well‐established methods.[^38^, ^46^]

Conversely, a paucity of synthesized compounds, **1**, **4**, **6**, **8** and **15** showed activities against *L. donovani* promastigotes with only the first three presenting with intrinsic activities (SI_2_>10 and IC_50_<10 μM) against *donovani* amastigotes, which was also supported by their good safety profiles on the macrophage cells (SI_4_>10). Interestingly, hybrids **7**, **13** and **14** were inactive against the promastigotes but **7**, **13** displayed exclusive and selective activities against the amastigotes which corroborated previous reports.^[41]^ Hence, **1**, **4**, **6**, **7**, **14** alongside all reference NF antibiotics and AMB were identified as potential antileishmanial hits^[33b]^. Moreover, the propargyl derivative **1** and hybrid **4** were the most active of all (except AMB) combining submicromolar activity, IC_50_ 0.38 μM, and more than 100‐fold selective antiparasitic action against *L. donovani* amastigote in the presence of mammalian cells (HEK‐293 and THP‐1), hence qualified as potential antileishmanial leads.^[33b]^ Both compounds demonstrated up to 30‐fold higher potency than the NF antibiotics. Dejectedly, no synthesized compound was found with equal or higher potency than AMB. However, comparison of the specificity indexes SpI_2_ values presented the synthesized hybrids with a potential advantage over AMB. Indeed, the hit **6** and the lead **1** targeted the *donovani* amastigote 3‐ (S_P_I_2_ **6** 1.3 *vs*. AMB 0.4) and 20‐fold (S_P_I_2_ **1** 7.8 *vs*. AMB 0.4) more specificity, respectively than did AMB.

Among the identified hits, **1** acted more against the amastigotes (S_p_I_2_>2), whereas **4** had its action directed towards both parasite forms (0.4<S_p_I_2_<2) with a significant preference for the amastigotes.^[41b]^ The remainder was acted more as antipromastigote compounds (S_p_I<0.4).^[41b]^

In summary, the high affinity of **1** for the clinically relevant amastigote *L. donovani*, combined with its predicted favorable physicochemical and ADME features, uncovered this lead as promising candidate for further investigation into the search for new antileishmanial agents.

As all synthesized compounds were active against *L. major* promastigotes, their IC_50_ values were considered to investigate possible structure‐activity relationship (SAR) within the series. The activity in the *n*‐alkyl sub‐series (**1**–**9**) displayed a zig‐zag pattern, with a decrease from **1** to **2**, then an increase to **4** followed by another downward trajectory to **5** then an increase to **6** etc., as the chain lengthened or the lipophilicity increased.

In the aryl sub‐series, the hybrids **11**–**14** bore electron donating groups (EDGs) with the increasing order of strength being H (**11**) <Me (**12**)<*i*Pr (**13**)<*t*‐Bu (**14**). On the other hand, hybrids **15**–**20** had electron withdrawing groups (EWGs) with the increasing order of electronic effect; 4‐Br (**15**)<3‐Br (**19**)<4‐F (**17**)<2‐F 4‐Br (**16**)<4‐CF_3_ (**20**)<4‐NO_2_ (**18**). An unevenly patterned activity profile was also observed with the increasing electronic effect in this sub‐series.

Overall, the activity variation in the series was consistent with a dependence to the structure specificity of each compound rather than a specific physiochemical property. Hence, neither the alkyl chain length nor the electronic effect appeared as regulators of the activity.

## Conclusion

A series of nitrofurantoin‐triazole hybrids were synthesized in moderate yields in a three‐step process encompassing nucleophilic substitution and CuAAC reactions. Unlike, the low cytotoxic parent nitrofurantoin, the hybrids were found with mild to weak basal/general toxicity. This study uncovered several antileishmanial hits, **6**, **7** and **14**, with micromolar activities and two leads, **1** and **4**, with submicromolar activities. Hence, the hybridization strategy herein employed resulted in enhancement of the antileishmanial activity of NFT. The compound **1**, by virtue of its favorable features (easy single step synthesis, predicted oral drug‐like physicochemical properties, good antileishmanial activity and safety profile) stood out as the promising nitrofurantoin derivative for further investigation into its potential to act as an alternative to the current therapies. Future investigation of this lead will focus on its exact mechanism of action, optimization, and *in vivo* activity determination.

## Experimental Section

**Materials and methods**: NFT was purchased from Changzhou Kaixuan Chemical Co (Chunjiang, China). Anhydrous DMF (*N,N*‐dimethylformamide), TEA (trimethylamine), 1‐bromopentane, 1‐bromohexane, 1‐bromoheptane, 1‐bromooctane, 1‐bromononane, 1‐bromodecane, 1‐bromoundecane, 1‐bromododecane, benzyl bromide, 4‐bromobenzyl bromide, 1‐(2‐bromoethyl)‐4‐nitrobenzene, 1‐(bromomethyl)‐4‐nitrobenzene, 1‐(bromomethyl)‐4‐fluorobenzene, 1‐(bromomethyl)‐4‐methylbenzene, 1‐(bromomethyl)‐4‐isopropylbenzene, 1‐(bromomethyl)‐4‐(*tert*‐butyl)benzene, 4‐bromo‐1‐(bromomethyl)‐2‐fluorobenzene, 1‐bromo‐3‐(bromomethyl)benzene, 1‐(bromomethyl)‐3‐(trifluoromethyl)benzene, sodium azide, sodium ascorbate, and copper (II) sulphate were purchased from Sigma‐Aldrich (Johannesburg, South Africa). Dichloromethane (DCM), methanol (MeOH), ethanol (EtOH) and ethyl acetate (EtOAc) were purchased from ACE chemicals (Johannesburg, South Africa). All the chemicals and reagents were of reagent grade and were used without further purification.

**General procedures**: The ^1^H and ^13^C NMR spectra were recorded on a Bruker Advance™ III 600 spectrometer at a frequency of 600 MHz and 150.913 MHz, respectively, in DMSO‐*d_6_*. Chemical shifts are reported in parts per million δ (ppm), with the residual protons of the solvent as reference. The splitting pattern abbreviations are as follows: singlet (s), doublet (d), doublet of doublet (dd), doublet of doublet of doublets (ddd), doublet of triplets (dt), doublet of quartets (dq), triplet (t), triplet of doublets (td), triplet of triplets (tt), quartet of doublets (qd) and multiplet (m).

HRMS was recorded on a Bruker MicroTOF Q II mass spectrometer, equipped with an APCI or an ESI source, set at 200 °C or 180 °C, respectively, using Bruker Compass DataAnalysis 4.0 software. A full scan from 50–1500 *m/z* was performed at a capillary voltage of 4500 V, an end plate offset voltage of −500 V, with the nebulizer set at 1.6 Bar and 0.4 Bar, respectively, and a collision cell RF voltage of 100 Vpp.

Mass spectra (MS) were recorded in positive mode on a Thermo Electron LXQ™ ion trap mass spectrometer, equipped with Xcalibur 2.2 data acquisition and analysis software. The MS had an APCI source set at 300 °C, and was direct infusion with a Harvard syringe pump utilized at a flow rate of 10 μL/min. A full scan from 100–1200 amu was achieved in 1 s, with a capillary voltage of 7 V, while the corona discharge was 10 μA.

IR spectra were recorded on a Bruker Alpha‐P FTIR instrument. Thin layer chromatography (TLC) was performed, using silica gel plates (60F_254_), obtained from Merck (Johannesburg, South Africa). HPLC analyses was conducted using an Agilent 1100 series instrument equipped with a gradient pump, autosampler, diode Array UV detector. OpenLab CDS Chemstation Rev.C.01.07 SR3 data acquisition and analysis software were used (Agilent Technologies, Palo Alto, CA, USA). The column was a Venusil XBP C18 (2)‐column, 150×4.6 mm, 5 μm spherical particles, 100 Å pore size (Agela Technologies, Newark, DE, USA), The mobile phase consisted of acetonitrile and 0.1 % orthophosphoric acid in water with a linear gradient from 30 % acetonitrile to 85 % after five minutes and holding until 15 minutes before equilibrating with 30 % acetonitrile to 20 minutes. The flow rate was set at 1 mL/min and the injection volume was 1 μL. The UV signal was monitored at 365 nm.

**Syntheses**: The synthesis of the hybrids occurred in three steps, namely (a) the propargylation of NFT to give the intermediate **1**, (b) the preparation of alkyl/benzyl azides and (c) the synthesis of the hybrids from **1** and alkyl/benzyl azides using click chemistry.

**Intermediate 1**: NFT (4.20 mmol, 1.0 g) was dissolved in anhydrous DMF (10 mL), and TEA (4.41 mmol, 1.5 equiv.) was added. The solution was stirred at room temperature for 15 min. To the resulting yellow solution, propargyl bromide (4.41 mmol, 1.5 equiv.) was added, stirred at room temperature for 24 h and monitored by TLC, eluting with DCM:MeOH (19 : 1, v/v).

Upon completion, the reaction was quenched with water (20 mL) and extracted with ethyl acetate (3×20 mL). The organic layer was washed with saturated NH_4_Cl (20 mL) and water (20 mL). The solution was spun to dryness *in vacuo*, and the resulting paste was purified by recrystallization in ethanol/water to afford the intermediate, **1** as a yellow powder. The characterization data of **1** are presented in the Supporting Information.

**Hybrids 2**–**20**: Intermediate **1** (1.81 mmol, 0.5 g, 1 equiv.) was dissolved DMSO:water (10 mL) (4 : 1, v/v). To the resulting solution, the appropriate 1‐azidoalkane/benzene (1.5 equiv.) was added with stirring. Catalytic amounts of β‐cyclodextrin (0.02 equiv.), sodium ascorbate (0.3 equiv.) and copper sulphate pentahydrate (0.1 equiv.) were then added in sequence and the stirring was continued for 12 h. Afterwards, an additional portion of sodium ascorbate (0.3 equiv.) was added and the mixture was stirred at room temperature for another 48 h, monitored by TLC, eluting with DCM:MeOH (19 : 1, v/v). Upon completion, the reaction was quenched with water (20 mL) and extracted with ethyl acetate (3×20 mL). The organic layer was washed with saturated NH_4_Cl (30 mL) and water (30 mL). The solution was spun to dryness *in vacuo* and the resulting residue was purified by recrystallization in ethanol/water to afford the desired hybrid.

The characterization data of all hybrids are presented in the Supporting Information.

## In vitro Biological Assays

**Antipromastigote assay**: The antipromastigote activity of synthesized compounds was evaluated as described previously by Mangwegape *et al*.^[47]^ using *L. donovani* 9515 (MHOM/IN/95/9515) and *L. major* IR‐173 (MHOM/IR/‐173). All compounds were first screened for >70 % growth inhibition at 10 μM^[40]^ and qualifying compounds were further used for IC_50_ determinations. The assay description is available in the Supporting Information.

**Anti‐amastigote assay**: The activities of synthesized compounds against the intramacrophage parasites of the three *Leishmania* strains were evaluated using a modified, resazurin‐based method of Jain *et al*.^[48]^ and Njanpa *et al*.^[49]^ Suspension cultures of human acute monocytic leukemia (THP‐1, Cellonex, South Africa) cells were maintained in RPMI‐1640 medium (Sigma Aldrich) supplemented with 10 % FBS and 1 % penicillin‐streptomycin, at 37 °C and 5 % CO_2_ in a humidified atmosphere. Duplicate 96 well plates (for respective anti‐amastigote and cytotoxicity assays) were seeded with 200 μL of a 2.5×10^5^ cells/mL suspension treated with 25 ng/mL phorbol 12‐myristate 13‐acetate (PMA), followed by 48‐hour incubation to promote differentiation into adherent macrophages.

For the anti‐amastigote assay, differentiated plates were carefully washed with PBS, followed by the addition of 200 μL of stationary phase promastigotes in RPMI 1640 medium with 2 % FBS. An MOI of 30 : 1 was used for all three *Leishmania* strains. The parasite‐treated plates were incubated for 24 hours at 32 °C (*L. major*) or 37 °C (*L. donovani*) and 5 % CO_2_ to promote infection of the macrophages. The wells were then washed four times with PBS to remove extracellular parasites, followed by treatment with 200 μL of: (i) amphotericin B (Sigma Aldrich) (positive control); (ii) growth medium and solvent (negative control to compensate for possible solvent effects); (iii) 10 μM of compound for activity screening; (iv) growth medium with 7 two‐fold dilution concentrations of 10 μM compounds for IC_50_ determination. Blanks were represented by growth medium without cells, as well as parasite‐free THP‐1 cells. The treated plates were incubated for 72 hours.

After incubation, the plates were gently washed three times with PBS to remove any remaining extracellular parasites. The wells were then treated with 20 μL of 0.05 % sodium dodecyl sulphate in PBS for 30 seconds to lyse the host macrophages. Lysis was terminated by adding 180 μL promastigote growth medium with 10 % FBS. To initiate the resazurin assay, 10 μL of resazurin solution (0.025 % in PBS) was added to all wells and the plates were incubated for 24 hours at 32 °C (*L. major*) or 37 °C (*L. donovani*) and 5 % CO_2_. Absorbance measurements, calculations and IC_50_ determinations were performed as described for the antipromastigote assay (Supporting Information).

All compounds were first screened for growth inhibition >60 % at 10 μM^[41b]^ and the qualifying compounds further proceeded for IC_50_ determinations.

**Cytotoxicity assay**: Human embryonal kidney (HEK‐293) cells (ATCC CRL 1573) were used for basal cytotoxicity of the synthesized compounds possessing antileishmanial activity by adopting the resazurin assay, as previously described.^[47]^

**Macrophage toxicity**: Toxicity of the compounds on the differentiated host THP‐1 cells were also evaluated. As mentioned, duplicate plates of differentiated THP‐1 cells were prepared during the anti‐amastigote assay. These plates were incubated for 72 hours to differentiate, instead of replacing the growth medium with parasite‐containing medium after 48 hours. The plates were then treated with 200 μL of: (i) amphotericin B (Sigma Aldrich) (positive control); (ii) growth medium and solvent (negative control to compensate for possible solvent effects); (iii) 7 two‐fold dilution concentrations of 100 μM compounds for IC_50_ determination. Blanks were growth medium without cells. The treated plates were incubated for 72 hours, followed by the addition of 50 μL of resazurin solution (0.01 % in PBS) to each well and 24‐hour incubation. Further data analysis, calculations and IC_50_ determinations were identical to that of the antileishmanial assays.

## Disclaimer

Any opinions, findings and conclusions, or recommendations expressed in this material are those of the authors and therefore the NRF does not accept any liability in regard thereto.

The following reagents were obtained through BEI Resources, NIAID, NIH: *Leishmania donovani*, Strain 9515 (MHOM/IN/95/9515), NR‐48822; *Leishmania major*, Strain IR173 (MHOM/IR/‐173), NR‐48816.

## Ethics

Ethics approval for this study was obtained from the Human Research Ethics Committee of the North‐West University (NWU‐00445‐20‐A1).

## Conflict of interest

The authors declare no conflict of interest.

1

## Supporting information

As a service to our authors and readers, this journal provides supporting information supplied by the authors. Such materials are peer reviewed and may be re‐organized for online delivery, but are not copy‐edited or typeset. Technical support issues arising from supporting information (other than missing files) should be addressed to the authors.

Supporting Information

## Data Availability

The data that support the findings of this study are available in the supplementary material of this article.

## References

[1] WHO, *Leishmaniasis*, https://www.who.int/news-room/fact-sheets/detail/leishmaniasis [Date of access: 08 January 2022], **2022**.

[2a] K. Cheraghipour , A. Marzban , B. Ezatpour , S. Khanizadeh , J. Koshki , AIMS J. 2018, 3, 561–578;

[2b] B. M. Roatt , J. M. de Oliveira Cardoso , R. C. F. De Brito , W. Coura-Vital , R. D. de Oliveira Aguiar-Soares , A. B. Reis , Appl. Microbiol. Biotechnol. 2020, 104, 8965–8977. 32875362 10.1007/s00253-020-10856-w

[3] D. K. Deep , R. Singh , V. Bhandari , A. Verma , V. Sharma , S. Wajid , S. Sundar , V. Ramesh , J. C. Dujardin , P. Salotra , PLoS Neglected Trop. Dis. 2017, 11, 1–16. 10.1371/journal.pntd.0005641 PMC5470736 28575060

[4] Z. Zhang , L. Zhou , N. Xie , E. C. Nice , T. Zhang , Y. Cui , C. Huang , Sign. Transduct. Target. Ther. 2020, 5, 113. 10.1038/s41392-020-00213-8 PMC7331117 32616710

[5] S. M. Corsello , J. A. Bittker , Z. Liu , J. Gould , P. McCarren , J. E. Hirschman , S. E. Johnston , A. Vrcic , B. Wong , M. Khan , J. Asiedu , R. Narayan , C. C. Mader , A. Subramanian , T. R. Golub , Nat. Med. 2017, 23, 405–408. 28388612 10.1038/nm.4306 PMC5568558

[6] V. Ivasiv , C. Albertini , A. E. Gonçalves , M. Rossi , M. L. Bolognesi , Curr. Top. Med. Chem. 2019, 19, 1694–1711. 31237210 10.2174/1568026619666190619115735

[7] R. L. Charlton , B. Rossi-Bergmann , P. W. Denny , P. G. Steel , Parasitology 2018, 145, 219–236. 28805165 10.1017/S0031182017000993 PMC5964475

[8] M. Montalto de Mecca , E. G. Diaz , J. A. Castro , Toxicol. Lett. 2002, 136, 1–8. 12368051 10.1016/s0378-4274(02)00238-2

[9] S. Jeganathan , L. Sanderson , M. Dogruel , J. Rodgers , S. Croft , S. Thomas , J. Pharmacol. Exp. Ther. 2010, 336, 506–515. 21057057 10.1124/jpet.110.172981 PMC3033724

[10a] S. C. Petri e Silva , F. Palace-Berl , L. C. Tavares , S. R. Soares , J. A. Lindoso , Exp. Parasitol. 2016, 163, 68–75; 26795261 10.1016/j.exppara.2016.01.007

[10b] M. E. Walsh , E. M. Naudzius , S. J. Diaz , T. W. Wismar , M. Martchenko Shilman , D. Schulz , PLoS Neglected Trop. Dis. 2020, 14, e0007790. 10.1371/journal.pntd.0007790 PMC7094864 32168320

[11] D. A. Morrison , Trends Parasitol. 2009, 25, 375–382. 19635681 10.1016/j.pt.2009.05.010

[12] Y. Wang , J. P. Gray , V. Mishin , D. E. Heck , D. L. Laskin , J. D. Laskin , Free Radical Biol. Med. 2008, 44, 1169–1179. 18206659 10.1016/j.freeradbiomed.2007.12.013 PMC5793909

[13] L. Zhou , H. Ishizaki , M. Spitzer , K. L. Taylor , N. D. Temperley , S. L. Johnson , P. Brear , P. Gautier , Z. Zeng , A. Mitchell , V. Narayan , E. M. McNeil , D. W. Melton , T. K. Smith , M. Tyers , N. J. Westwood , E. E. Patton , Chem. Biol. 2012, 19, 883–892. 22840776 10.1016/j.chembiol.2012.05.017 PMC3684953

[14] M. D. Roldán , E. Pérez-Reinado , F. Castillo , C. Moreno-Vivián , FEMS Microbiol. Rev. 2008, 32, 474–500. 18355273 10.1111/j.1574-6976.2008.00107.x

[15] A. Ryan , Br. J. Pharmacol. 2017, 174, 2161–2173. 27487252 10.1111/bph.13571 PMC5481658

[16a] P. Wardman , Environ. Health Perspect. 1985, 64, 309–320; 3830700 10.1289/ehp.8564309 PMC1568617

[16b] J. M. Gutteridge , FEBS Lett. 1985, 185, 19–23. 2987038 10.1016/0014-5793(85)80732-8

[17] B. E. Blass, in *Basic Principles of Drug Discovery and Development* (Ed.: B. E. E. Blass), Academic Press, Boston, **2015**, pp. 499–529.

[18] X. Y. Teoh , F. N. Bt Mahyuddin , W. Ahmad , S. Y. Chan , Pharm. Dev. Technol. 2020, 25, 245–251. 31690150 10.1080/10837450.2019.1689401

[19] A. Bains , D. Buna , N. A. Hoag , Can. Pharm. J. 2009, 142, 248–252.

[20a] T. B. Cassamale , E. C. Costa , D. B. Carvalho , N. S. Cassemiro , C. C. Tomazela , M. C. S. Marques , M. Ojeda , M. F. C. Matos , S. Albuquerque , C. C. P. Arruda , A. C. M. Baroni , J. Braz. Chem. Soc. 2016, 27, 1217–1228;

[20b] E. O. J. Porta , S. N. Jäger , I. Nocito , G. I. Lepesheva , E. C. Serra , B. L. Tekwani , G. R. Labadie , MedChemComm 2017, 8, 1015–1021. 28993794 10.1039/c7md00008a PMC5629980

[21a] T. B. de Souza , I. S. Caldas , F. R. Paula , C. C. Rodrigues , D. T. Carvalho , D. F. Dias , Chem. Biol. Drug Des. 2020, 95, 124–129; 31569301 10.1111/cbdd.13628

[21b] L.-f. Mao , Y.-w. Wang , J. Zhao , G.-q. Xu , X.-j. Yao , Y.-M. Li , Front. Pharmacol. 2020, 11, 579024. 33101032 10.3389/fphar.2020.579024 PMC7555427

[22] S. Zhang , Z. Xu , C. Gao , Q.-C. Ren , L. Chang , Z.-S. Lv , L.-S. Feng , Eur. J. Med. Chem. 2017, 138, 501–513. 28692915 10.1016/j.ejmech.2017.06.051

[23] V. Ji Ram , A. Sethi , M. Nath , R. Pratap , in The Chemistry of Heterocycles (Eds.: V. Ji Ram , A. Sethi , M. Nath , R. Pratap ), Elsevier, 2019, pp. 19–92.

[24] D. Kumar , Beena , G. Khare , S. Kidwai , A. K. Tyagi , R. Singh , D. S. Rawat , Eur. J. Med. Chem. 2014, 81, 301–313. 24852277 10.1016/j.ejmech.2014.05.005

[25a] K. Maji , M. Abbasi , D. Podder , R. Datta , D. Haldar , ChemistrySelect 2018, 3, 10220–10225;

[25b] C. P. Kaushik , R. Luxmi , J. Heterocycl. Chem. 2020, 57, 2400–2409;

[25c] C.-M. Horn , J. Aucamp , F. J. Smit , R. Seldon , A. Jordaan , D. F. Warner , D. D. N'Da , Med. Chem. Res. 2020, 29, 1387–1399.

[26] J. D. Conklin , Antibiot. Chemother. 1978, 25, 233–252. 352255 10.1159/000401065

[27] M. N. Martinez , G. L. Amidon , J. Clin. Pharmacol. 2002, 42, 620–643. 12043951 10.1177/00970002042006005

[28] P. Cilliers , R. Seldon , F. J. Smit , J. Aucamp , A. Jordaan , D. F. Warner , D. D. N′Da , Chem. Biol. Drug Des. 2019, 94, 1518–1536. 31033220 10.1111/cbdd.13534

[29] J.-A. Shin , Y.-G. Lim , K.-H. Lee , J. Org. Chem. 2012, 77, 4117–4122. 22448725 10.1021/jo3000095

[30] M. J. Waring , Expert Opin. Drug Discovery 2010, 5, 235–248. 10.1517/17460441003605098 22823020

[31] H. van de Waterbeemd , E. Gifford , Nat. Rev. Drug Discovery 2003, 2, 192–204. 12612645 10.1038/nrd1032

[32] C. A. Lipinski , F. Lombardo , B. W. Dominy , P. J. Feeney , Adv. Drug Delivery Rev. 2001, 46, 3–26. 10.1016/s0169-409x(00)00129-0 11259830

[33a] R. J. A. Goodwin , J. Bunch , D. F. McGinnity , in Advances in Cancer Research, Vol. 134 (Eds.: R. R. Drake , L. A. McDonnell ), Academic Press, 2017, pp. 133–171; 10.1016/bs.acr.2016.11.005 28110649

[33b] K. Katsuno , J. N. Burrows , K. Duncan , R. H. Van Huijsduijnen , T. Kaneko , K. Kita , C. E. Mowbray , D. Schmatz , P. Warner , B. Slingsby , Nat. Rev. Drug Discovery 2015, 14, 751–758. 26435527 10.1038/nrd4683

[34] J. S. Delaney , J. Chem. Inf. Comput. Sci. 2004, 44, 1000–1005. 15154768 10.1021/ci034243x

[35] J. Ali , P. Camilleri , M. B. Brown , A. J. Hutt , S. B. Kirton , J. Chem. Inf. Model. 2012, 52, 420–428. 22196228 10.1021/ci200387c

[36] D. F. Veber , S. R. Johnson , H.-Y. Cheng , B. R. Smith , K. W. Ward , K. D. Kopple , J. Med. Chem. 2002, 45, 2615–2623. 12036371 10.1021/jm020017n

[37] A. Daina , V. Zoete , ChemMedChem 2016, 11, 1117–1121. 27218427 10.1002/cmdc.201600182 PMC5089604

[38] A. Daina , O. Michielin , V. Zoete , Sci. Rep. 2017, 7, 42717. 28256516 10.1038/srep42717 PMC5335600

[39a] R. Lira , S. Sundar , A. Makharia , R. Kenney , A. Gam , E. Saraiva , D. Sacks , J. Infect. Dis. 1999, 180, 564–567; 10395884 10.1086/314896

[39b] J. E. Potvin , P. Leprohon , M. Queffeulou , S. Sundar , M. Ouellette , Clin. Infect. Dis. 2021, 72, e526-e532. 32827255 10.1093/cid/ciaa1236 PMC8130028

[40] J. L. Siqueira-Neto , O.-R. Song , H. Oh , J.-H. Sohn , G. Yang , J. Nam , J. Jang , J. Cechetto , C. B. Lee , S. Moon , A. Genovesio , E. Chatelain , T. Christophe , L. H. Freitas-Junior , PLoS Neglected Trop. Dis. 2010, 4, e675-e675. 10.1371/journal.pntd.0000675 PMC2864270 20454559

[41a] H. L. Callahan , A. C. Portal , R. Devereaux , M. Grogl , Antimicrob. Agents Chemother. 1997, 41, 818–822; 9087496 10.1128/aac.41.4.818 PMC163801

[41b] G. De Muylder , K. K. Ang , S. Chen , M. R. Arkin , J. C. Engel , J. H. McKerrow , PLoS Neglected Trop. Dis. 2011, 5, e1253; 10.1371/journal.pntd.0001253 PMC3139667 21811648

[41c] M. Vermeersch , R. I. da Luz , K. Toté , J. P. Timmermans , P. Cos , L. Maes , Antimicrob. Agents Chemother. 2009, 53, 3855–3859. 19546361 10.1128/AAC.00548-09 PMC2737839

[42] F. P. Guengerich , Drug Metab. Pharmacokinet. 2011, 26, 3–14. 20978361 10.2133/dmpk.dmpk-10-rv-062 PMC4707670

[43] E. A. Adewusi , P. Steenkamp , G. Fouche , V. Steenkamp , Nat. Prod. Commun. 2013, 8, 1213–1216. 24273848

[44] S. Liu , M. Su , S. J. Song , J. H. Jung , Mar. Drugs 2017, 15, 329. 10.3390/md15100329 PMC5666435 29064452

[45] D. L. Roman , M. Roman , C. Som , M. Schmutz , E. Hernandez , P. Wick , T. Casalini , G. Perale , V. Ostafe , A. Isvoran , Front. Bioeng. Biotechnol. 2019, 7, 214. 31552240 10.3389/fbioe.2019.00214 PMC6743017

[46] B. Bakchi , A. D. Krishna , E. Sreecharan , V. B. J. Ganesh , M. Niharika , S. Maharshi , S. B. Puttagunta , D. K. Sigalapalli , R. R. Bhandare , A. B. Shaik , J. Mol. Struct. 2022, 1259, 132712.

[47] D. K. Mangwegape , N. H. Zuma , J. Aucamp , D. D. N'Da , Arch. Pharm. 2021, 354, e2000280. 10.1002/ardp.202000280 33491807

[48] S. K. Jain , R. Sahu , L. A. Walker , B. L. Tekwani , J. Visualization 2012, 70, e4054.

[49] C. A. N. Njanpa , S. C. N. Wouamba , L. R. T. Yamthe , D. Dize , B. M. T. Tchatat , P. V. F. Tsouh , M. N. Pouofo , J. B. Jouda , B. L. Ndjakou , N. Sewald , S. F. Kouam , F. F. Boyom , BMC Complem. Med. Therap. 2021, 21, art. #106. 10.1186/s12906-021-03279-1 PMC8011081 33789661

