# Supplementary material for: Synthesis, in vitro Antileishmanial Efficacy and Hit/Lead Identification of Nitrofurantoin‐Triazole Hybrids
Source: ChemMedChem. 2022 May 2;17(10):e202200023. doi: 10.1002/cmdc.202200023 (PMC9322565; doi:10.1002/cmdc.202200023)
Supplement: Supplementary file 1 — Supporting Information [file CMDC-17-0-s001.pdf]

ChemMedChem

Supporting Information

Synthesis, *in vitro* Antileishmanial Efficacy and Hit/Lead Identification of Nitrofurantoin-Triazole Hybrids

Nonkululeko H. Zuma, Janine Aucamp, Maryna Viljoen, and David D. N'Da*

(E)-1-{[(5-Nitrofuran-2-yl)methylene]amino}-3-(prop-2-yn-1-yl)imidazolidine-2,4-dione, ***1***

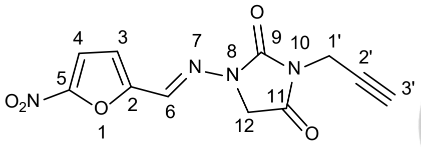

Yield: 1.06 g (91%); mp: 136.1-136.2 °C (EtOH/H_2O). IR (ATR) ν_{max}/cm^{-1}: 2138 (C≡C), 1779 (C=O), 1719 (C=O), 1523 (NO_2). ^1H NMR (600 MHz, DMSO) δ (ppm): 7.89 (s, 1H, H-6), 7.80 (d, *J* = 3.8 Hz, 1H, H-4), 7.20 (d, *J* = 3.8 Hz, 1H, H-3), 4.49 (s, 2H, H-12), 4.29 (d, *J* = 2.5 Hz, 2H, H-1'), 3.31 (t, *J* = 2.5 Hz, 1H, H-3'). ^{13}C NMR (151 MHz, DMSO) δ (ppm): 166.9 (C-11), 152.3 (C-9, -5), 152.0 (C-2), 132.6 (C-6), 115.5 (C-4), 115.1 (C-3), 78.1 (C-2'), 74.8 (C-3'), 49.9 (C-12), 28.20 (C-1'). Purity 88%. HRMS *m/z* $[M+H]^+$: 277.0547 (calcd. for $C_{11}H_9N_4O_5^+$ 277.0573).

^{1}H NMR

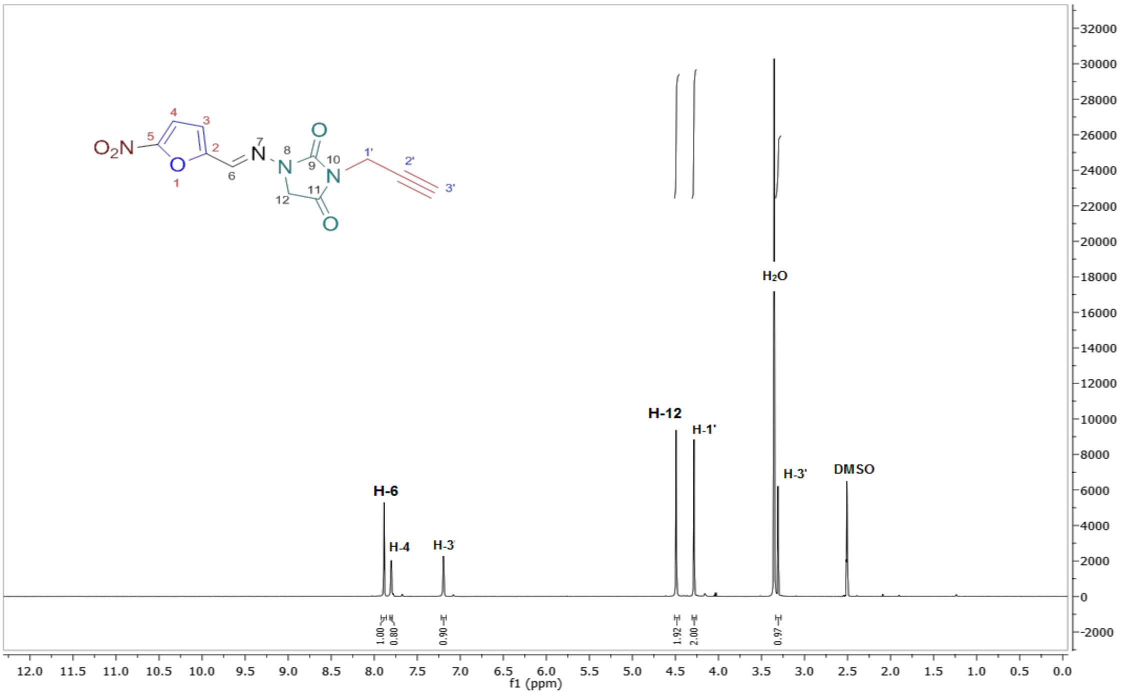

^{13}C NMR

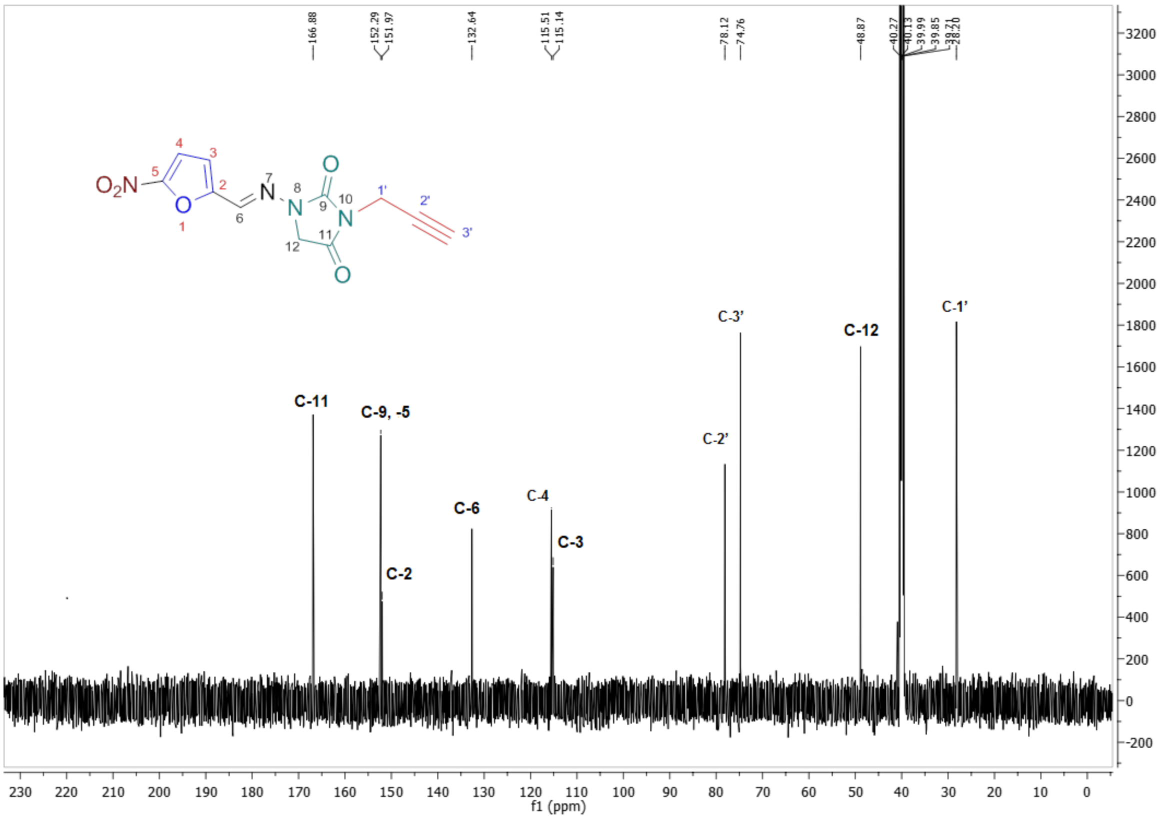

HRMS

Acquisition Parameter

Source Type	APCI	Ion Polarity	Positive	Set Nebulizer	1.6 Bar
Focus	Not active	Set Capillary	4500 V	Set Dry Heater	200 °C
Scan Begin	50 m/z	Set End Plate Offset	-500 V	Set Dry Gas	8.0 l/min
Scan End	1500 m/z	Set Collision Cell RF	100.0 Vpp	Set Divert Valve	Waste

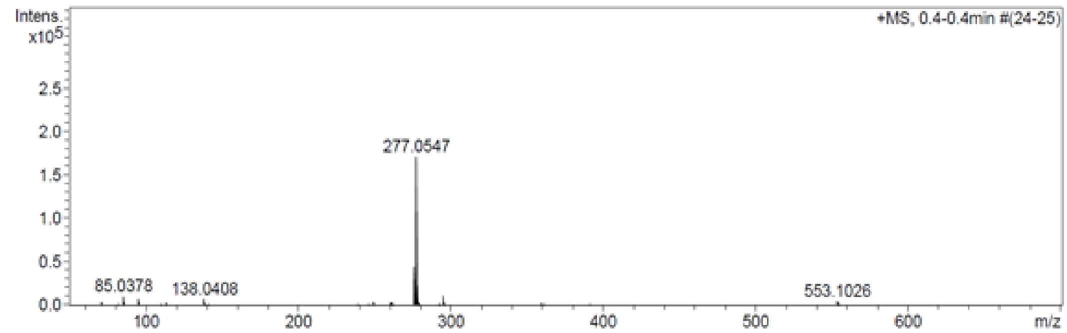

HPLC Purity

SHIMADZU LabSolutions Analysis Report

<Sample Information>

Sample Name	: David NDa S1		
Sample ID	: David NDa S1		
Data Filename	: David NDa S1_004.lcd		
Method Filename	: PURITY non-polar.lcm		
Batch Filename	: David NDa.lcb		
Vial #	: 1-2	Sample Type	: Unknown
Injection Volume	: 1 uL		
Date Acquired	: 29/03/2022 09:13:30	Acquired by	: System Administrator
Date Processed	: 31/03/2022 08:04:35	Processed by	: System Administrator

<Chromatogram>

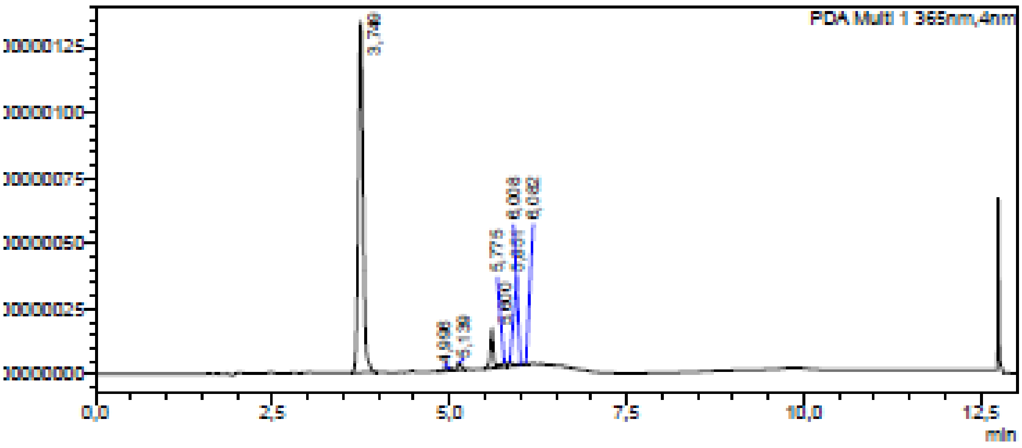

<Peak Table>

PDA Ch1 365nm

Peak#	Ret. Time	Area	Area%
1	3,749	709016	88,044
2	4,996	9305	1,155
3	5,139	12891	1,601
4	5,600	53400	6,631
5	5,775	8986	1,116
6	5,851	7587	0,942
7	6,008	2598	0,323
8	6,082	1512	0,188
Total		805294	100,000

Synthesis of alkyl/benzyl azides

These intermediates were prepared according to Cilliers et al. (2019) as follows:

Alkyl/benzyl bromide (1.0 eq.) were dissolved in DMSO (15 mL) together with sodium azide (NaN_3, 1.5 eq.). The reaction mixture was stirred at room temperature overnight. The resulting reaction mixture was diluted with water (30 mL) and the aqueous phase extracted with diethyl ether (3 x 40 mL). The combined organic layers were washed with brine (3 x 50 ml) and dried over $MgSO_4$. Removal of the solvent *in vacuo* resulted in the target intermediate as a clear oil which was used without further purification nor spectroscopic characterization.

P. Cilliers, R. Seldon, F.J. Smit, J. Aucamp, A. Jordaan, D.F. Warner, D.D. N'Da, Design, synthesis and antimycobacterial activity of novel ciprofloxacin derivatives, *Chem Biol Drug Des.* 2019, **94**,1518-1536.

Hybrids 2 - 20

(E)-1-{[(5-Nitrofuran-2-yl)methylene]amino}-10-[(4-pentyl-1H-1,2,3-triazol-1-yl) methyl]imidazolidine-9,11-dione, **2**

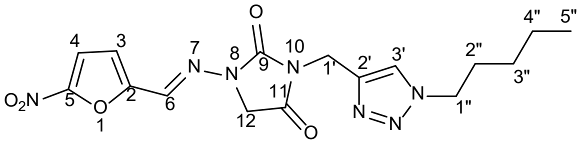

The reaction of **1** with 1-azidopentane yielded **2** as a fine brownish yellow powder, 351 mg (50%), mp: 105-106.0 °C (EtOH/H_2O). IR (ATR) ν_{max}/cm^{-1}: 3139 (C-H, alkene), 2925 (C-H, alkane), 1785 (C=O), 1712 (C=O), 1344 (C-N). ^{1}H NMR (600 MHz, DMSO) δ (ppm): 8.08 (s, 1H, H-6), 7.89 (s, 1H, H-3'), 7.80 (d, *J* = 3.9 Hz, H-4), 7.18 (d, *J* = 3.9 Hz, 1H, H-3), 4.73 (s, 2H, H-1'), 4.48 (s, 2H, H-12), 4.32 (t, *J* = 7.2 Hz, 2H, H-1"), 1.84 – 1.73 (m, 2H, H-2"), 1.35 – 1.14 (m, 4H, H-3", -4"), 0.89 – 0.80 (m, 3H, H-5"). ^{13}C NMR (151 MHz, DMSO) δ (ppm): 167.3 (C-11), 152.9 (C-9), 152.3 (C-5), 152.1 (C-2), 132.4 (C-6, -2'), 123.8 (C-3'), 115.3 (C-4), 115.2 (C-3), 49.8 (C-1"), 48.8 (C-1'), 39.7 (C-2"), 39.6 (C-3"), 22.0 (C-4"), 14.3 (C-5"). Purity 94%. HRMS *m/z* [M+H]$^+$: 390.1496 (calcd. for $C_{16}H_{20}N_7O_5^+$ 390.1526).

IR

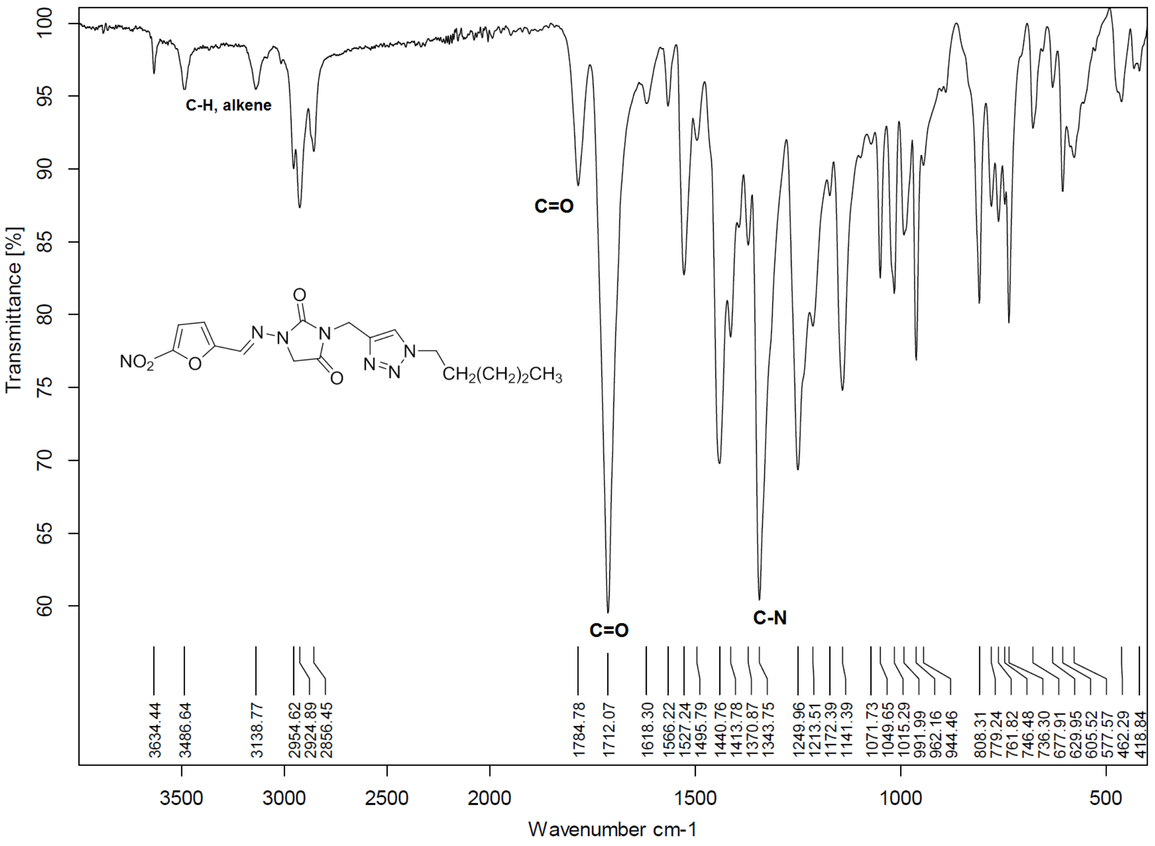

^{1}H NMR

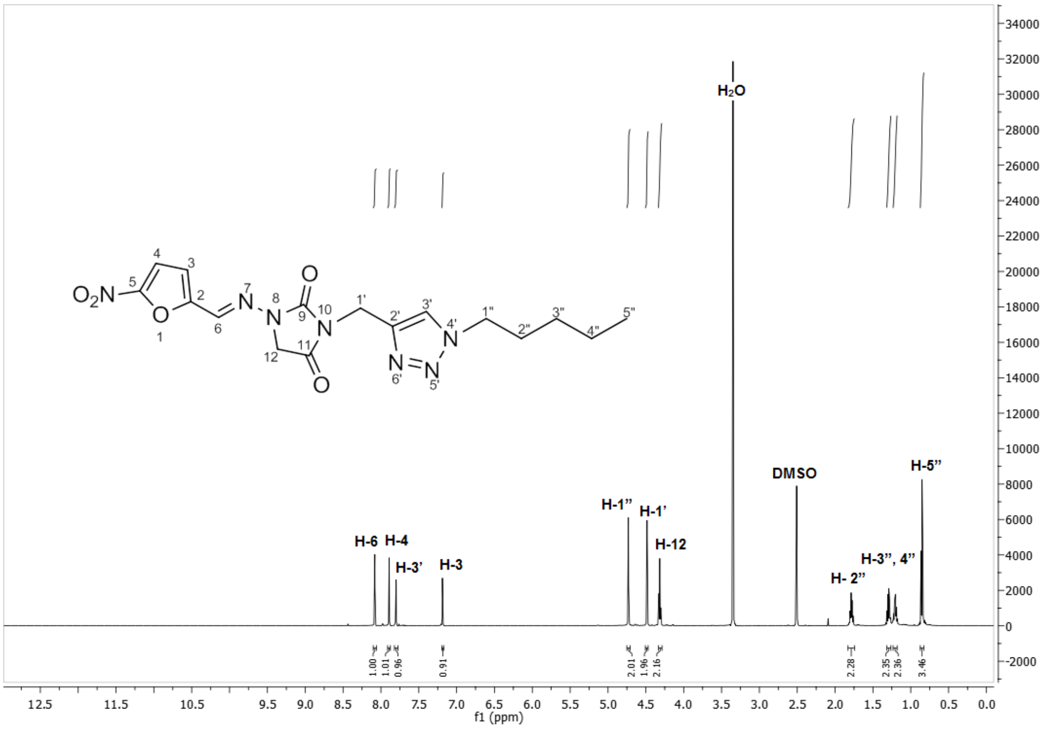

H₂O
DMSO
H-5"
H-1"
H-1'
H-12
H-6
H-4
H-3'
H-3
H- 2"
H-3", 4"
1.00
1.01
0.96
0.91
2.01
1.96
2.16
2.28
2.35
2.36
3.46
f1 (ppm)

^{13}C NMR

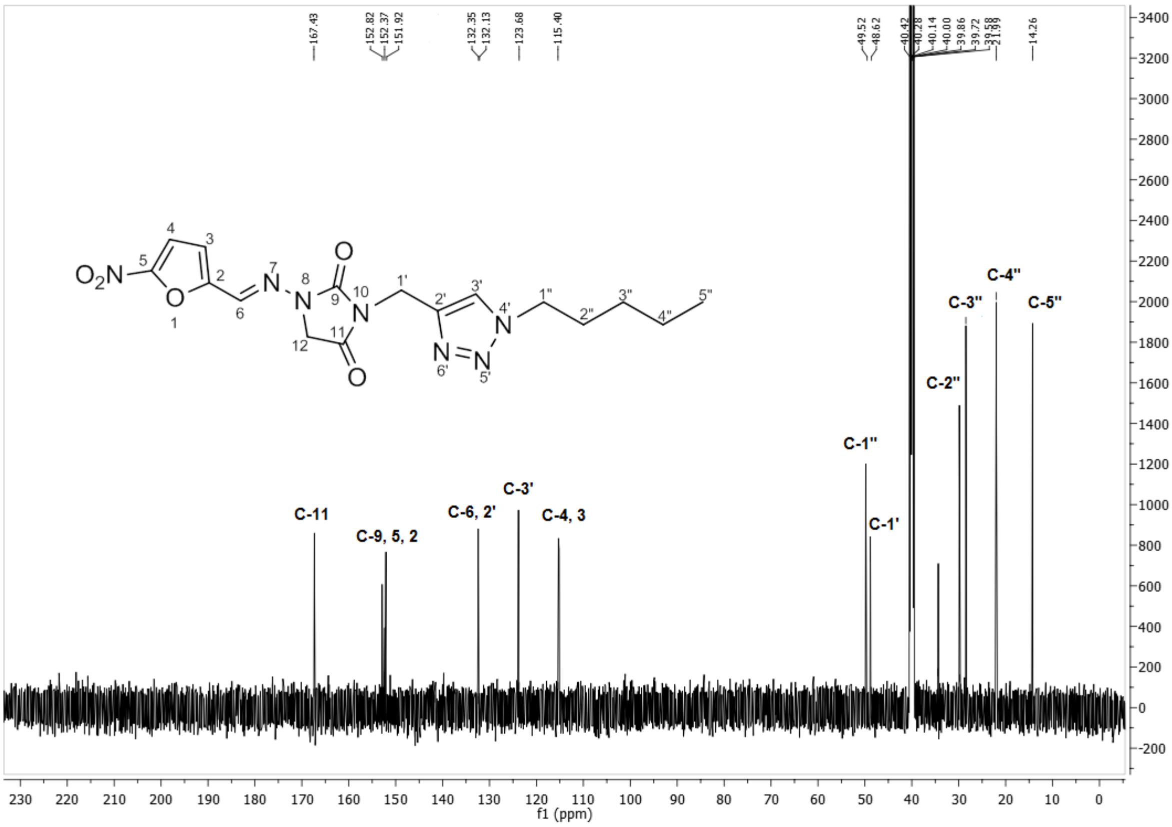

167.43
152.82
152.37
151.92
132.35
132.13
123.68
115.40
49.52
48.62
40.42
40.28
40.14
40.00
39.86
39.72
39.58
21.99
14.26
C-11
C-9, 5, 2
C-6, 2'
C-3'
C-4, 3
C-1"
C-1'
C-2"
C-3"
C-4"
C-5"
f1 (ppm)

HRMS

Acquisition Parameter

Source Type	APCI	Ion Polarity	Positive	Set Nebulizer	1.6 Bar
Focus	Not active	Set Capillary	4500 V	Set Dry Heater	200 °C
Scan Begin	50 m/z	Set End Plate Offset	-500 V	Set Dry Gas	8.0 l/min
Scan End	1500 m/z	Set Collision Cell RF	100.0 Vpp	Set Divert Valve	Waste

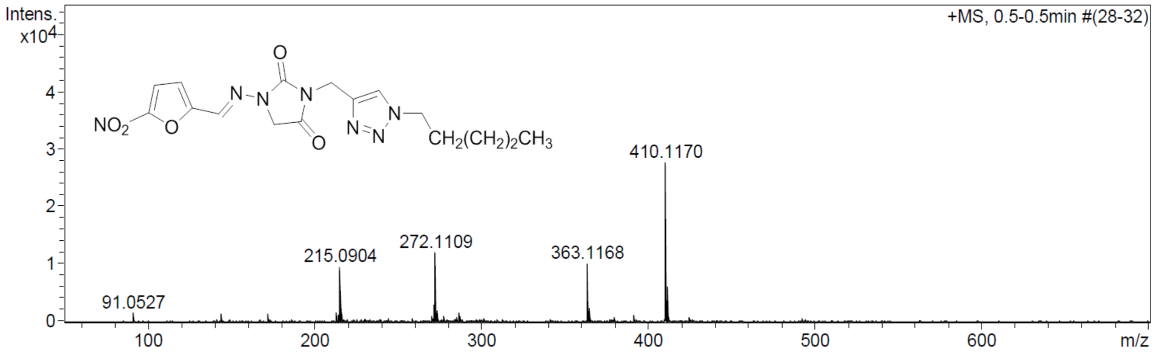

HPLC Purity

2022/03/31 08:22:55 Page 1 / 1

Analysis Report

SHIMADZU LabSolutions

<Sample Information>

Sample Name	: David NDa S2		
Sample ID	: David NDa S2		
Data Filename	: David NDa S2_005.lcd		
Method Filename	: PURITY non-polar.lcm		
Batch Filename	: David NDa.lcb		
Vial #	: 1-3	Sample Type	: Unknown
Injection Volume	: 1 uL		
Date Acquired	: 29/03/2022 09:26:51	Acquired by	: System Administrator
Date Processed	: 29/03/2022 09:39:52	Processed by	: System Administrator

<Chromatogram>

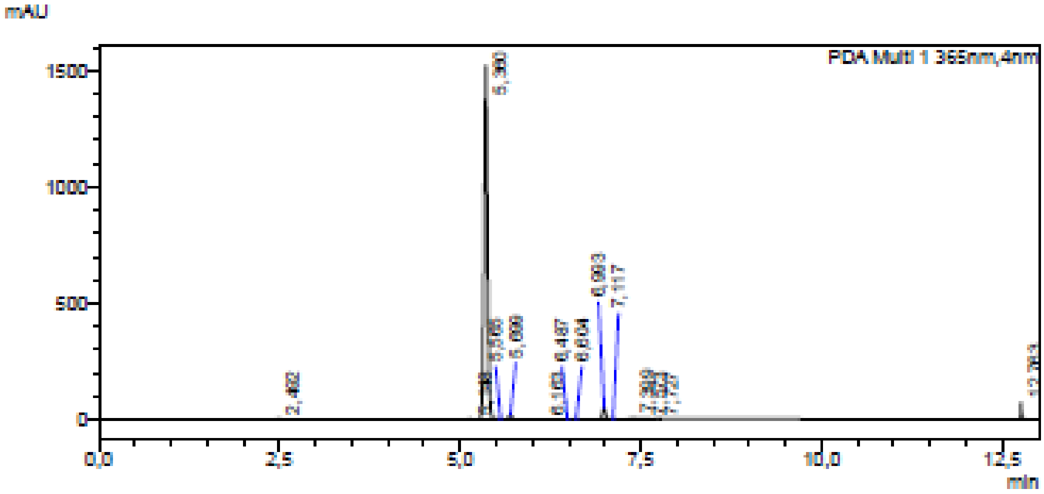

<Peak Table>

PDA Ch1 365nm

Peak#	Ret. Time	Area	Area%
1	2,492	10150	0,184
2	5,148	8292	0,150
3	5,360	5173497	93,769
4	5,565	10949	0,198
5	5,699	56063	1,016
6	6,163	1340	0,024
7	6,487	4985	0,090
8	6,604	6837	0,124
9	6,993	145191	2,632
10	7,117	4706	0,085
11	7,399	11526	0,209
12	7,573	6900	0,125
13	7,727	1988	0,036
14	12,763	74851	1,357
Total		5517276	100,000

C:\LabSolutions\Data\Project1\PURITY\David NDa S2_005.lcd

(E)-1-{[(5-Nitrofuran-2-yl)methylene]amino}-10-[(4-hexyl-1H-1,2,3-triazol-1-yl)methyl]imidazolidine-9,11-dione, ***3***

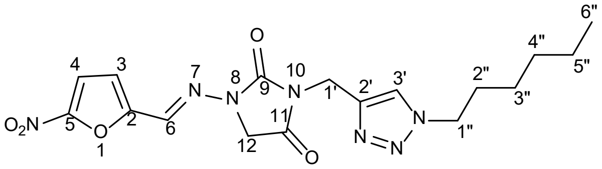

The reaction of **1** with 1-azidohexane yielded **3** as a dark orange powder, 583 mg (80%), mp: 123.8-124.6 °C (EtOH/H_2O). IR (ATR) ν_{max}/cm^{-1}: 2918 (C-H, alkane), 2850 (C-H, C-12), 1785 (C=O), 1729 (C=O), 1340 (C-N). ^{1}H NMR (600 MHz, DMSO) δ (ppm): 8.08 (s, 1H, H-6), 7.89 (s, 1H, H-3') 7.80 (d, *J* = 3.9 Hz, 1H, H-4), 7.19 (d, *J* = 3.9 Hz, 1H, H-3), 4.71 (t, *J* = 6.8 Hz, 2H, H-1"), 4.48 (s, 2H, H-12), 4.32 (s, 2H, H-1'), 1.77 (m, 2H, H- 2"), 1.27 – 1.22 (m, 6H, H-3"…-5"), 0.85 – 0.80 (m, 3H, H-6"). ^{13}C NMR (151 MHz, DMSO) δ (ppm): 167.3 (C-11), 152.9 (C-9), 152.3 (C-5), 152.1 (C-2), 132.4 (C-6, 2'), 123.8 (C-3'), 115.3 (C-4), 115.2 (C-3), 49.8 (C-1"), 48.8 (C-1'), 39.7 (C-2"), 39.6 (C-3"), 26.0 (C-4") 22.4 (C-5"), 14.3 (C-6"). Purity 93%. HRMS *m/z* [M+H]$^+$: 404.1637 (calcd. for $C_{17}H_{22}N_7O_5^+$ 404.1682).

IR

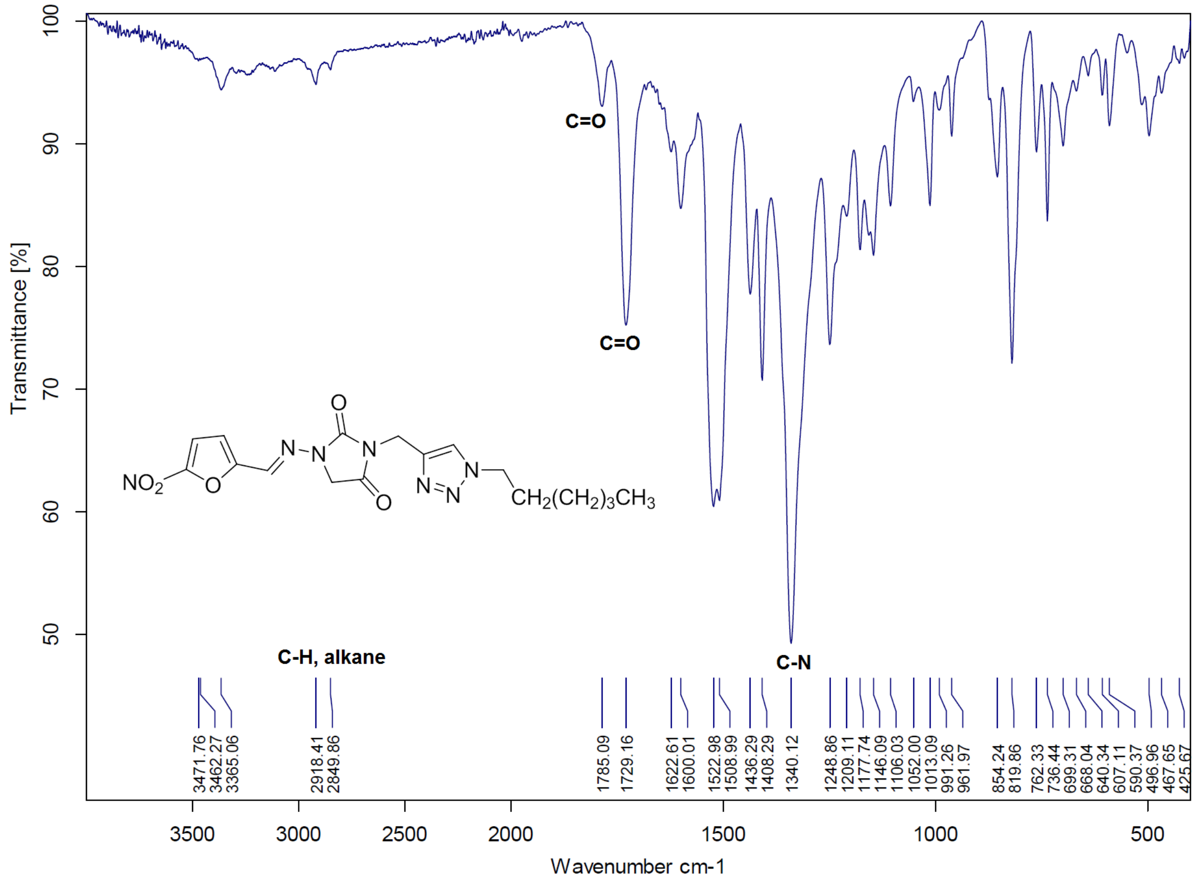

^{1}H NMR

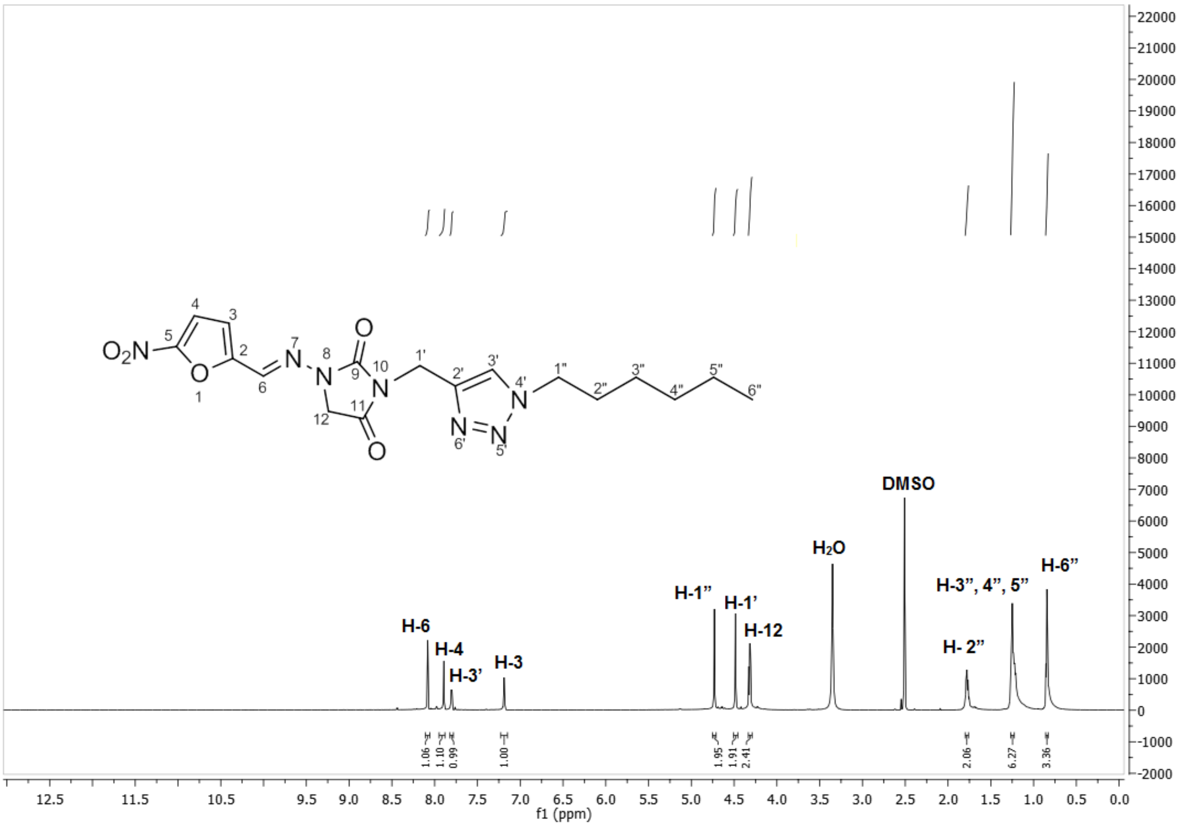

^{13}C NMR

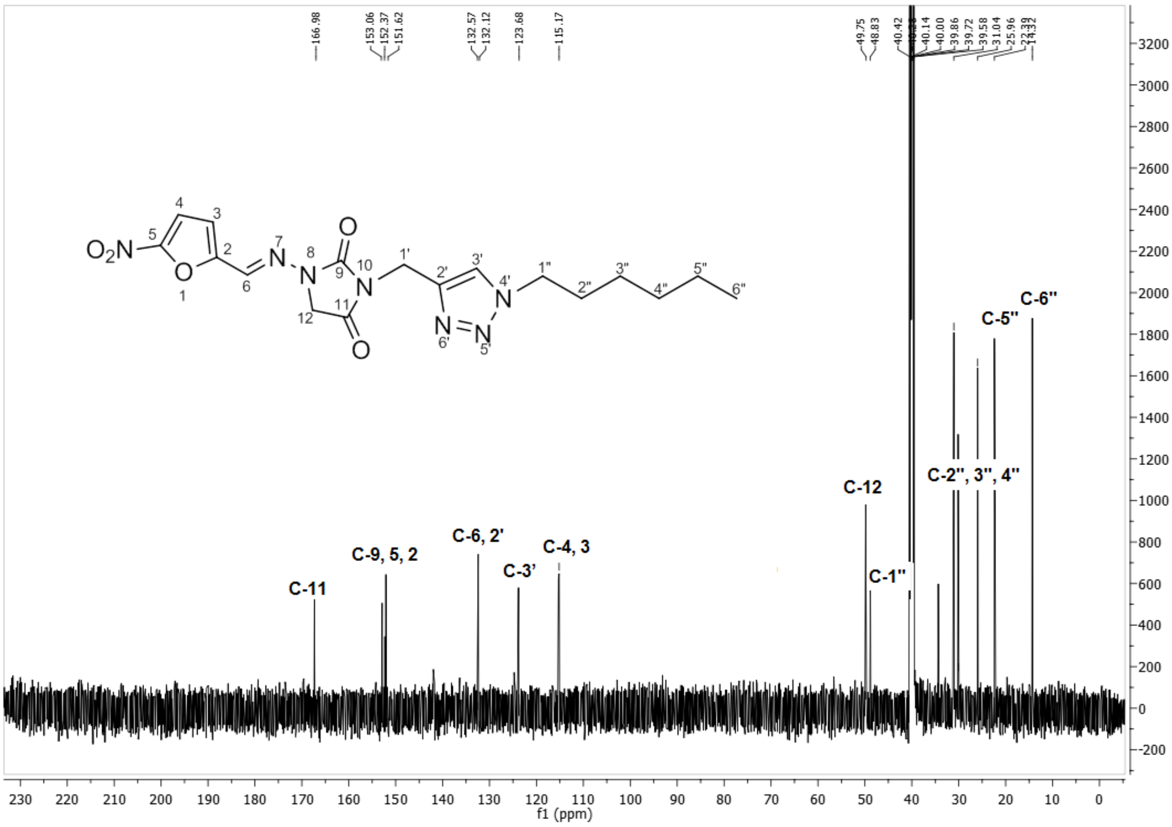

HRMS

Acquisition Parameter

Source Type	APCI	Ion Polarity	Positive	Set Nebulizer	1.6 Bar
Focus	Not active	Set Capillary	4500 V	Set Dry Heater	200 °C
Scan Begin	50 m/z	Set End Plate Offset	-500 V	Set Dry Gas	8.0 l/min
Scan End	1500 m/z	Set Collision Cell RF	100.0 Vpp	Set Divert Valve	Waste

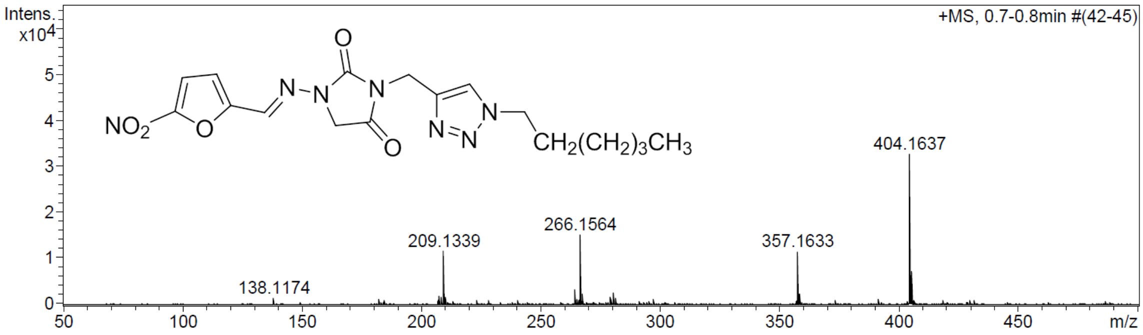

HPLC Purity

SHIMADZU LabSolutions

Analysis Report

<Sample Information>

Sample Name	: David NDa S3		
Sample ID	: David NDa S3		
Data Filename	: David NDa S3_006.lcd		
Method Filename	: PURITY non-polar.lcm		
Batch Filename	: David NDa.lcb		
Vial #	: 1-4	Sample Type	: Unknown
Injection Volume	: 1 uL		
Date Acquired	: 29/03/2022 09:40:12	Acquired by	: System Administrator
Date Processed	: 29/03/2022 09:53:13	Processed by	: System Administrator

<Chromatogram>

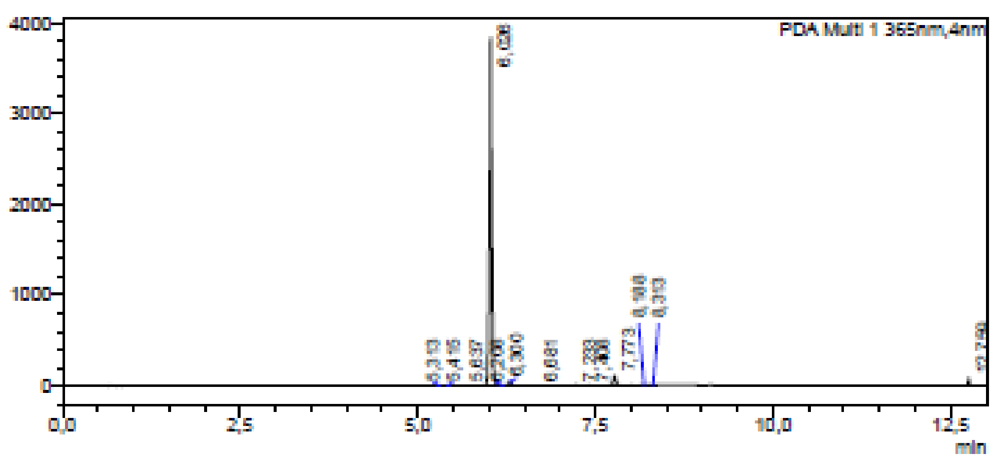

<Peak Table>

PDA Ch1 365nm

Peak#	Ret. Time	Area	Area%
1	5,313	11979	0,131
2	5,415	11859	0,129
3	5,637	24906	0,272
4	6,026	8479184	92,474
5	6,208	12029	0,131
6	6,300	147908	1,613
7	6,681	10477	0,114
8	7,233	13894	0,152
9	7,408	17880	0,195
10	7,773	312093	3,404
11	8,188	21525	0,235
12	8,313	30564	0,333
13	12,759	74928	0,817
Total		9169224	100,000

C:\LabSolutions\Data\Project1\PURITY\David NDa S3_006.lcd

(E)-1-{[(5-Nitrofuran-2-yl)methylene]amino}-10-[(4-heptyl-1H-1,2,3-triazol-1-yl)methyl]imidazolidine-9,11-dione, ***4***

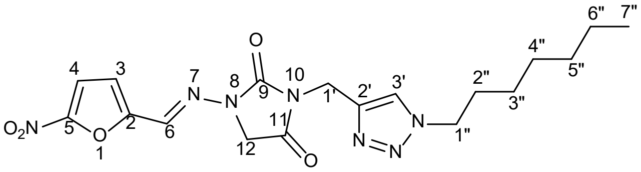

The reaction of **1** with 1-azidoheptane provided **4** as a yellow powder, 558 mg (74%), mp: 130.3-132.7 °C (EtOH/H_2O). IR (ATR) ν_{max}/cm^{-1}: 3138 (C-H, alkene), 2925 (C-H, alkane), 1784 (C=O), 1712 (C=O), 1344 (C-N). ^{1}H NMR (600 MHz, DMSO) δ (ppm): 8.08 (s, 1H, H-6), 7.89 (s, 1H, H-3'), 7.80 (d, *J* = 3.9 Hz, 1H, H-4), 7.18 (d, *J* = 3.9 Hz, 1H, H-3), 4.73 (s, 2H, H-1'), 4.48 (s, 2H, H-12), 4.31 (t, *J* = 7.2 Hz, 2H, H-1"), 1.79 – 1.75 (m, 2H, H-2"), 1.26 – 1.21 (m, 8H, H-3"…-6"), 0.98 - 0.85 (m, 3H, H-7"). ^{13}C NMR (151 MHz, DMSO) δ (ppm): 167.3 (C-11), 152.9 (C-9), 152.6 (C-5),152.1 (C-2), 132.4 (C-6, 2'), 123.8 (C-3'), 115.3 (C-4), 115.2 (C-3), 49.8 (C-1"), 48.8 (C-1'), 39.7 (C-2"), 39.6 (C-3"), 28.5 (C-4"), 26.3 (C-5"), 22.5 (C-6"), 14.3 (C-7"). Purity 90%. HRMS *m/z* $[M+H]^+$: 418.1811 (calcd. for $C_{18}H_{24}N_7O_5^+$ 418.1839).

IR

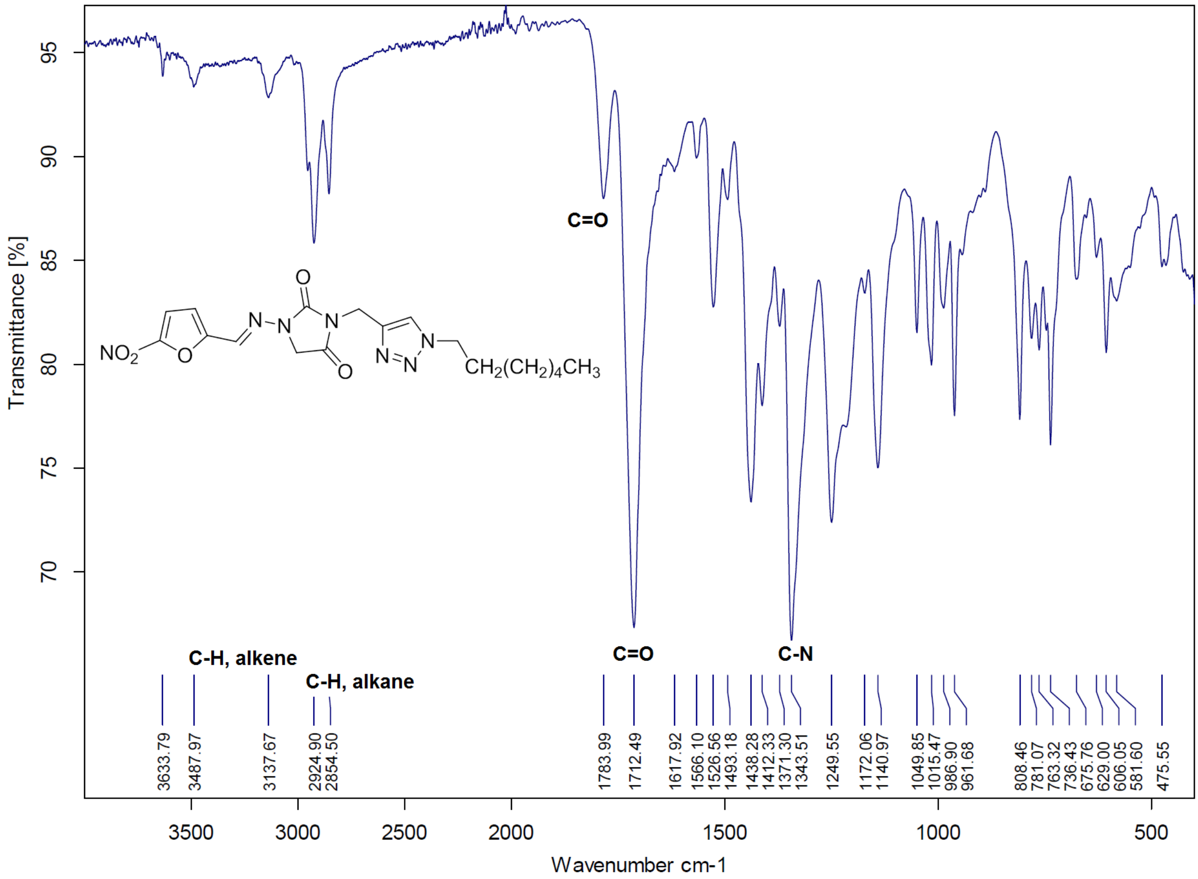

^{1}H NMR

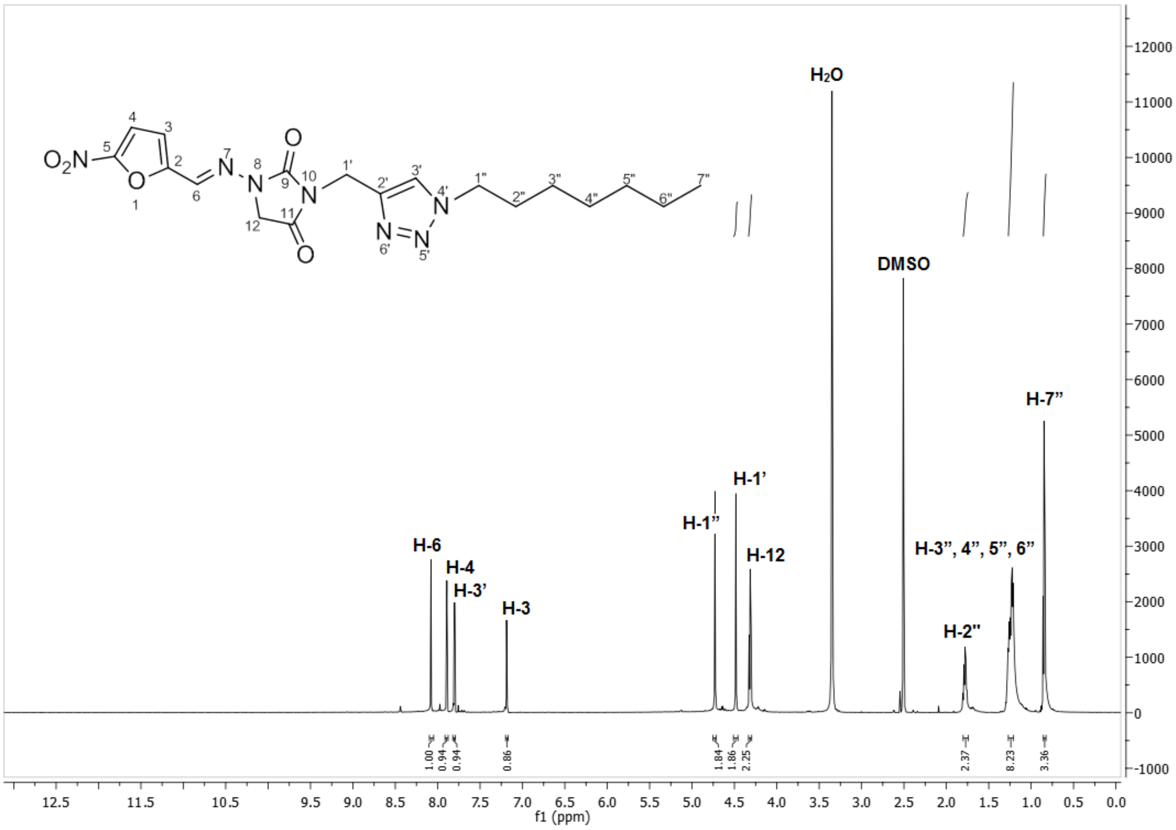

^{13}C NMR

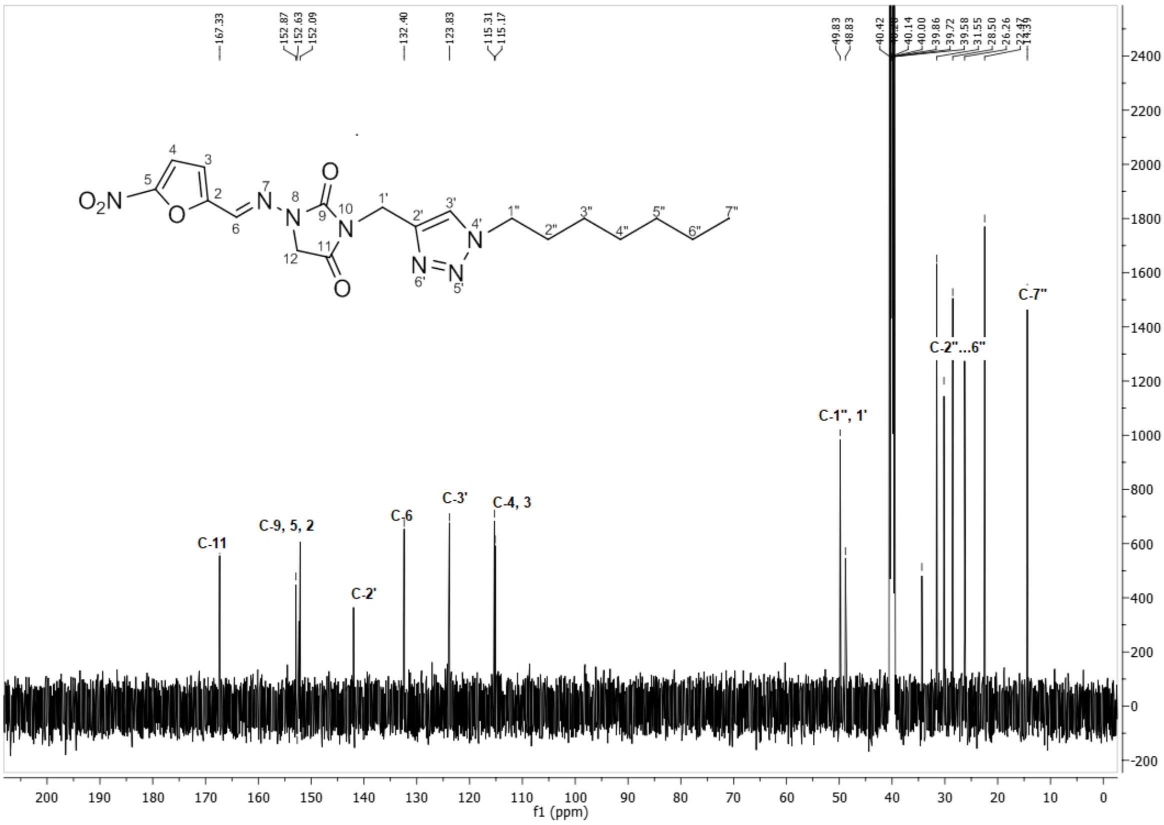

HRMS

Acquisition Parameter

Source Type	APCI	Ion Polarity	Positive	Set Nebulizer	1.6 Bar
Focus	Not active	Set Capillary	4500 V	Set Dry Heater	200 °C
Scan Begin	50 m/z	Set End Plate Offset	-500 V	Set Dry Gas	8.0 l/min
Scan End	1500 m/z	Set Collision Cell RF	100.0 Vpp	Set Divert Valve	Waste

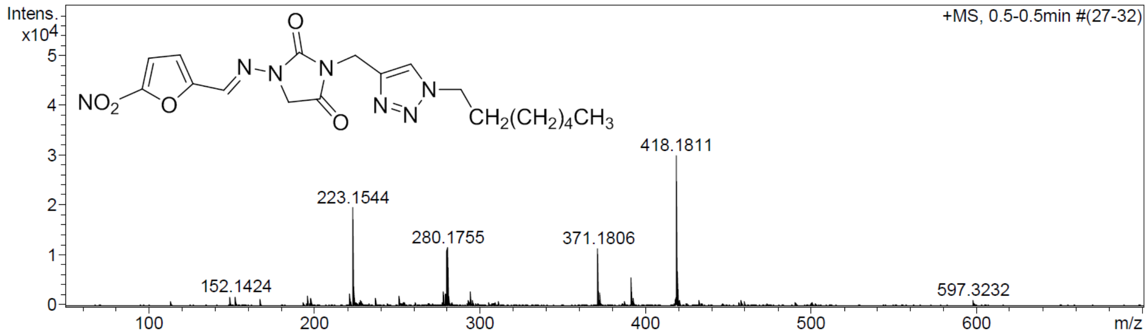

HPLC Purity

LabSolutions Analysis Report

<Sample Information>

Sample Name	: David NDa S4		
Sample ID	: David NDa S4		
Data Filename	: David NDa S4_007.lcd		
Method Filename	: PURITY non-polar.lcm		
Batch Filename	: David NDa.lcb		
Vial #	: 1-5	Sample Type	: Unknown
Injection Volume	: 1 uL		
Date Acquired	: 29/03/2022 09:53:33	Acquired by	: System Administrator
Date Processed	: 29/03/2022 10:06:34	Processed by	: System Administrator

<Chromatogram>

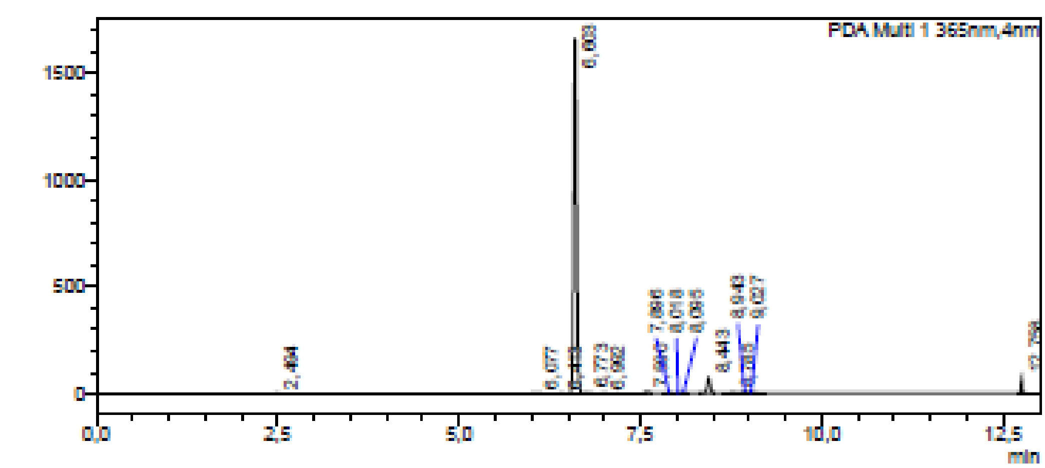

<Peak Table>

PDA Ch1 365nm

Peak#	Ret. Time	Area	Area%
1	2,494	12558	0,239
2	6,077	26249	0,499
3	6,413	10278	0,195
4	6,603	4735329	90,023
5	6,773	22230	0,423
6	6,992	10117	0,192
7	7,590	20535	0,390
8	7,896	17477	0,332
9	8,018	14048	0,267
10	8,095	19681	0,374
11	8,443	220345	4,189
12	8,785	24099	0,458
13	8,943	31781	0,604
14	9,027	18262	0,347
15	12,758	77171	1,467
Total		5260159	100,000

C:\LabSolutions\Data\Project1\PURITY\David NDa S4_007.lcd

(E)-1-{[(5-Nitrofuran-2-yl)methylene]amino}-10-[(4-octyl-1H-1,2,3-triazol-1-yl)methyl]imidazolidine-9,11-dione, ***5***

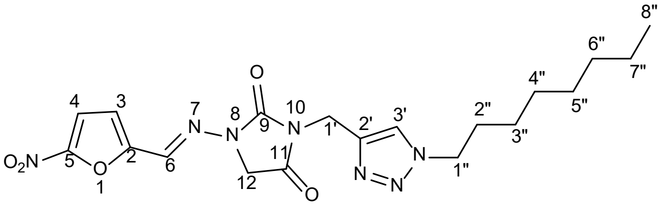

1 and 1-azidooctane yielded **5** as a yellow powder, 598 mg (77%), mp: 127.9-130.3 °C (EtOH/H_2O). IR (ATR) ν_{max}/cm^{-1}: 3137 (C-H, alkene), 2924 (C-H, alkane), 1785 (C=O), 1718 (C=O), 1343 (C-N). ^{1}H NMR (600 MHz, DMSO) δ (ppm): 8.08 (s, 1H, H-6), 7.89 (s, 1H, H-3'), 7.80 (d, *J* = 3.9 Hz, 1H, H-4), 7.18 (d, *J* = 3.9 Hz, 1H, H-3), 4.73 (s, 2H, H-1'), 4.48 (s, 2H, H-12), 4.31 (t, *J* = 7.2 Hz, 2H, H-1"), 1.79 – 1.75 (m, 2H, H- 2"), 1.26 – 1.21 (m, 10H, H-3"…-7"), 0.92 -0.84 (m, 3H, H-8"). ^{13}C NMR (151 MHz, DMSO) δ (ppm): ^{13}C NMR (151 MHz, DMSO) δ (ppm): 167.3 (C-11), 152.9 (C-9), 152.3 (C-5),152.1 (C-2), 132.4 (C-6, 2'), 123.8 (C-3'), 115.3 (C-4), 115.2 (C-3), 49.8 (C-1"), 48.8 (C-1'), 39.7 (C-2"), 39.6 (C-3"), 28.5 (C-4"), 28.8 (C-5"), 22.5 (C-7"), 14.4 (C-8"). Purity 89%. HRMS *m/z* $[M+H]^{+}$: 432.1966 (calcd. for $C_{19}H_{26}N_7O_5^{+}$ 432.1995).

IR

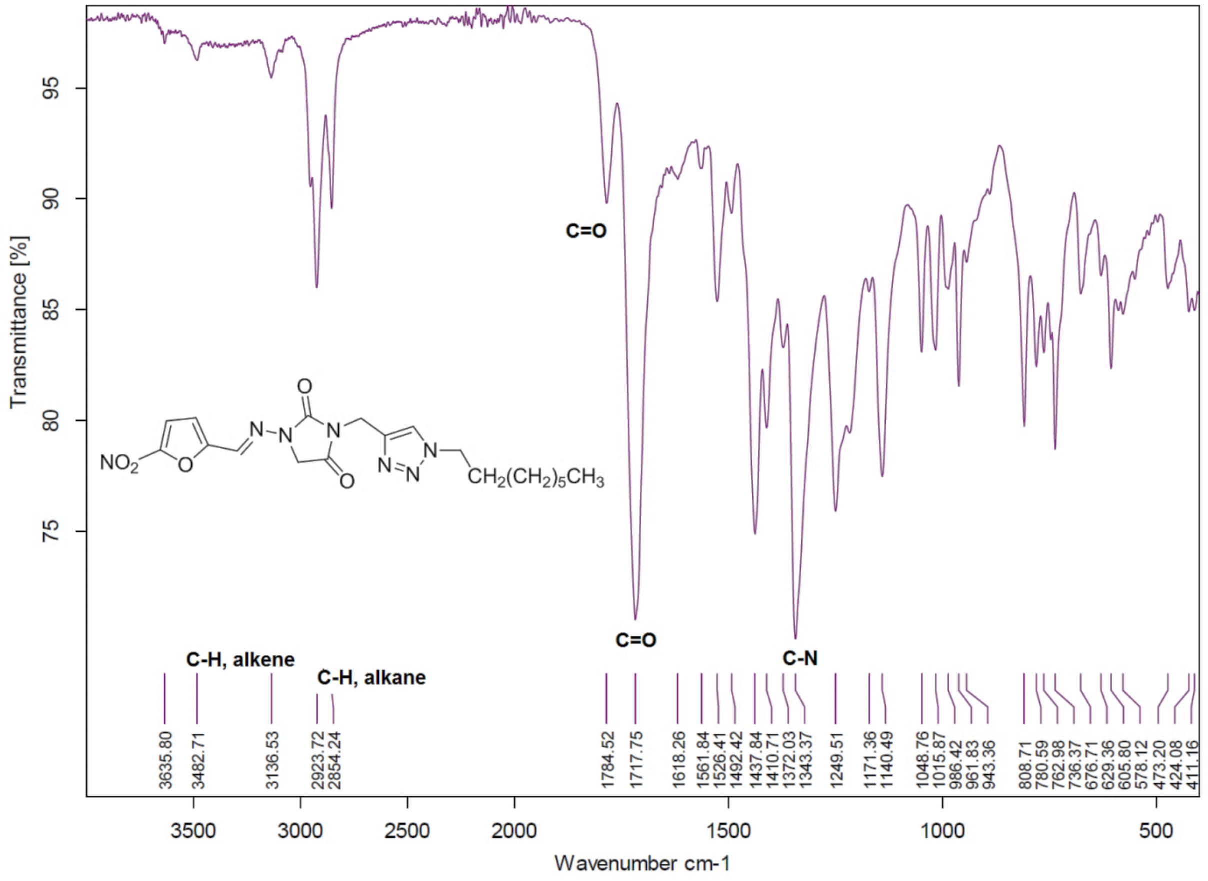

^{1}H-NMR

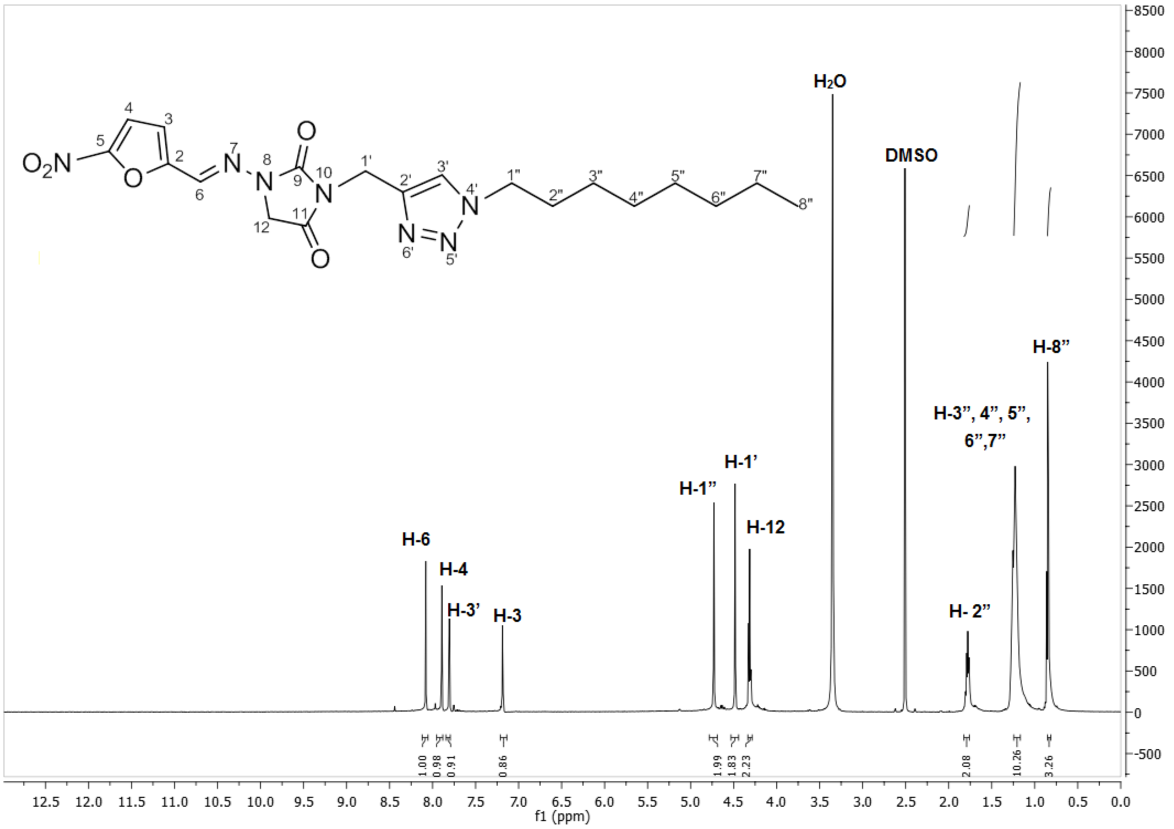

^{13}C-NMR

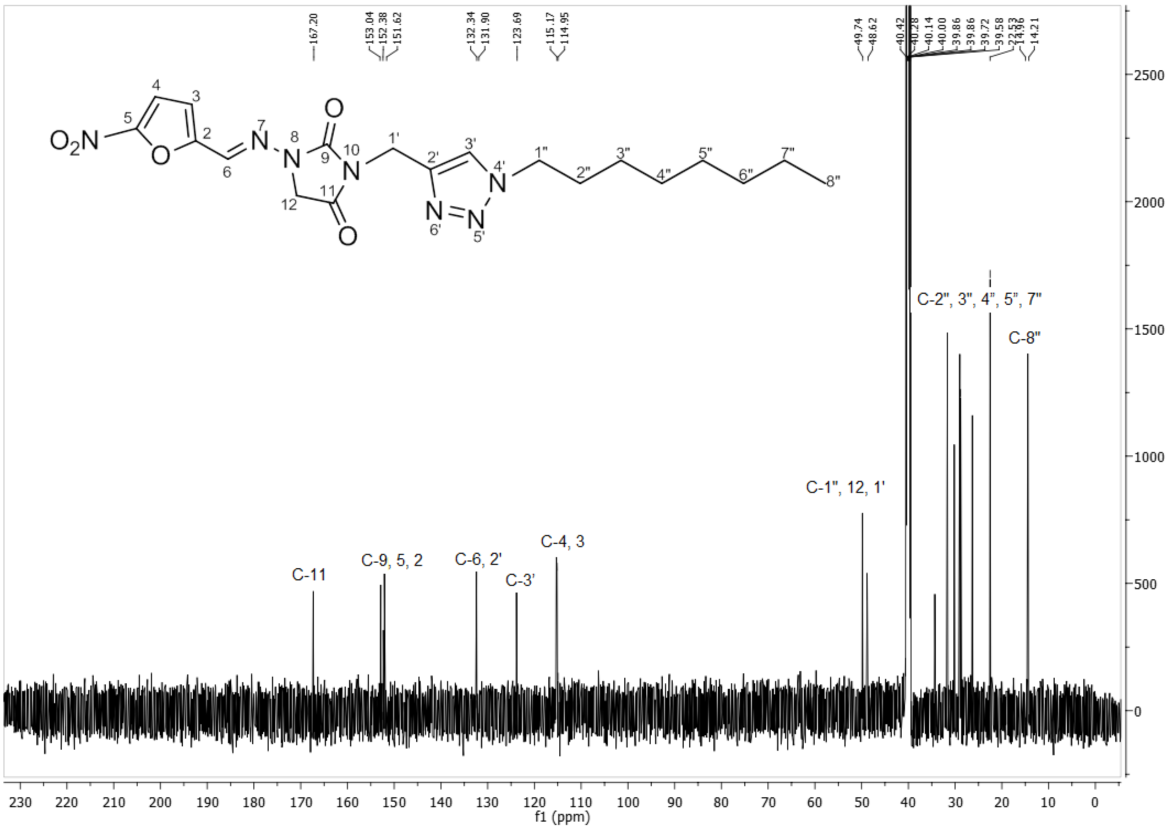

HRMS

Acquisition Parameter

Source Type	APCI	Ion Polarity	Positive	Set Nebulizer	1.6 Bar
Focus	Not active	Set Capillary	4500 V	Set Dry Heater	200 °C
Scan Begin	50 m/z	Set End Plate Offset	-500 V	Set Dry Gas	8.0 l/min
Scan End	1500 m/z	Set Collision Cell RF	100.0 Vpp	Set Divert Valve	Waste

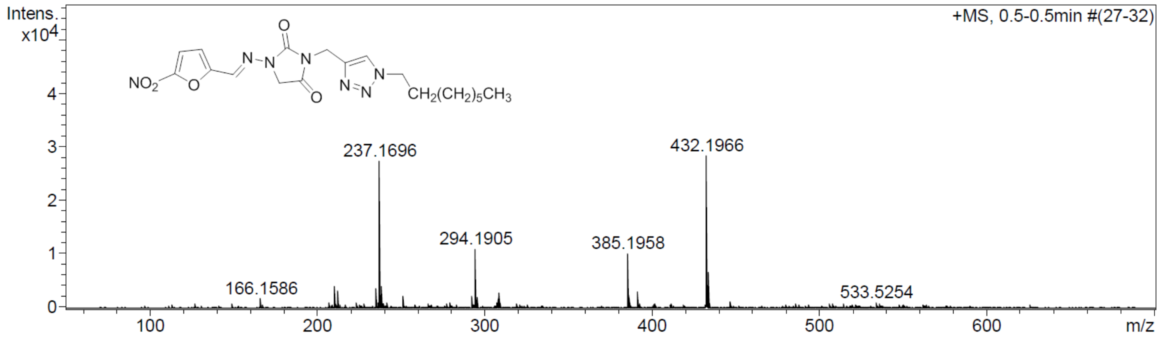

HPLC Purity

SHIMADZU LabSolutions

Analysis Report

<Sample Information>

Sample Name	: David NDa S5		
Sample ID	: David NDa S5		
Data Filename	: David NDa S5_008.lcd		
Method Filename	: PURITY non-polar.lcm		
Batch Filename	: David NDa.lcb		
Vial #	: 1-6	Sample Type	: Unknown
Injection Volume	: 1 uL		
Date Acquired	: 29/03/2022 10:06:54	Acquired by	: System Administrator
Date Processed	: 29/03/2022 10:19:55	Processed by	: System Administrator

<Chromatogram>

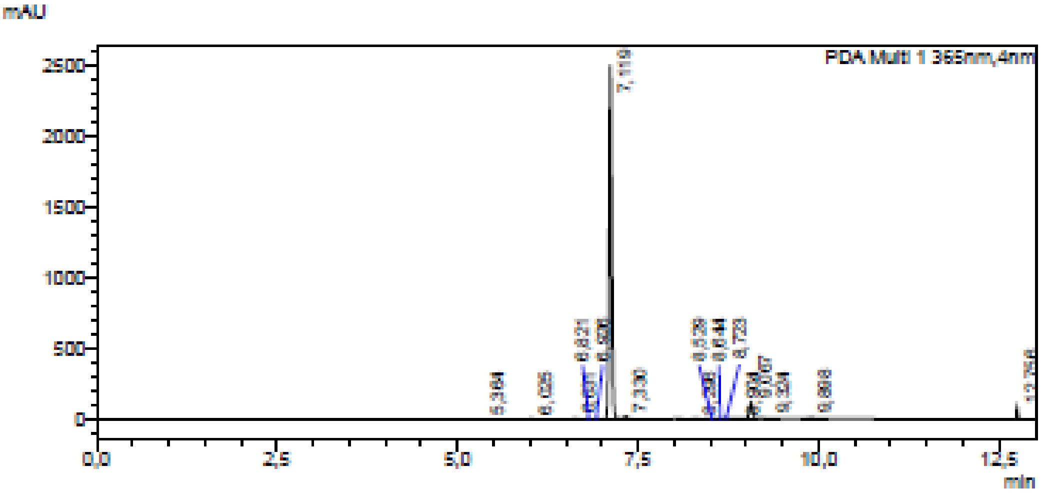

<Peak Table>

PDA Ch1 365nm

Peak#	Ret. Time	Area	Area%
1	5,364	10420	0,146
2	6,025	17661	0,247
3	6,631	26705	0,374
4	6,821	20203	0,283
5	6,926	21285	0,298
6	7,119	6345631	88,799
7	7,330	76662	1,073
8	8,296	14213	0,199
9	8,529	24826	0,347
10	8,644	17545	0,246
11	8,723	32433	0,454
12	8,904	20019	0,280
13	9,067	383255	5,363
14	9,324	15642	0,219
15	9,898	44911	0,628
16	12,756	74679	1,045
Total		7146089	100,000

C:\LabSolutions\Data\Project1\PURITY\David NDa S5_008.lcd

(E)-1-{[(5-Nitrofuran-2-yl)methylene]amino}-10-[(4-nonyl-1H-1,2,3-triazol-1-yl)methyl]imidazolidine-9,11-dione, ***6***

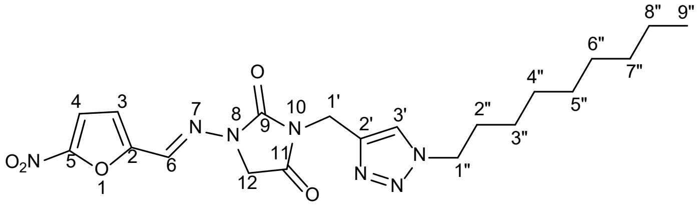

The reaction of **1** with1-azidononane provided **6** as a dark orange powder, 467 mg (58%), mp: 123.8-126.1 °C (EtOH/H_2O). IR (ATR) ν_{max}/cm^{-1}: 3142 (C-H, alkene), 2920 (C-H, alkane), 1783 (C=O), 1713 (C=O), 1345 (C-N). ^{1}H NMR (600 MHz, DMSO) δ (ppm): 8.08 (s, 1H, H-6), 7.89 (s, 1H, H-3'), 7.80 (d, *J* = 3.9 Hz, 1H, H-4), 7.18 (d, *J* = 3.9 Hz, 1H, H-3), 4.73 (s, 2H, H-1'), 4.48 (s, 2H, H-12), 4.31 (t, *J* = 7.2 Hz, 2H, H-1"), 1.79 – 1.75 (m, 2H, H- 2"), 1.26 – 1.21 (m, 12H, H-3"…-8"), 0.91 - 0.85 (m, 3H, H-9"). ^{13}C NMR (151 MHz, DMSO) δ (ppm): 167.3 (C-11), 152.9 (C-9), 152.3 (C-5), 152.1 (C-2), 132.4 (C-6, 2'), 123.8 (C-3'), 115.4 (C-4), 115.2 (C-3), 49.8 (C-1"), 48.6 (C-1'), 31.7 (C-7"), 29.3 (C-5"), 29.1 (C-4"), 28.9 (C-6), 26.3 (C-3"), 22.6 (C-8"), 14.4 (C-9"). Purity 92%. HRMS *m/z* $[M+H]^+$: 446.2131 (calcd. for $C_{20}H_{28}N_7O_5^+$ 446.2152).

IR

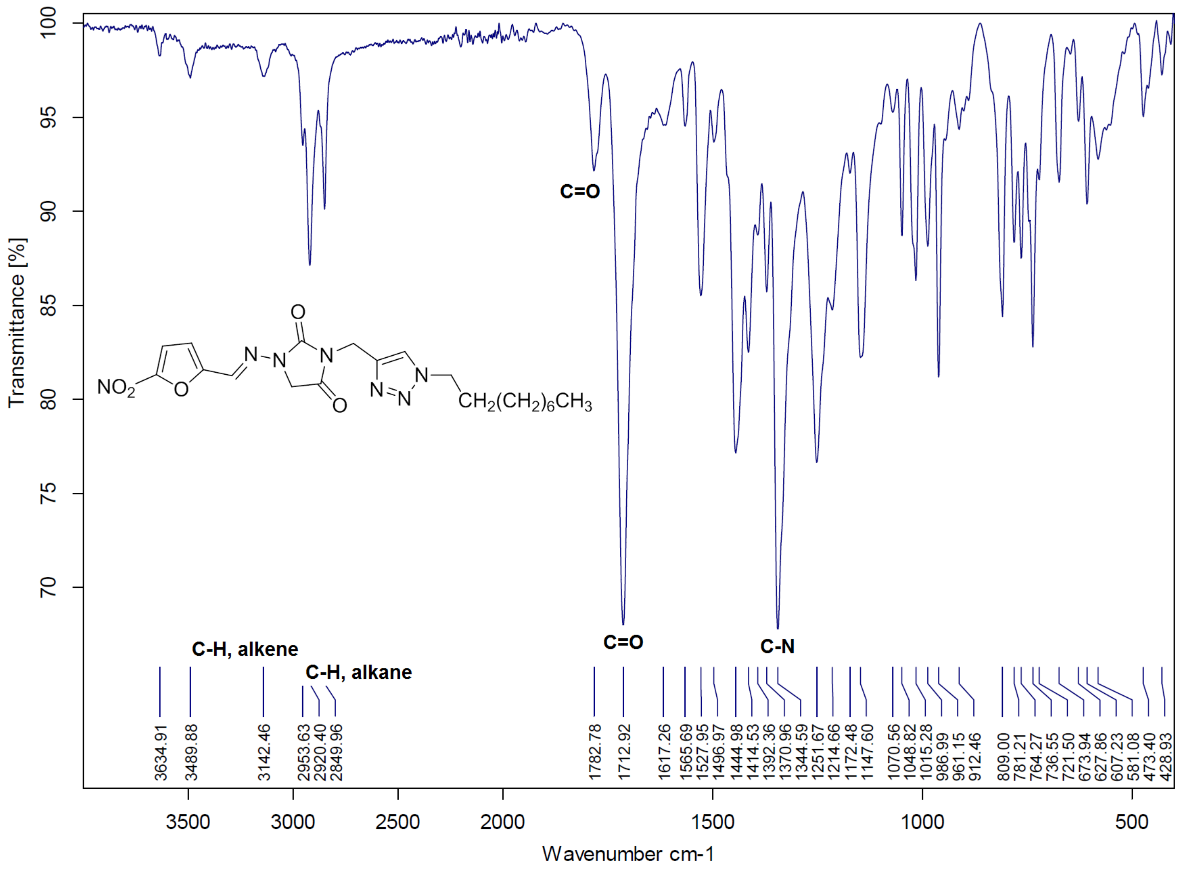

^{1}H-NMR

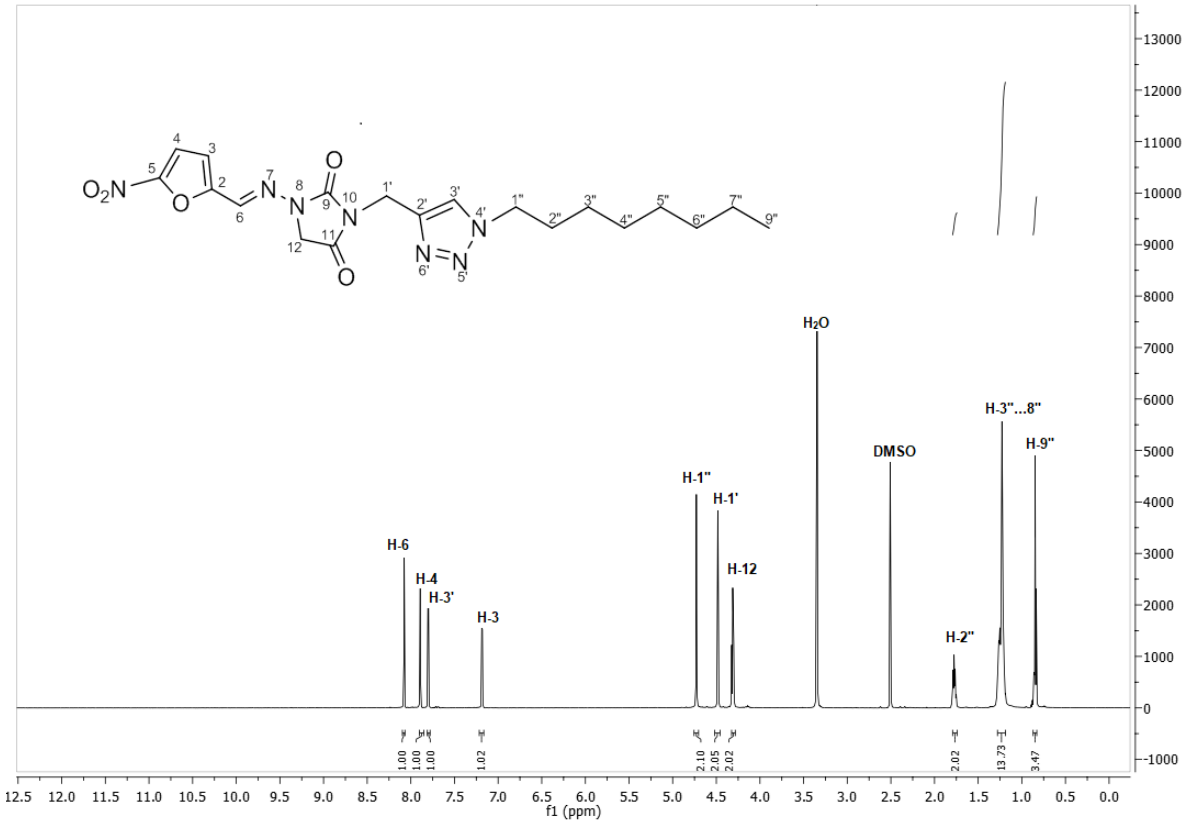

^{13}C-NMR

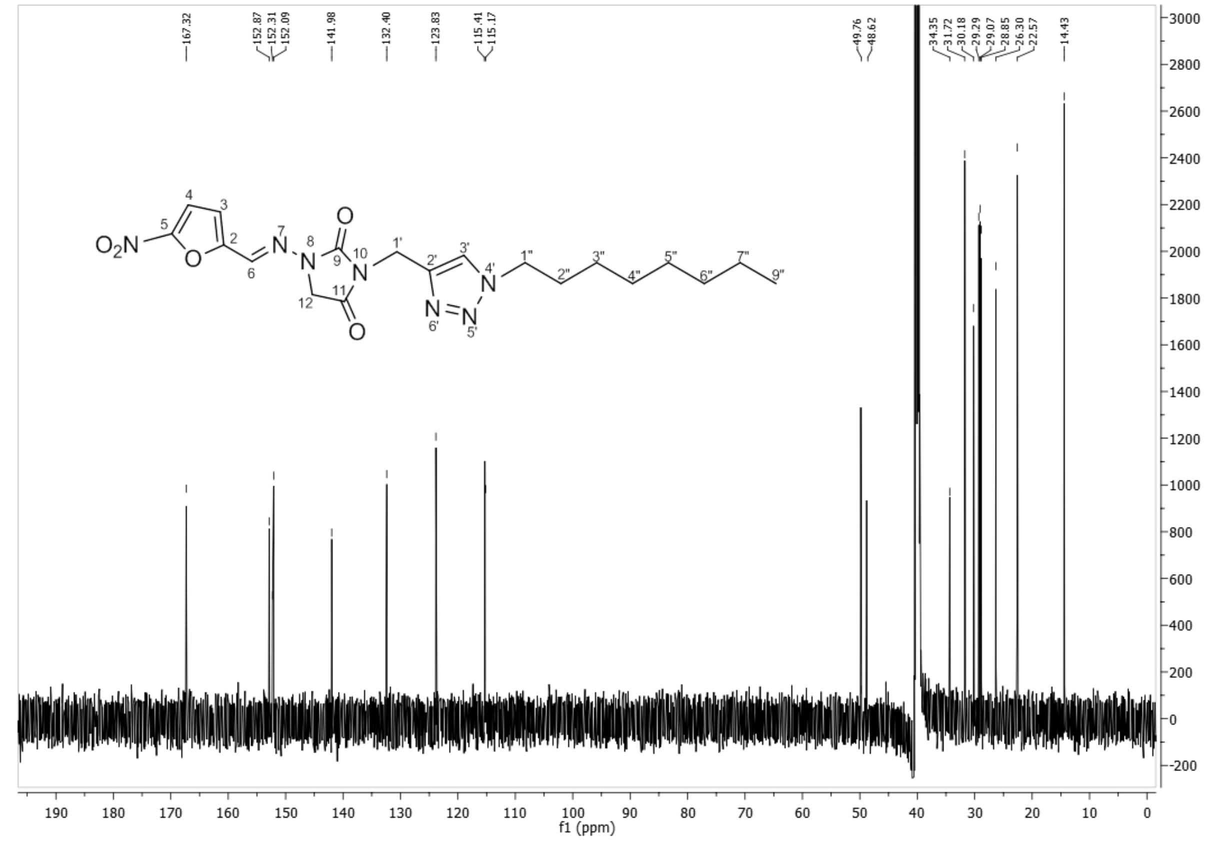

HRMS

Acquisition Parameter

Source Type	APCI	Ion Polarity	Positive	Set Nebulizer	1.6 Bar
Focus	Not active	Set Capillary	4500 V	Set Dry Heater	200 °C
Scan Begin	50 m/z	Set End Plate Offset	-500 V	Set Dry Gas	8.0 l/min
Scan End	1500 m/z	Set Collision Cell RF	100.0 Vpp	Set Divert Valve	Waste

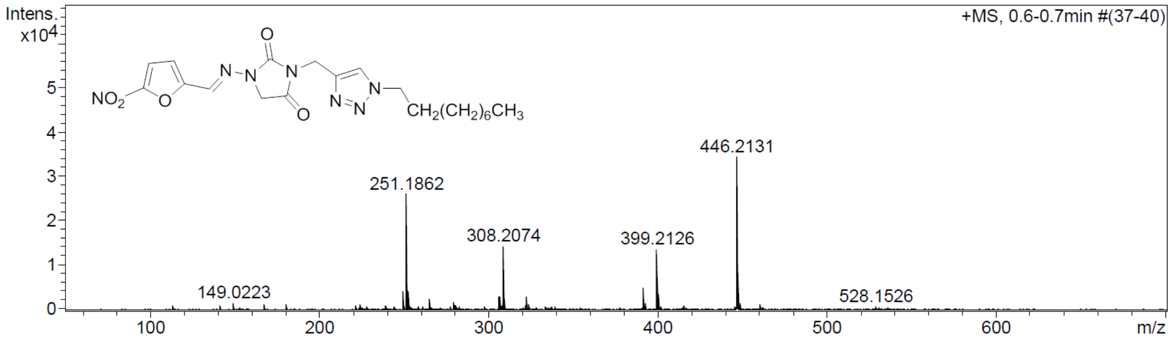

HPLC Purity

SHIMADZU LabSolutions

Analysis Report

<Sample Information>

Sample Name	: David NDa S6		
Sample ID	: David NDa S6		
Data Filename	: David NDa S6_009.lcd		
Method Filename	: PURITY non-polar.lcm		
Batch Filename	: David NDa.lcb		
Vial #	: 1-7	Sample Type	: Unknown
Injection Volume	: 1 uL		
Date Acquired	: 29/03/2022 10:20:14	Acquired by	: System Administrator
Date Processed	: 29/03/2022 10:33:16	Processed by	: System Administrator

<Chromatogram>

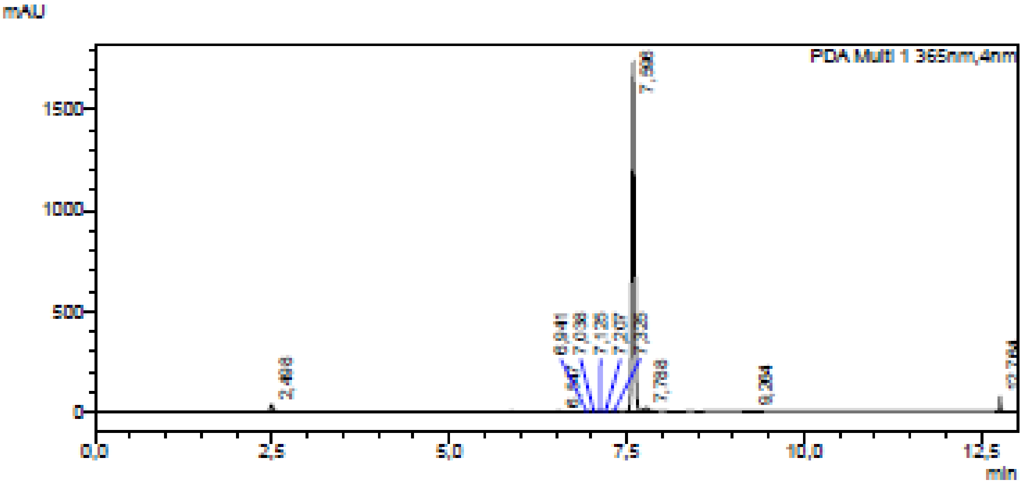

<Peak Table>

PDA Ch1 365nm

Peak#	Ret. Time	Area	Area%
1	2,498	105984	2,062
2	6,547	11491	0,224
3	6,941	15686	0,305
4	7,038	18908	0,368
5	7,125	19400	0,377
6	7,207	10018	0,195
7	7,325	18800	0,366
8	7,596	4740887	92,239
9	7,788	106104	2,064
10	9,264	15417	0,300
11	12,764	77075	1,500
Total		5139771	100,000

C:\LabSolutions\Data\Project1\PURITY\David NDa S6_009.lcd

(E)-1-{[(5-Nitrofuran-2-yl)methylene]amino}-10-[(4-decyl-1H-1,2,3-triazol-1-yl)methyl]imidazolidine-9,11-dione, ***7***

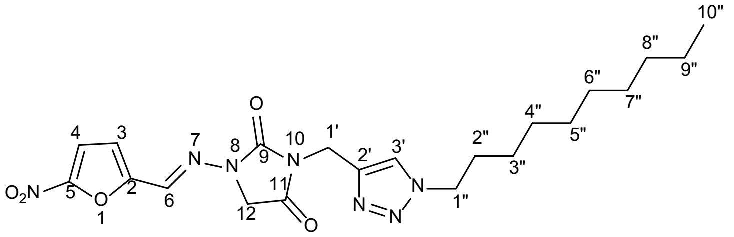

The reaction of **1** with 1-azidodecane afforded **7** as a fine orange powder, 638 mg (77%), mp: 148.0-148-8 °C (EtOH/H_2O). IR (ATR) ν_{max}/cm^{-1}: 2920 (C-H, alkane), 1785 (C=O), 1713 (C=O), 1344 (C-N). ^{1}H NMR (600 MHz, DMSO) δ (ppm): 8.08 (s, 1H, H-6), 7.89 (s, 1H, H-3'), 7.80 (d, *J* = 4.2 Hz, 1H, H-4), 7.19 (d, *J* = 4.2 Hz, 1H, H-3), 4.73 (s, 2H, H-1'), 4.48 (s, 2H, H-12), 4.31 (t, *J* = 7.1 Hz, 2H, H-1"), 1.87 – 1.74 (m, 2H, H-2"), 1.38 – 1.24 (m, H-3"…-9"), 0.98 – 0.84 (m, *J* = 7.1 Hz, 3H, H-10"). ^{13}C NMR (151 MHz, DMSO) δ (ppm): 167.3 (C-11), 152.9 (C-9), 152.1 (C-5), 151.9 (C-2), 132.4 (C-6), 132.1 (C-2'), 123.9 (C-3'), 115.3 (C-4), 115.2 (C-3), 49.5 (C-1"), 48.6 (C-1'), 31.5 (C-7"), 30.3 (C-5"), 26.3 (C-3"), 22.5 (C-9"), 14.5 (C-10"). Purity 93%. HRMS *m/z* [M+H]$^+$: 460.2295 (calcd. for $C_{21}H_{30}N_7O_5^+$ 460.2308).

IR

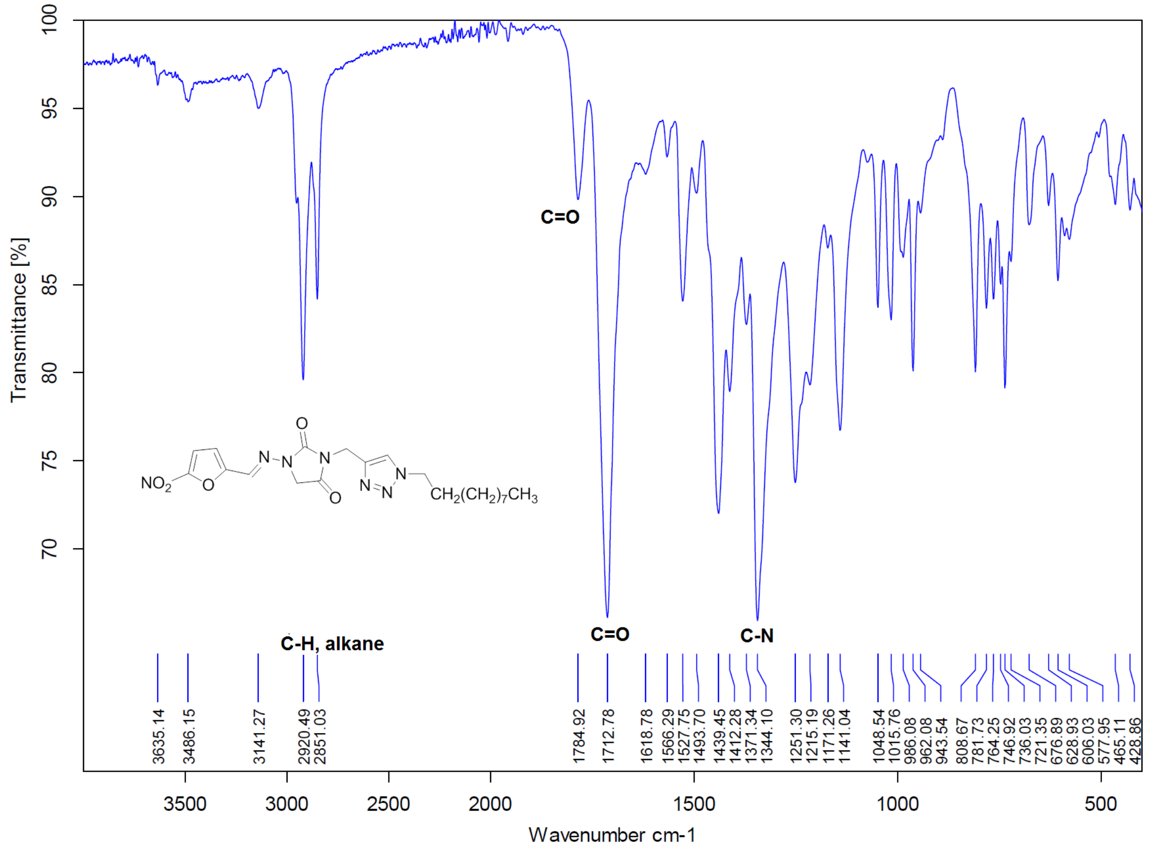

^{1}**H-NMR**

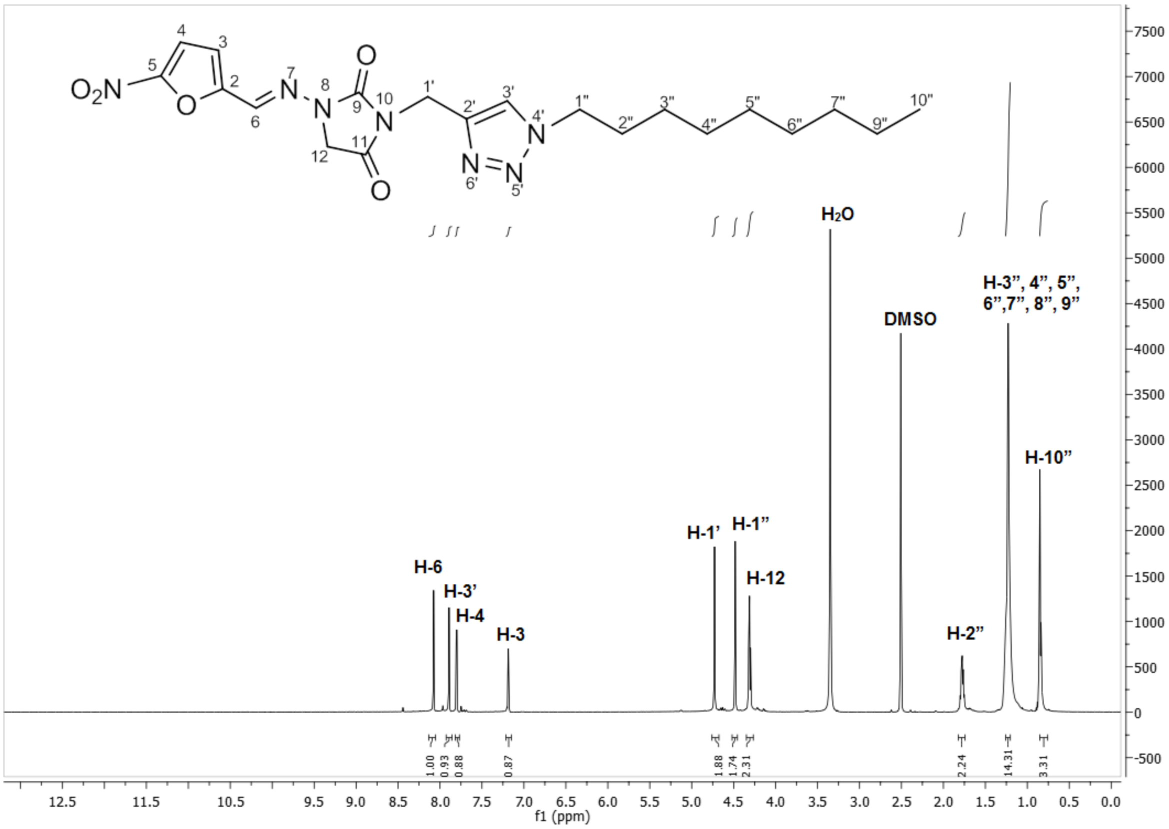

^{13}**C-NMR**

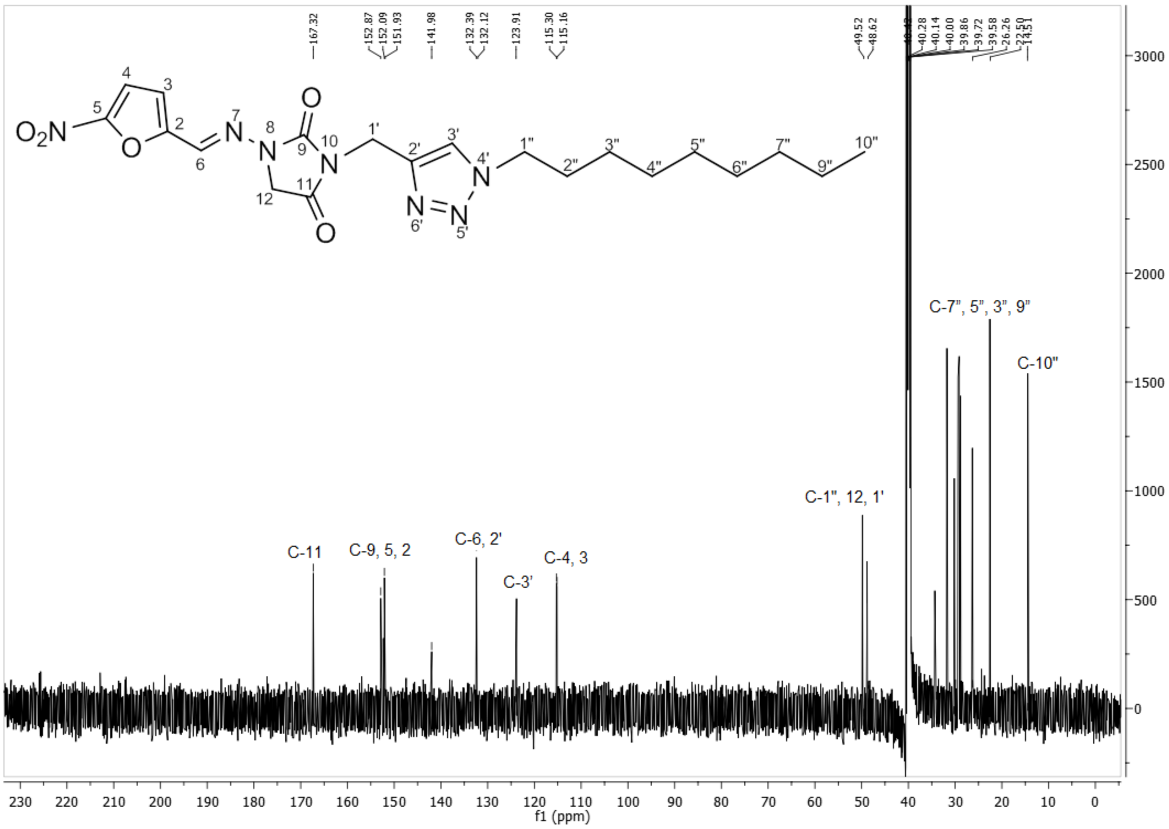

HRMS

Acquisition Parameter

Source Type	APCI	Ion Polarity	Positive	Set Nebulizer	1.6 Bar
Focus	Not active	Set Capillary	4500 V	Set Dry Heater	200 °C
Scan Begin	50 m/z	Set End Plate Offset	-500 V	Set Dry Gas	8.0 l/min
Scan End	1500 m/z	Set Collision Cell RF	100.0 Vpp	Set Divert Valve	Waste

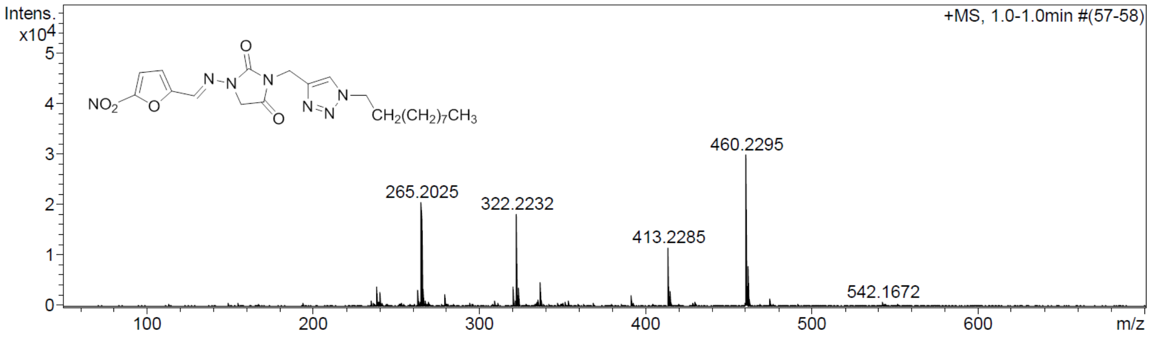

HPLC Purity

SHIMADZU LabSolutions **Analysis Report**

<Sample Information>

Sample Name	: David NDa S7		
Sample ID	: David NDa S7		
Data Filename	: David NDa S7_010.lcd		
Method Filename	: PURITY non-polar.lcm		
Batch Filename	: David NDa.lcb		
Vial #	: 1-8	Sample Type	: Unknown
Injection Volume	: 1 uL		
Date Acquired	: 29/03/2022 10:33:36	Acquired by	: System Administrator
Date Processed	: 31/03/2022 08:33:43	Processed by	: System Administrator

<Chromatogram>

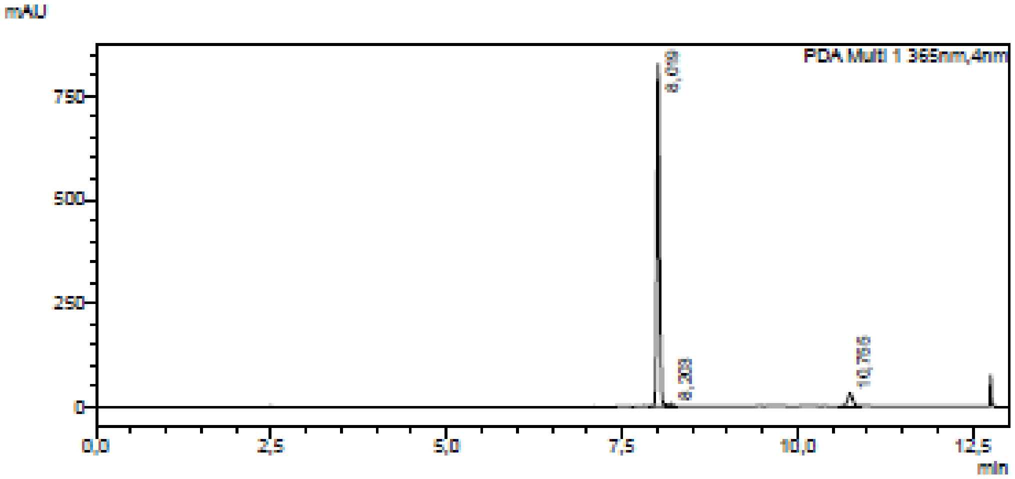

<Peak Table>

PDA Ch1 365nm

Peak#	Ret. Time	Area	Area%
1	8,019	2323792	92,502
2	8,203	27372	1,090
3	10,755	160980	6,408
Total		2512143	100,000

C:\LabSolutions\Data\Project1\PURITY\David NDa S7_010.lcd

(E)-1-{[(5-Nitrofuran-2-yl)methylene]amino}-10-[(4-undecyl-1H-1,2,3-triazol-1-yl)methyl]imidazolidine-9,11-dione, ***8***

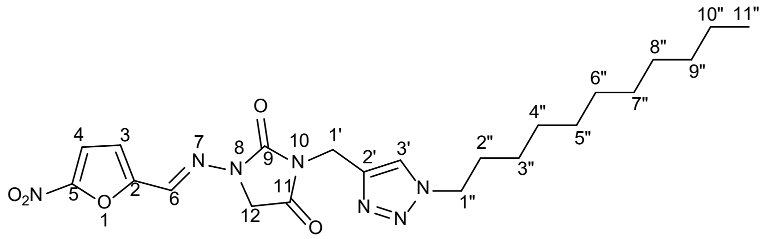

The reaction of **1** with 1-azidoundecane yielded **8** as a dark yellow powder, 604 mg (70%), mp: 163.3-163.9 °C (EtOH/H_2O). IR (ATR) ν_{max}/cm^{-1}: 3145 (C-H, alkene), 2919 (C-H, alkane), 2849 (C-H), 1781 (C=O), 1715 (C=O), 1345 (C-N). ^{1}H NMR (600 MHz, DMSO) δ (ppm): 8.08 (s, 1H, H-6), 7.89 (s, 1H, H-3'), 7.81 (d, *J* = 4.1 Hz, 1H, H-4), 7.19 (d, *J* = 4.1 Hz, 1H, H-3), 4.71 (s, 2H, H-1'), 4.48 (s, 2H, H-12), 4.31 (t, *J* = 7.1 Hz, 2H, H-1"), 1.78 (dt, *J* = 14.4, 7.2 Hz, 2H, H-2"), 1.36 - 1.02 (m, 16H, H-3"…-10"), 0.99 - 0.84 (m, 3H, H-11"). ^{13}C NMR (151 MHz, DMSO) δ (ppm): 167.2 (C-11), 152.8 (C-9), 151.9 (C-5), 132.3 (C-6, 2'), 123.7 (C-3'), 115.2 (C-4), 114.7 (C-3), 49.7 (C-1"), 48.6 (C-1', 12), 29.50 (C-7"), 29.4 (C-2"), 29.2 (C-8"), 26.3 (C-3"), 22.6 (C-10"), 14.4 (C-11"). Purity: 77%. HRMS *m/z* [M+H]$^+$: 474.2406 (calcd. for $C_{22}H_{32}N_7O_5^+$ 474.2465).

IR

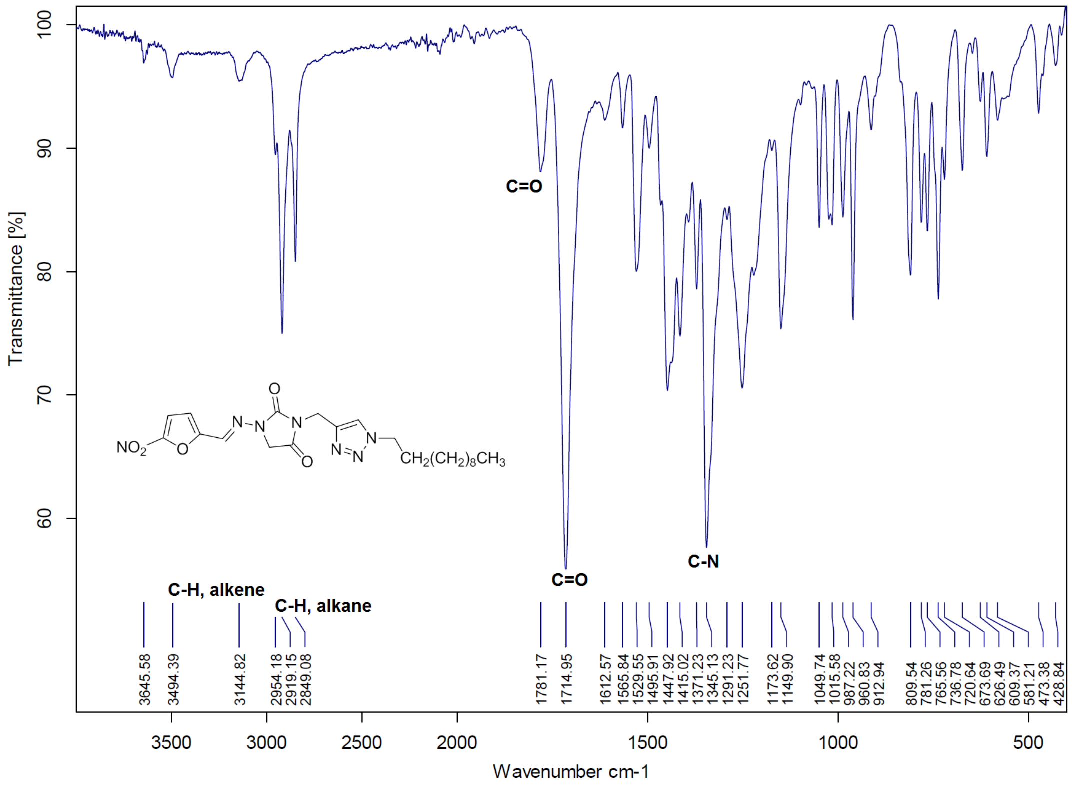

^{1}H-NMR

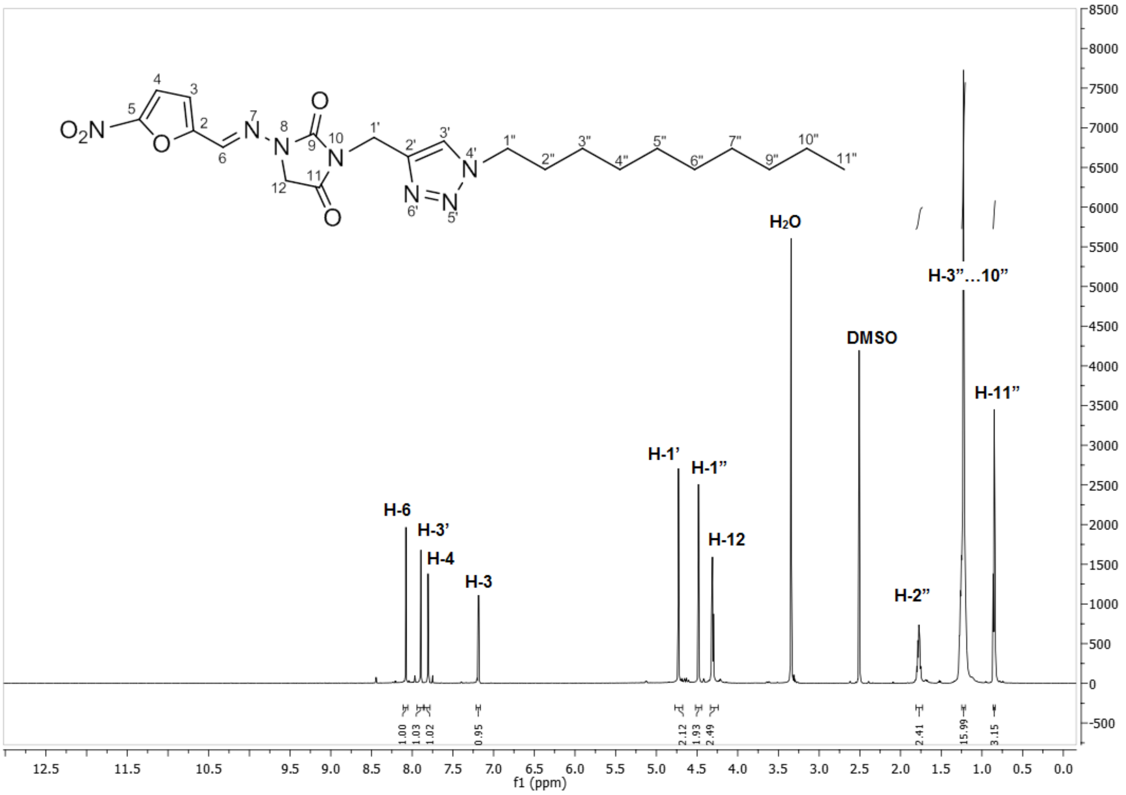

^{13}C-NMR

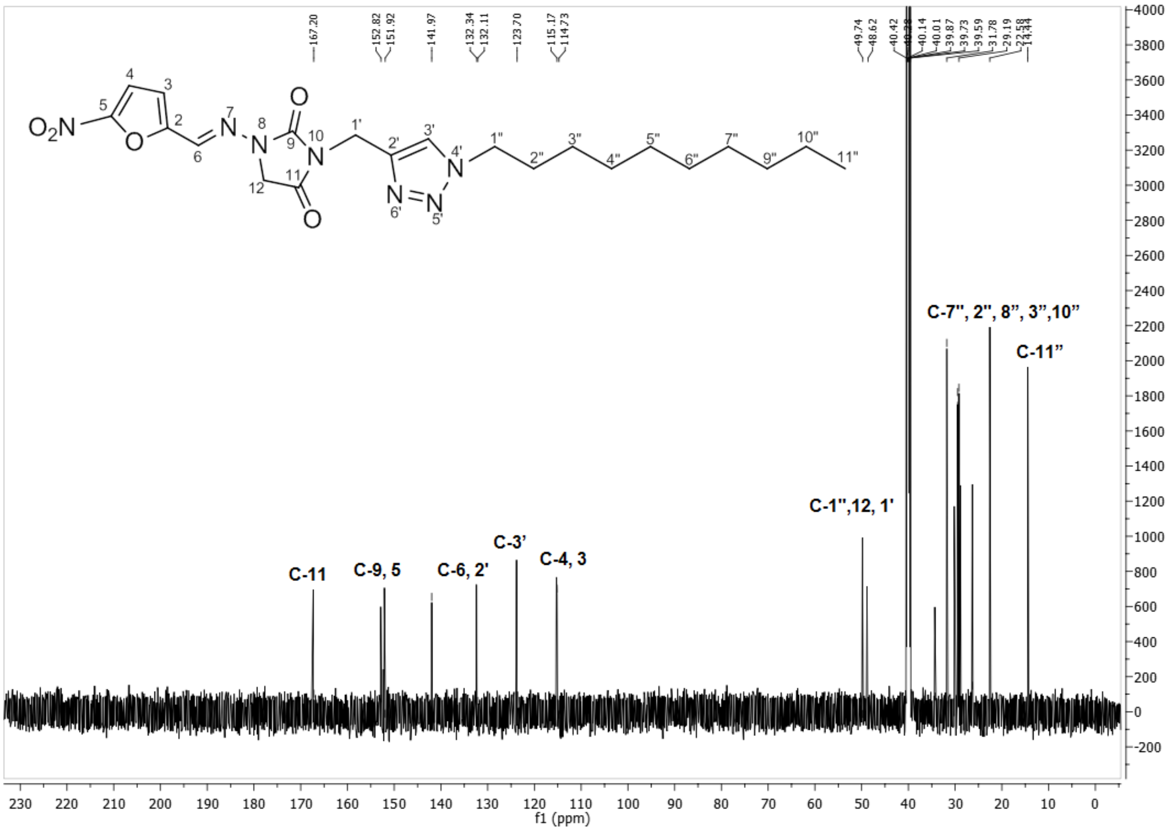

HRMS

Acquisition Parameter

Source Type	APCI	Ion Polarity	Positive	Set Nebulizer	1.6 Bar
Focus	Not active	Set Capillary	4500 V	Set Dry Heater	200 °C
Scan Begin	50 m/z	Set End Plate Offset	-500 V	Set Dry Gas	8.0 l/min
Scan End	1500 m/z	Set Collision Cell RF	100.0 Vpp	Set Divert Valve	Waste

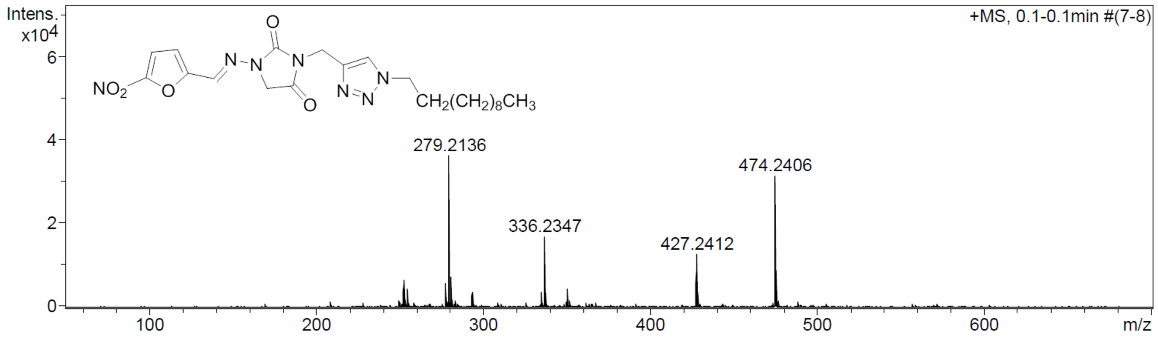

HPLC Purity

LabSolutions Analysis Report

<Sample Information>

Sample Name	: David NDa S8		
Sample ID	: David NDa S8		
Data Filename	: David NDa S8_011.lcd		
Method Filename	: PURITY non-polar.lcm		
Batch Filename	: David NDa.lcb		
Vial #	: 1-9	Sample Type	: Unknown
Injection Volume	: 1 uL		
Date Acquired	: 29/03/2022 10:46:58	Acquired by	: System Administrator
Date Processed	: 29/03/2022 11:00:00	Processed by	: System Administrator

<Chromatogram>

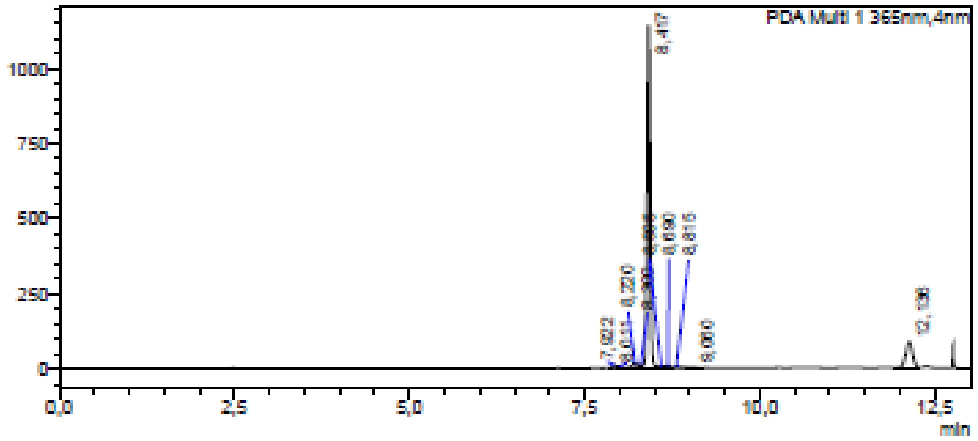

<Peak Table>

PDA Ch1 365nm

Peak#	Ret. Time	Area	Area%
1	7,922	43721	1,035
2	8,011	30933	0,732
3	8,220	92688	2,194
4	8,300	54101	1,280
5	8,417	3249534	76,909
6	8,595	39558	0,936
7	8,690	40501	0,959
8	8,815	43639	1,033
9	9,060	26119	0,618
10	12,136	604348	14,304
Total		4225140	100,000

C:\LabSolutions\Data\Project1\PURITY\David NDa S8_011.lcd

(E)-1-{[(5-Nitrofuran-2-yl)methylene]amino}-10-[(4-dodecyl-1H-1,2,3-triazol-1-yl)methyl]imidazolidine-9,11-dione, ***9***

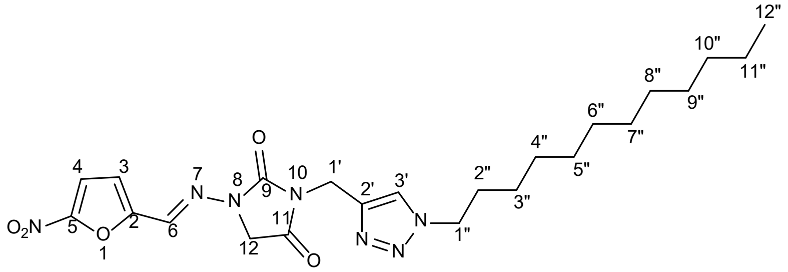

The reaction of **1** with 1-azidododecane resulted in **9** as an orange yellow powder, 566 mg (64%), mp: 163.9-165.8 °C (EtOH/H_2O). IR (ATR) ν_{max}/cm^{-1}: 3145 (C-H, alkene), 2919 (C-H, alkane), 2849 (C-H, alkane), 1782 (C=O), 1715 (C=O), 1345 (C-N). ^1H NMR (600 MHz, DMSO) δ (ppm): 8.08 (s, 1H, H-6), 7.89 (s, 1H, H-3'), 7.80 (d, *J* = 3.9 Hz, 1H, H-4), 7.18 (d, *J* = 3.9 Hz, 1H, H-3), 4.73 (s, 2H, H-1'), 4.48 (s, 2H, H-12), 4.31 (t, *J* = 7.1 Hz, 2H, H-1"), 1.77 (dd, *J* = 14.4, 7.2 Hz, 2H, H-2"), 1.34 – 1.01 (m, 18H, H-3"…-11"), 0.88 – 0.85 (m, 3H, H-12"). ^{13}C NMR (151 MHz, DMSO) δ (ppm): 167.4 (C-11), 152.8 (C-9), 152.2 (C-5), 151.4 (C-2), 132.6 (C-6), 132.4 (C-2'), 123.7 (C-3'), 115.3 (C-4), 115.2 (C-3), 50.0 (C-1"), 48.6 (C-1'), 40.4 (C-2"), 39.6 (C-3"), 31.8 (C-10"), 30.3 (C-5"…8"), 29.5 (C-4"), 29.43 (C-9"), 26.0 (C-3"), 22.6 (C-11"), 14.4 (C-12"). Purity 91%. HRMS *m/z* $[M+H]^+$: 488.2566 (calcd. for $C_{23}H_{34}N_7O_5^+$ 488.2621).

IR

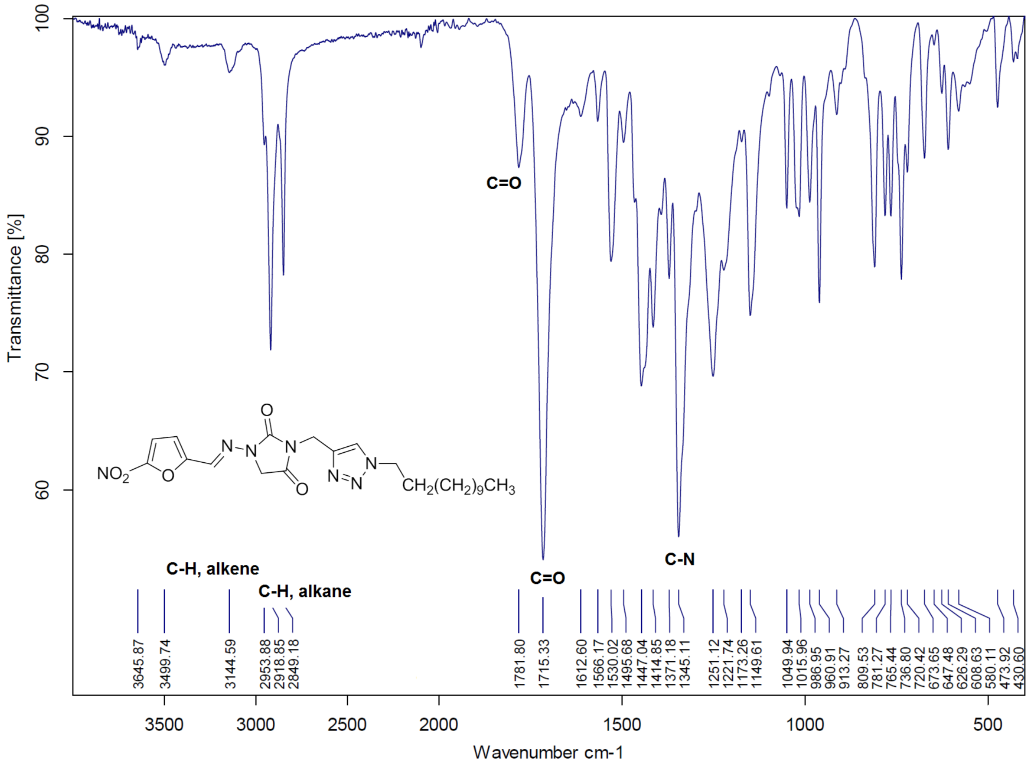

^{1}H-NMR

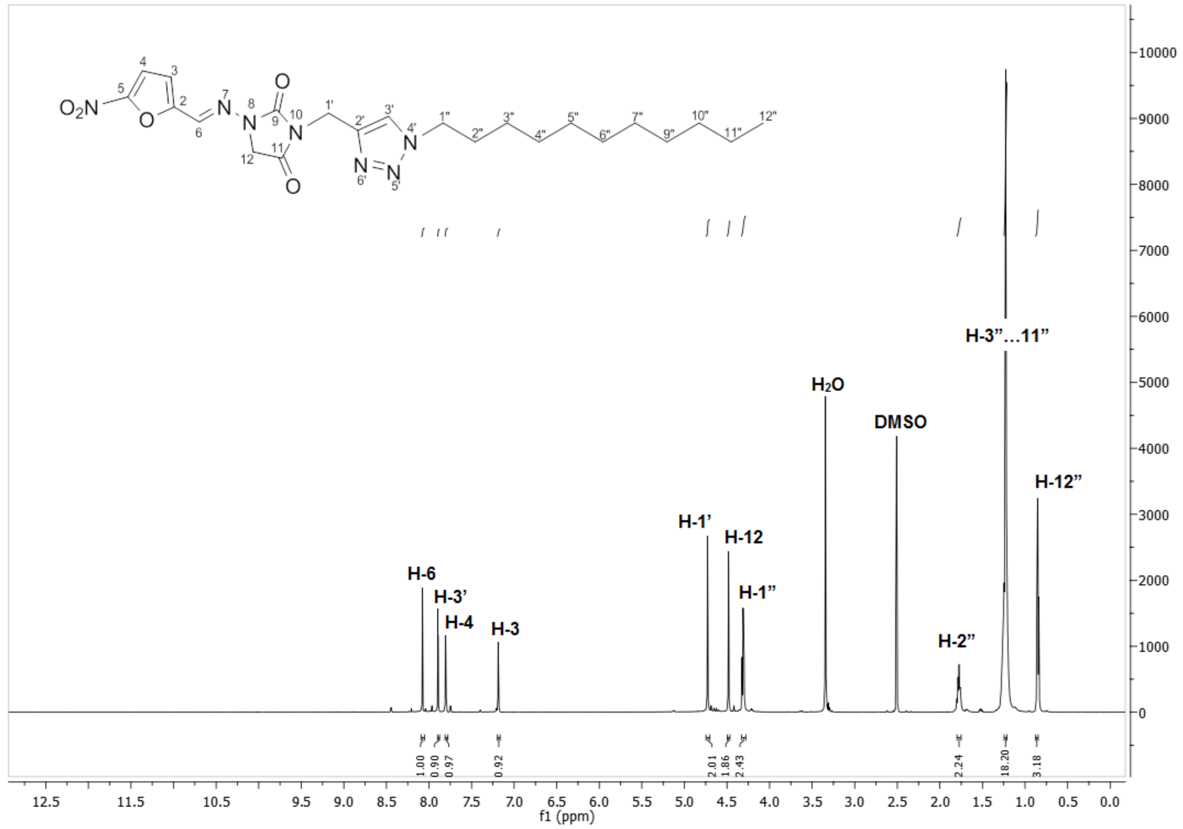

^{13}C-NMR

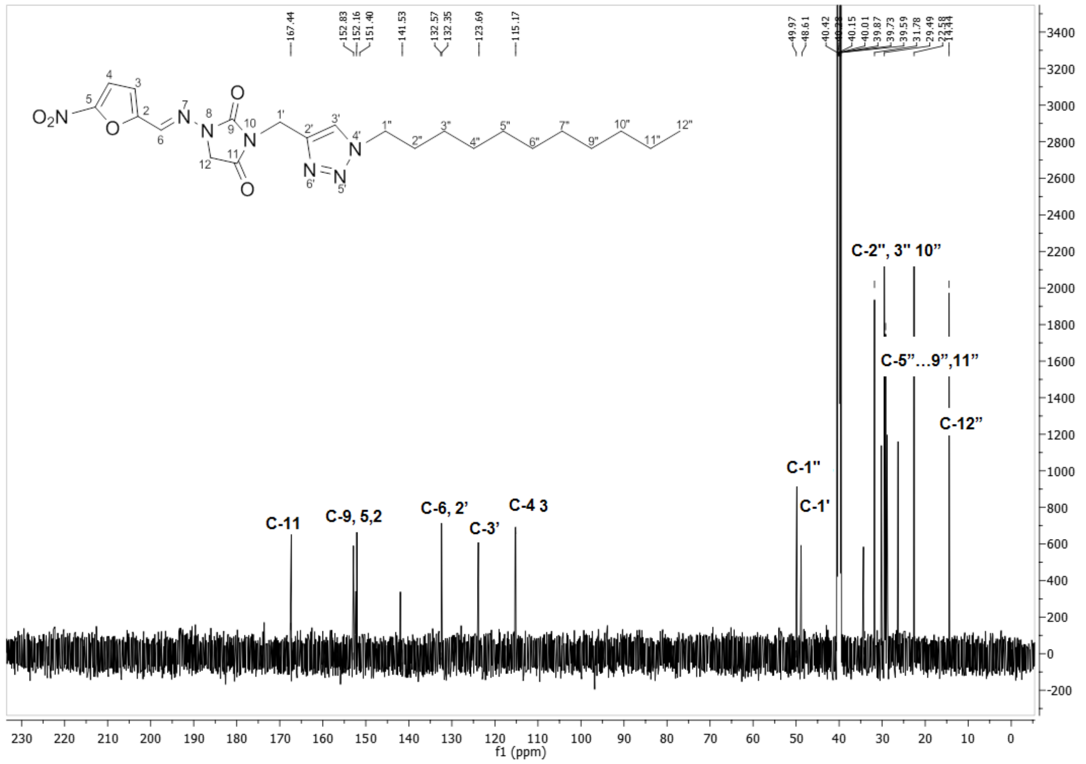

HRMS

Acquisition Parameter

Source Type	APCI	Ion Polarity	Positive	Set Nebulizer	1.6 Bar
Focus	Not active	Set Capillary	4500 V	Set Dry Heater	200 °C
Scan Begin	50 m/z	Set End Plate Offset	-500 V	Set Dry Gas	8.0 l/min
Scan End	1500 m/z	Set Collision Cell RF	100.0 Vpp	Set Divert Valve	Waste

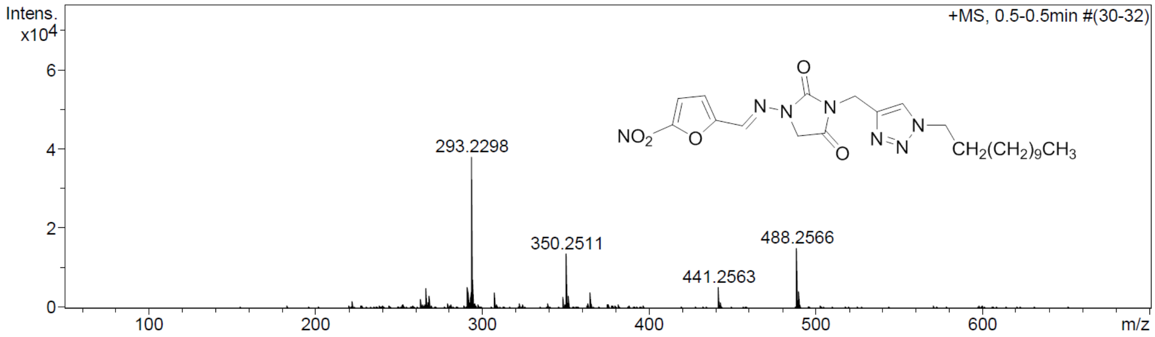

HPLC Purity

2022/03/31 08:37:42 Page 1 / 1

SHIMADZU LabSolutions **Analysis Report**

<Sample Information>

Sample Name	: David NDa S9		
Sample ID	: David NDa S9		
Data Filename	: David NDa S9_012.lcd		
Method Filename	: PURITY non-polar.lcm		
Batch Filename	: David NDa.lcb		
Vial #	: 1-10	Sample Type	: Unknown
Injection Volume	: 1 uL		
Date Acquired	: 29/03/2022 11:00:20	Acquired by	: System Administrator
Date Processed	: 29/03/2022 11:13:21	Processed by	: System Administrator

<Chromatogram>

mAU

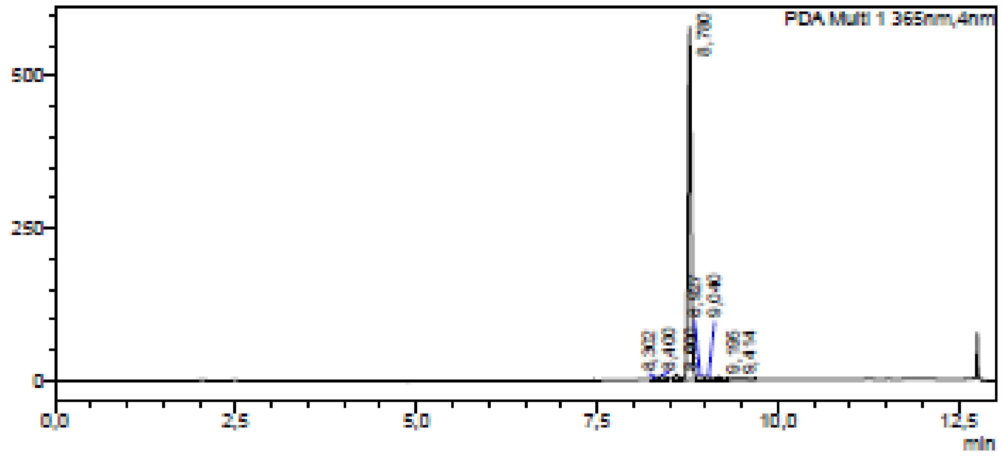

<Peak Table>

PDA Ch1 365nm

Peak#	Ret. Time	Area	Area%
1	8,302	18092	0,958
2	8,400	29197	1,545
3	8,600	30676	1,624
4	8,780	1713513	90,695
5	8,927	30784	1,629
6	9,040	32714	1,732
7	9,195	15364	0,813
8	9,414	18972	1,004
Total		1889311	100,000

C:\LabSolutions\Data\Project1\PURITY\David NDa S9_012.lcd

(E)-1-{[(5-Nitrofuran-2-yl)methylene]amino}-10-[(1-(4-nitrophenethyl)-1H-1,2,3-triazol-4-yl)methyl]imidazolidine-9,11-dione, ***10***

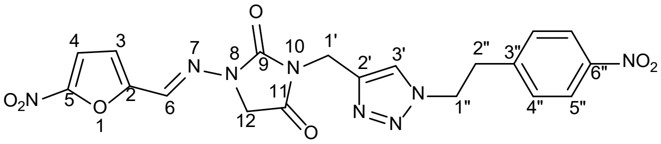

The reaction of **1** with 1-(2-azidoethyl)-4-nitrobenzene yielded **10** as a dark orange powder, 334 mg (39%), mp: 125.4-126.1 °C (EtOH/H_2O). IR (ATR) ν_{max}/cm^{-1}: 3240 (C-H, alkene), 2921 (C-H, alkane), 1783 (C=O), 1726 (C=O), 1339 (C-N). ^{1}H NMR (600 MHz, DMSO) δ (ppm): 8.16 (d, *J* = 8.7 Hz, 2H, H-5"), 8.05 (s, 1H, H-6), 7.89 (s, 1H, H-3'), 7.80 (d, *J* = 3.8 Hz, 1H, H-4), 7.46 (d, *J* = 8.7 Hz, 2H, H-4"), 7.19 (d, *J* = 3.8 Hz, 1H, H-3), 4.49 (s, 2H, H-12), 4.29 (d, *J* = 7.4 Hz, 2H, H-1"), 3.30 (t, *J* = 7.4 Hz, 2H, H-2"), ^{13}C NMR (151 MHz, DMSO) δ (ppm): 166.9 (C-11), 152.3 (C-9), 152.3 (C-5), 152.0 (C-2), 143.9 (C-6"), 142.9 (C-3"), 132.6 (C-6), 130.6 (C-4"), 123.9 (C-3'), 115.5 (C-4), 115.1 (C-3), 48.9 (C-1"), 39.6 (C-2"). Purity: not determined. HRMS *m/z* [M+H]$^+$: 469.1232 (calcd. for $C_{19}H_{17}N_8O_7^+$ 469.1220).

IR

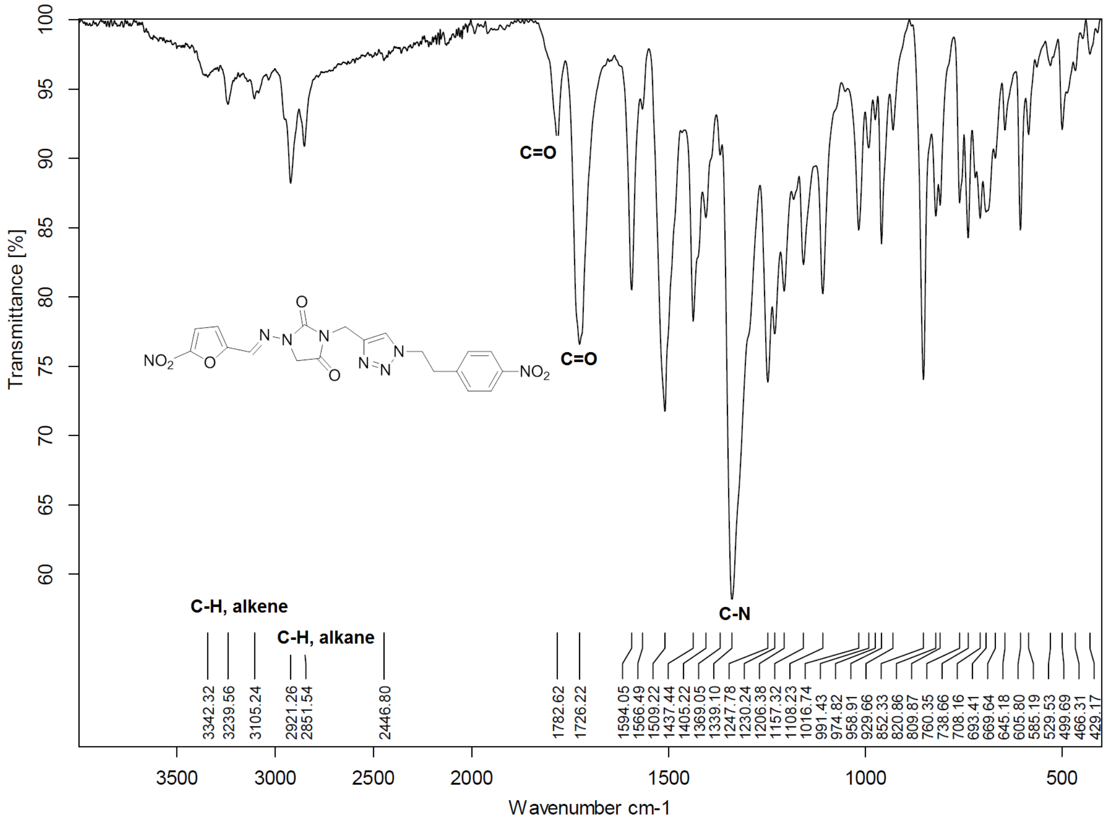

^{13}C-NMR

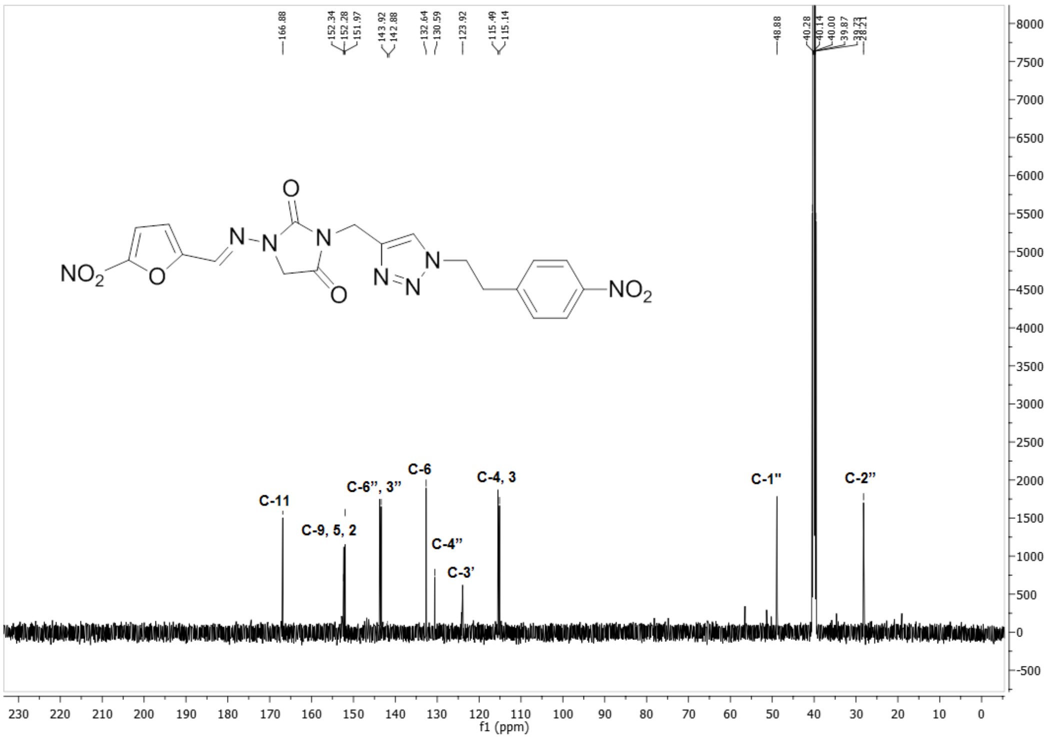

HRMS

Acquisition Parameter

Source Type	APCI	Ion Polarity	Positive	Set Nebulizer	1.6 Bar
Focus	Not active	Set Capillary	4500 V	Set Dry Heater	200 °C
Scan Begin	50 m/z	Set End Plate Offset	-500 V	Set Dry Gas	8.0 l/min
Scan End	1600 m/z	Set Collision Cell RF	80.0 Vpp	Set Divert Valve	Waste

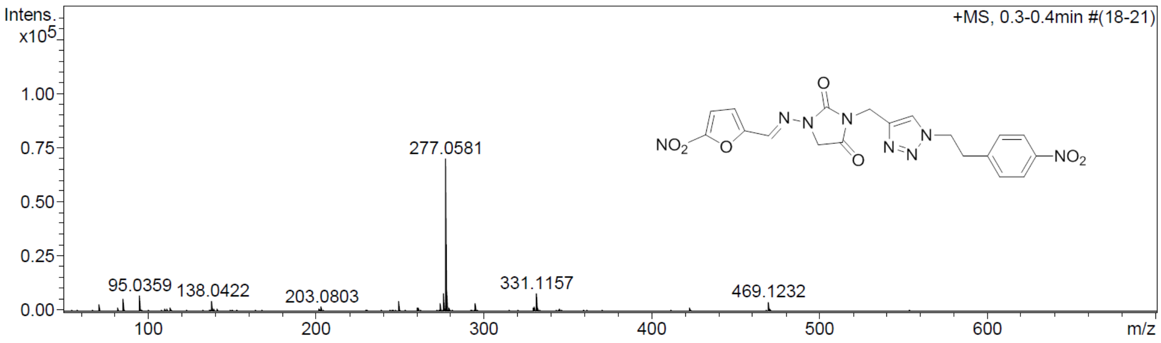

(E)-10-[(4-Benzyl-1H-1,2,3-triazolyl)methyl]-1-{[(5-nitrofuran-2-yl)methylene]amino} imidazolidine-9,11-dione, ***11***

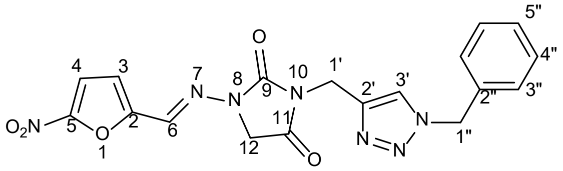

The reaction of **1** with (azidomethyl)benzene provided **11** as an orange powder, 523 mg (71%), mp: 143.5-145.4 °C (EtOH/H_2O). IR (ATR) ν_{max}/cm^{-1}: 3130 (C-H, alkene), 2921 (C-H, alkane), 2851 (C-H, alkane), 1781 (C=O), 1714 (C=O), 1343 (C-N). ^{1}H NMR (600 MHz, DMSO) δ (ppm): 8.14 (s, 1H, H-6), 7.88 (s, 1H, H-3'), 7.80 (d, *J* = 3.9 Hz, 1H, H-4), 7.41 – 7.30 (m, 5H, H-2"…5"), 7.18 (d, *J* = 3.9 Hz, 1H, H-3), 5.58 (s, 2H, H-1"), 4.73 (s, 2H, H-1'), 4.47 (s, 2H, H-12). ^{13}C NMR (151 MHz, DMSO) δ (ppm): 167.7 (C-11), 152.6 (C-9), 152.4 (C-5), 152.1 (C-2), 142.5 (C-2"), 136.1 (C-2'), 132.40 (C-4"), 128.7 (C-3"), 124.1 (C-3') 115.3 (C-4), 115.0 (C-3), 78.4 (C-2'), 74.8 (C-3'), 53.3 (C-1"), 48.4 (C-12). Purity: 98%. HRMS *m/z* [M+H]$^+$: 410.1170 (calcd. for $C_{18}H_{16}N_7O_5^+$ 410.1213).

IR

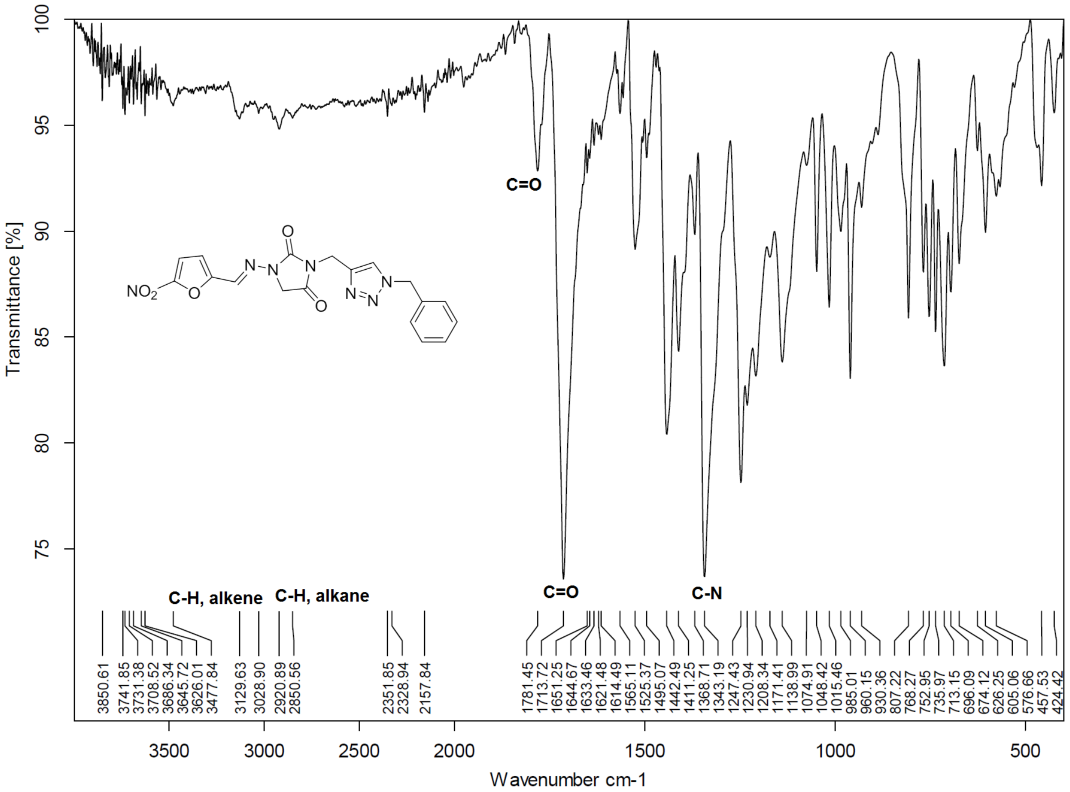

^{1}H-NMR

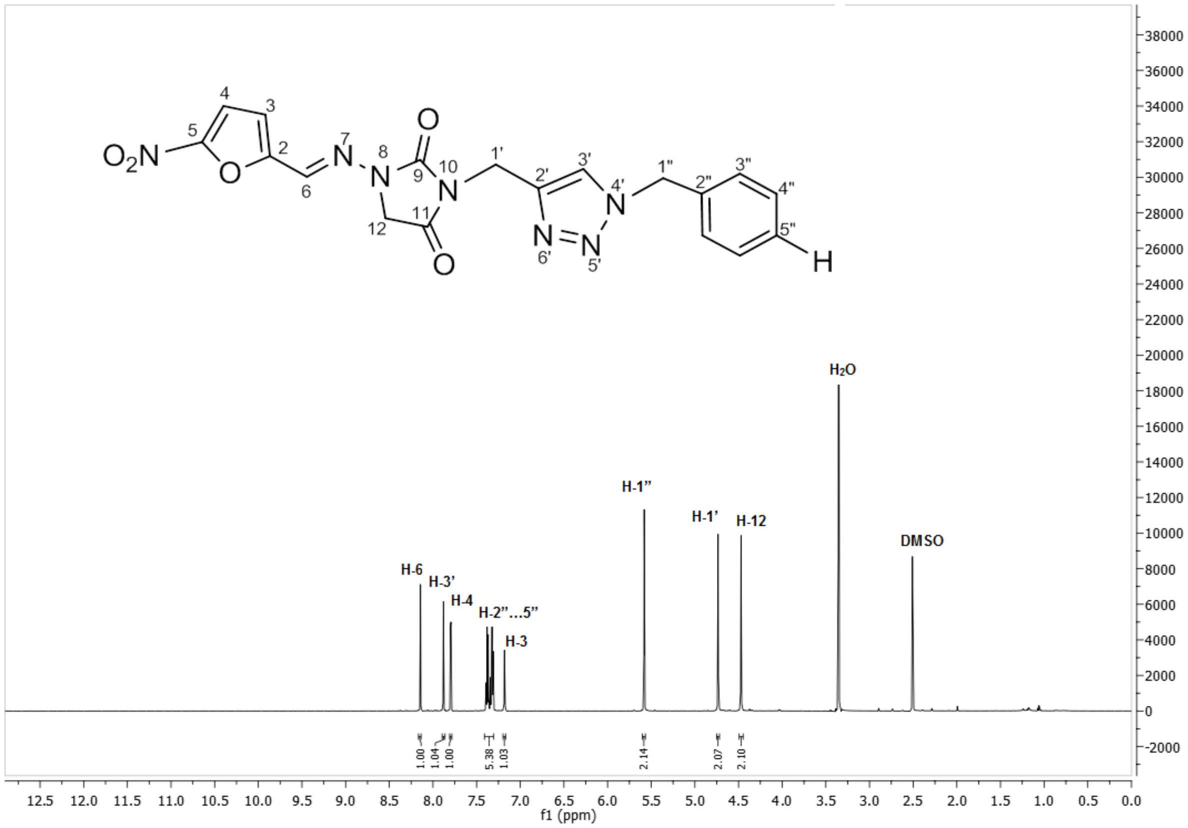

^{13}C-NMR

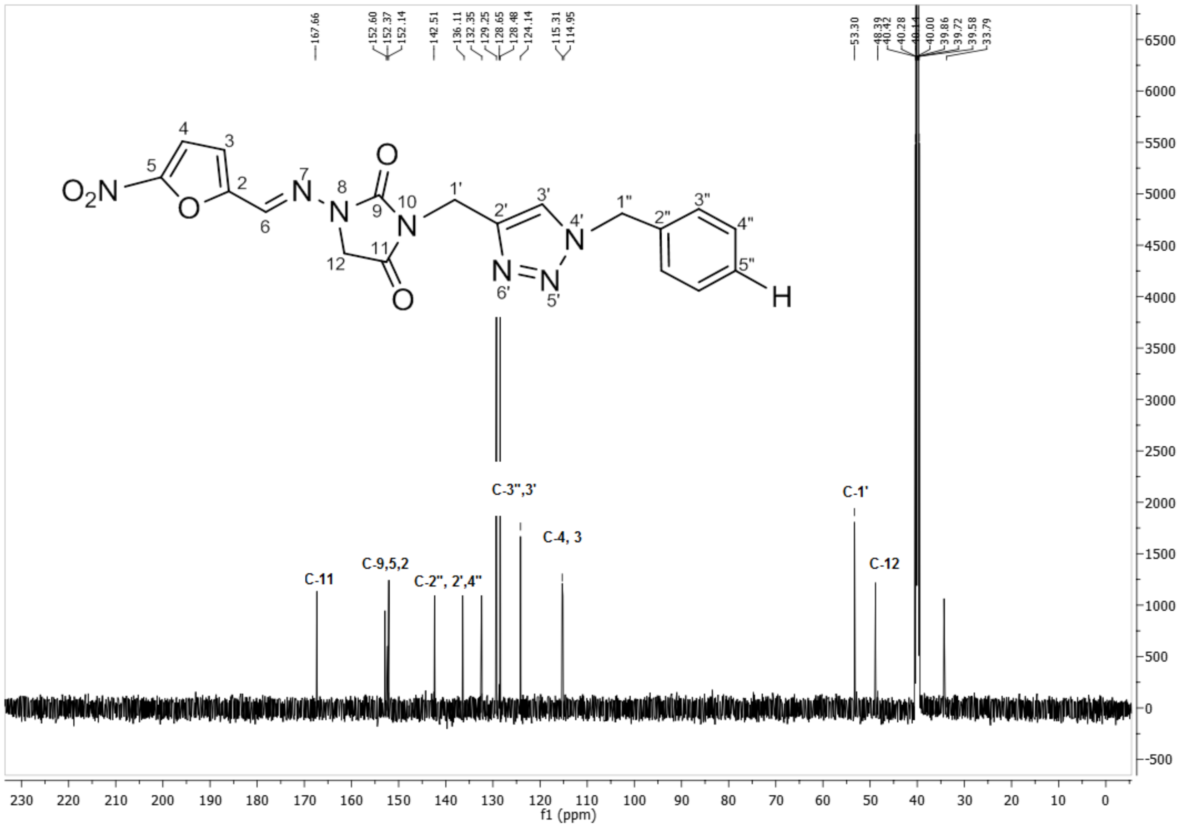

HRMS

Acquisition Parameter

Source Type	APCI	Ion Polarity	Positive	Set Nebulizer	1.6 Bar
Focus	Not active	Set Capillary	4500 V	Set Dry Heater	200 °C
Scan Begin	50 m/z	Set End Plate Offset	-500 V	Set Dry Gas	8.0 l/min
Scan End	1500 m/z	Set Collision Cell RF	100.0 Vpp	Set Divert Valve	Waste

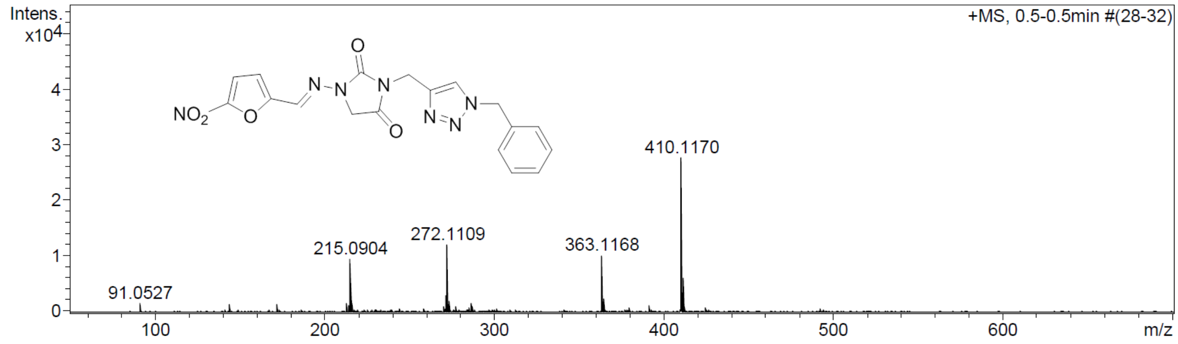

HPLC Purity

SHIMADZU LabSolutions

Analysis Report

<Sample Information>

Sample Name	: David NDa S11		
Sample ID	: David NDa S11		
Data Filename	: David NDa S11_014.lcd		
Method Filename	: PURITY non-polar.lcm		
Batch Filename	: David NDa.lcb		
Vial #	: 1-12	Sample Type	: Unknown
Injection Volume	: 1 uL		
Date Acquired	: 29/03/2022 11:27:00	Acquired by	: System Administrator
Date Processed	: 29/03/2022 11:40:01	Processed by	: System Administrator

<Chromatogram>

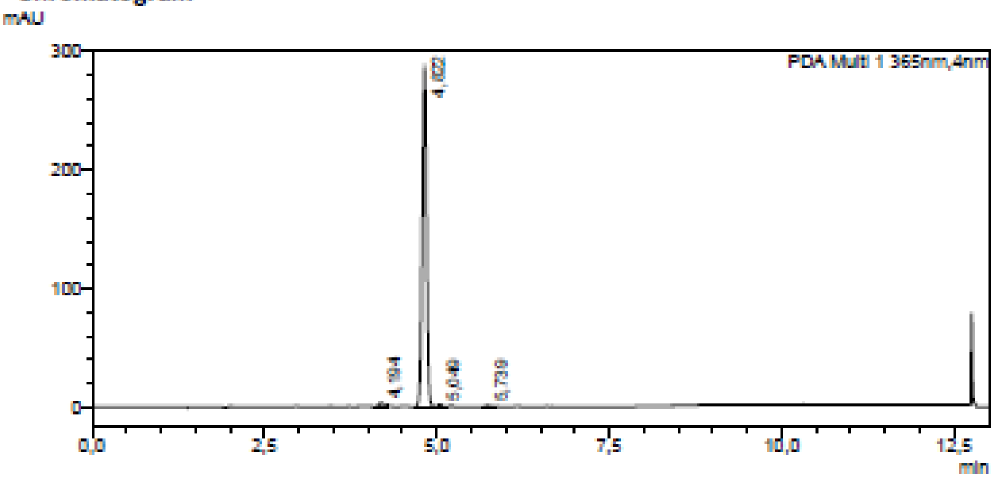

<Peak Table>

PDA Ch1 365nm

Peak#	Ret. Time	Area	Area%
1	4,194	16867	1,342
2	4,822	1226318	97,591
3	5,049	7301	0,581
4	5,739	6102	0,486
Total		1256588	100,000

C:\LabSolutions\Data\Project1\PURITY\David NDa S11_014.lcd

(E)-10-[(5-Methylbenzyl-1H-1,2,3-triazolyl)methyl]-1-{[(5-nitrofuran-2-yl)methylene] amino}imidazolidine-9,11-dione, ***12***

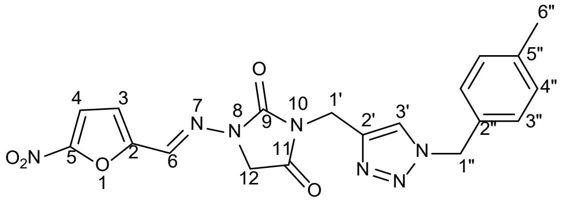

1 and 1-(azidomethyl)-4-methylbenzene yielded **12** as a fluffy, shiny and yellow crystals, 424 mg (55%), mp: 154.9-155.2 °C (EtOH/H_2O). IR (ATR) ν_{max}/cm^{-1}: 3155 (C-H, alkene), 2918 (C-H, alkane), 1780 (C=O), 1719 (C=O), 1345 (C-N). ^{1}H NMR (600 MHz, DMSO) δ (ppm): 8.09 (s, 1H, H-6), 7.88 (s, 1H, H-3'), 7.80 (d, *J* = 3.9 Hz, 1H, H-4), 7.21-7.17 (m, 5H, H-3, 3", 4"), 5.52 (s, 2H, H-1"), 4.72 (s, 2H, H-1'), 4.47 (s, 2H, H-12), 2.28 (s, 3H, H-6"). ^{13}C NMR (151 MHz, DMSO) δ (ppm): 167.0 (C-11), 152.8 (C-9), 152.4 (C-2), 151.6 (C-5), 138.0 (C-2"), 133.6 (C-6), 132.8 (C-2'), 129.8 (C-4"), 128.6 (C-3"), 123.7 (C-3'), 115.3 (C-4), 115.2 (C-3), 53.1 (C-1"), 48.8 (C-12), 40.4 (C-1'), 21.2 (C-6"). Purity: 97%. HRMS *m/z* [M+H]$^+$: 424.1323 (calc. for $C_{19}H_{18}N_7O_5^+$ 424.1369).

IR

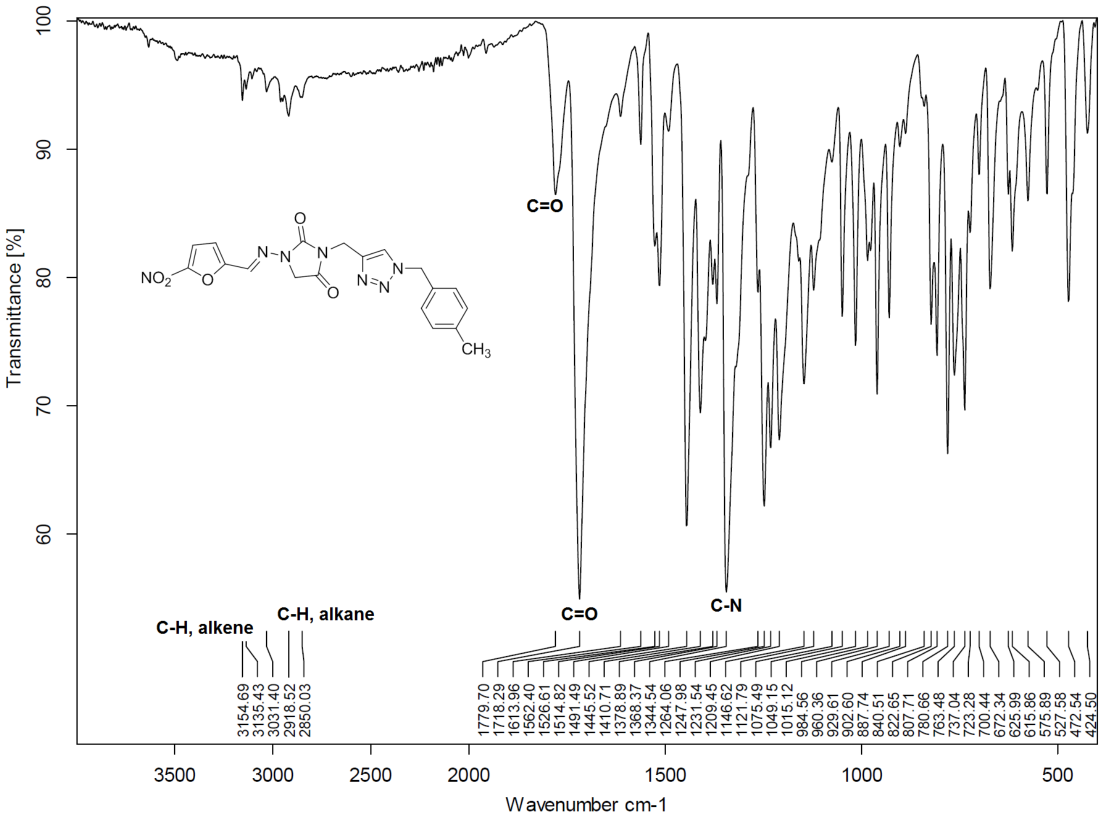

^{1}H-NMR

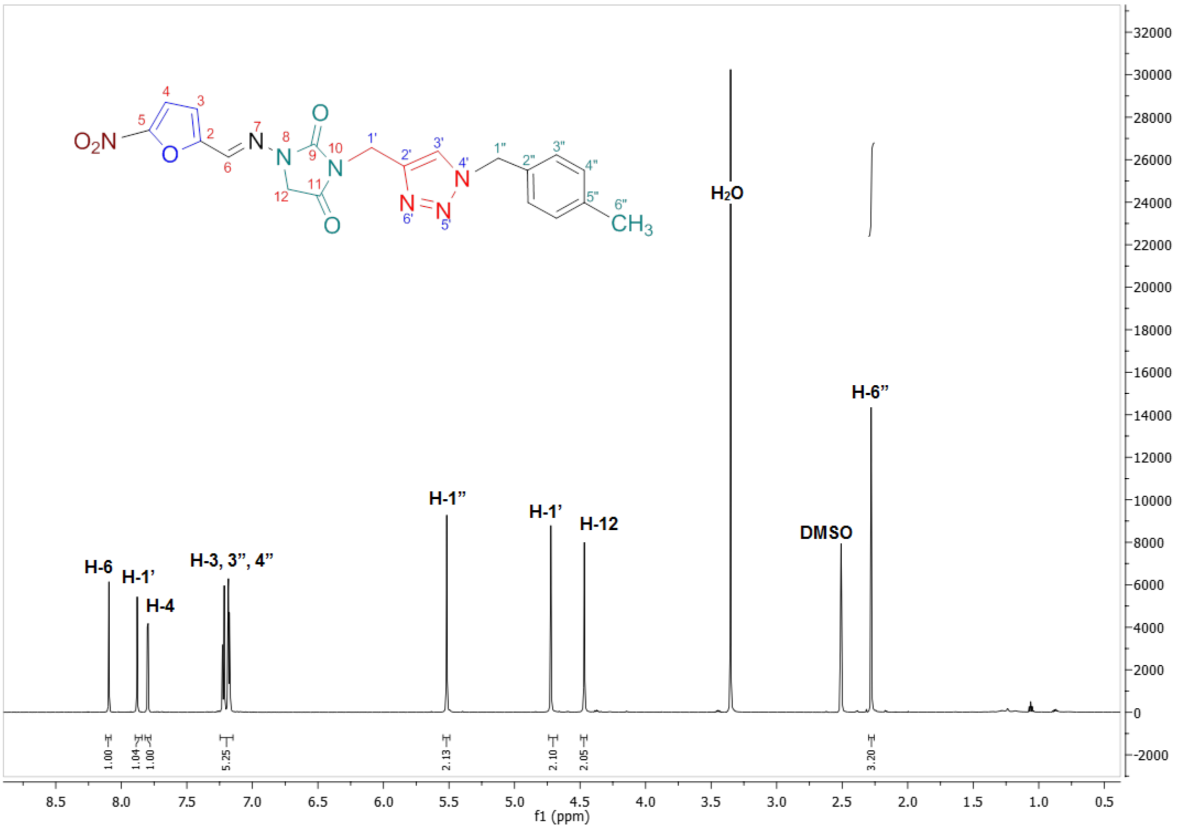

^{13}C-NMR

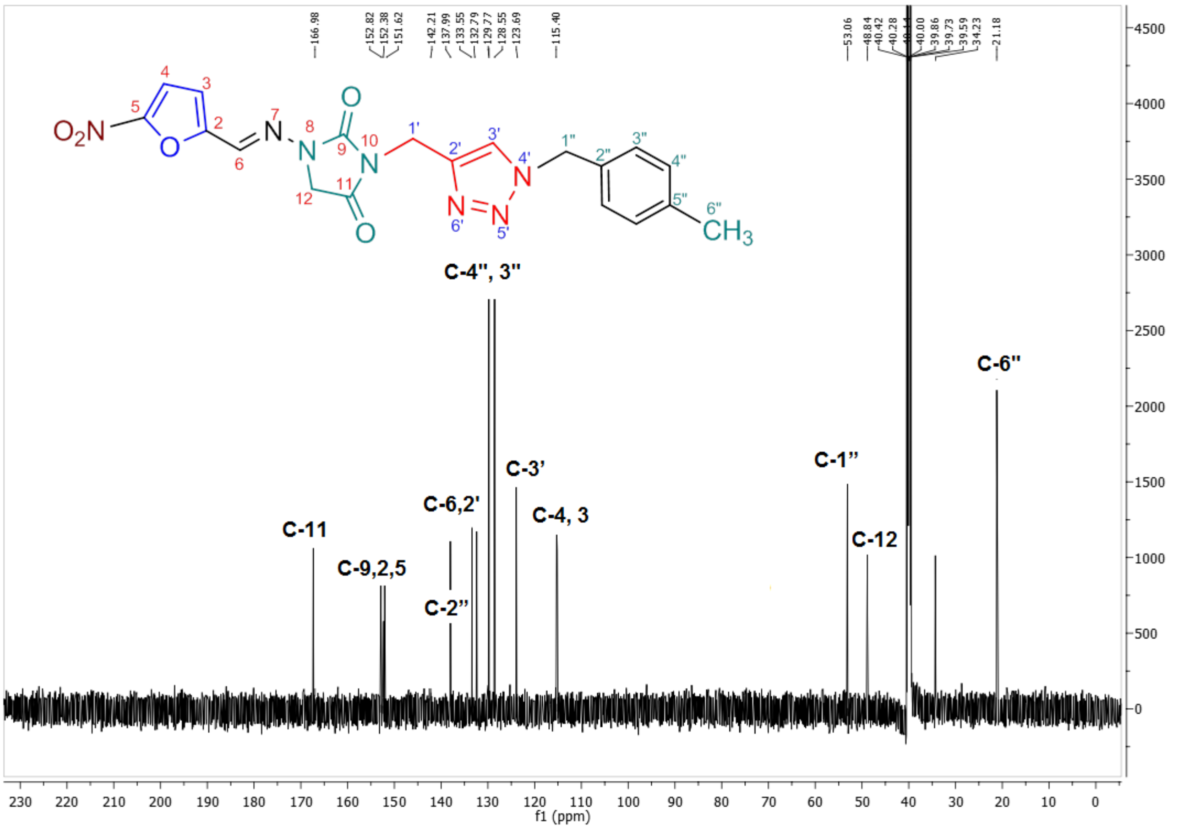

HRMS

Acquisition Parameter

Source Type	APCI	Ion Polarity	Positive	Set Nebulizer	1.6 Bar
Focus	Not active	Set Capillary	4500 V	Set Dry Heater	200 °C
Scan Begin	50 m/z	Set End Plate Offset	-500 V	Set Dry Gas	8.0 l/min
Scan End	1500 m/z	Set Collision Cell RF	100.0 Vpp	Set Divert Valve	Waste

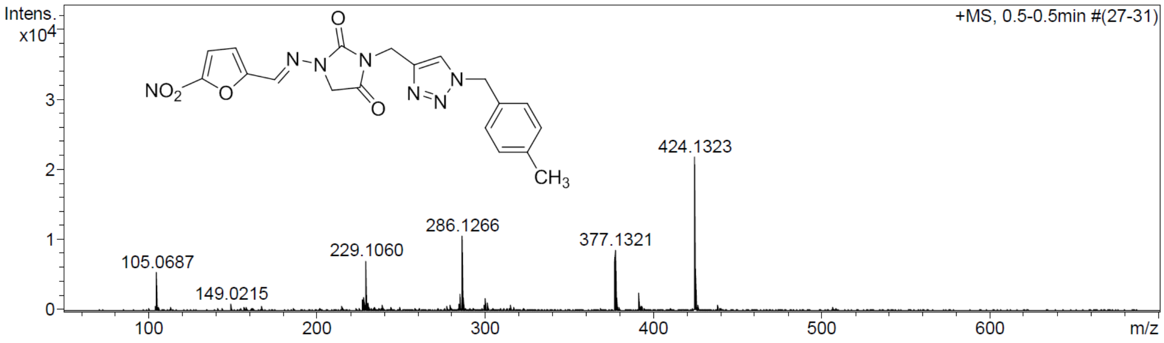

HPLC Purity

2022/03/31 08:43:25 Page 1 / 1

Analysis Report

SHIMADZU LabSolutions

<Sample Information>

Sample Name	: David NDa S12		
Sample ID	: David NDa S12		
Data Filename	: David NDa S12_015.lcd		
Method Filename	: PURITY non-polar.lcm		
Batch Filename	: David NDa.lcb		
Vial #	: 1-13	Sample Type	: Unknown
Injection Volume	: 1 uL		
Date Acquired	: 29/03/2022 11:40:22	Acquired by	: System Administrator
Date Processed	: 29/03/2022 11:53:23	Processed by	: System Administrator

<Chromatogram>

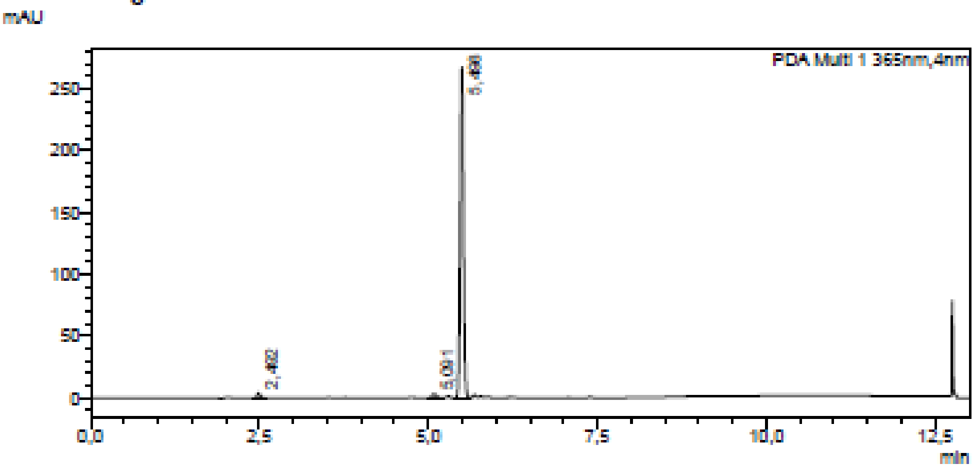

<Peak Table>

PDA Ch1 365nm

Peak#	Ret. Time	Area	Area%
1	2,492	12425	1,378
2	5,091	16108	1,787
3	5,498	872826	96,834
Total		901359	100,000

C:\LabSolutions\Data\Project1\PURITY\David NDa S12_015.lcd

(E)-10-[(5-Isopropylbenzyl-1H-1,2,3-triazolyl)methyl]-1-{[(5-nitrofuran-2-yl)methylene] amino}imidazolidine-9,11-dione, ***13***

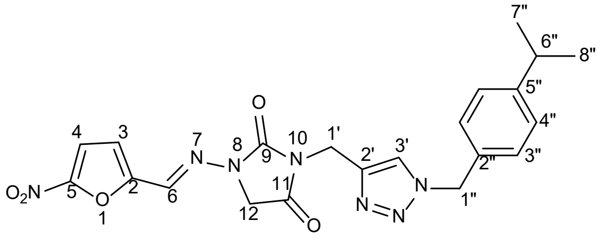

The reaction of **1** with 1-(azidomethyl)-4-isopropylbenzene afforded **13** as an orange powder, 627 mg (77%), mp: 154.9-155.1 °C (EtOH/H_2O). IR (ATR) ν_{max}/cm^{-1}: 3135 (C-H, alkene), 2958 (C-H, alkane), 1783 (C=O), 1714 (C=O), 1344 (C-N). ^{1}H NMR (600 MHz, DMSO) δ (ppm): 8.13 (s, 1H, H-6), 7.88 (s, 1H, H-3'), 7.80 (d, *J* = 3.9 Hz, 1H, H-4), 7.26 – 7.22 (m, 4H, H-3", -4"), 7.18 (d, *J* = 3.9 Hz, 1H, H-3), 5.52 (s, 2H, H-1"), 4.73 (s, 2H, H-1'), 4.47 (s, 2H, H-12), 2.92 – 2.80 (m, 1H, H-6"), 1.17 (d, *J* = 6.9 Hz, 6H, H-7", 8"). ^{13}C NMR (151 MHz, DMSO) δ (ppm): 168.0 (C-11), 152.6 (C-9), 152.0 (C-5), 148.7 (C-2), 142.1 (C-2"), 133.9 (C-2'), 128.6 (C-3") 127.2 (C-4"), 124.1 (C-3'), 115.3 (C-4), 115. 0 (C-3), 52.6 (C-1"), 48.9 (C-12), 24.3 (C-7", -8"). Purity: 70%. HRMS *m/z* [M+H]$^+$: 452.1716 (calcd. for $C_{21}H_{22}N_7O_5^+$, 452.1682).

IR

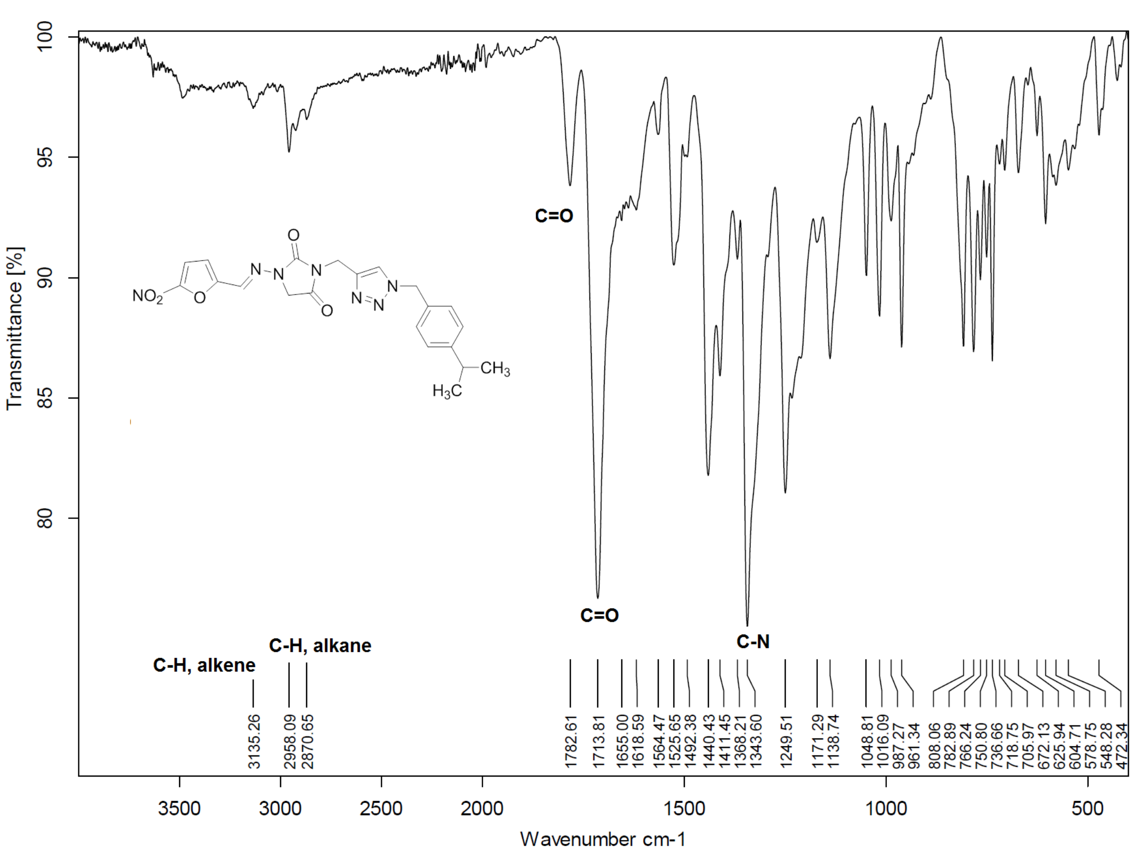

^{1}H-NMR

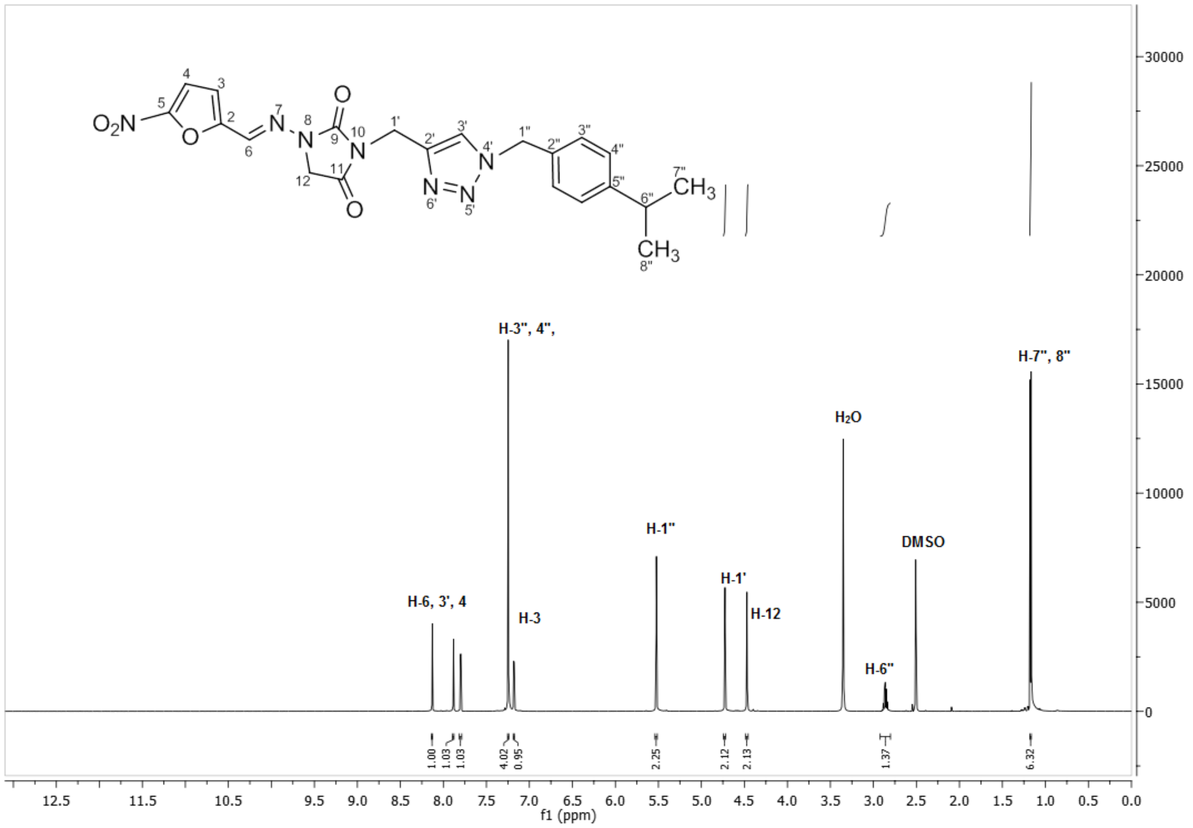

^{13}C-NMR

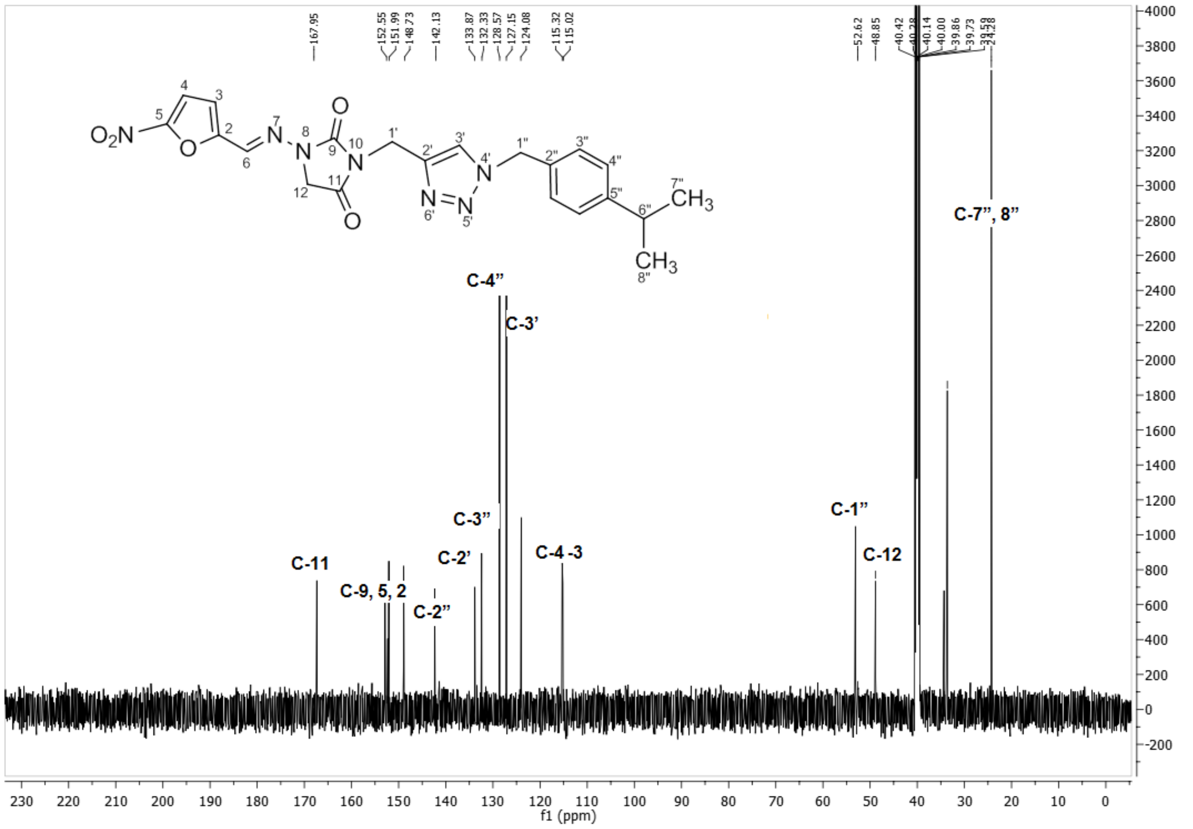

HRMS

Acquisition Parameter

Source Type	APCI	Ion Polarity	Positive	Set Nebulizer	1.6 Bar
Focus	Not active	Set Capillary	4500 V	Set Dry Heater	200 °C
Scan Begin	50 m/z	Set End Plate Offset	-500 V	Set Dry Gas	8.0 l/min
Scan End	1500 m/z	Set Collision Cell RF	100.0 Vpp	Set Divert Valve	Waste

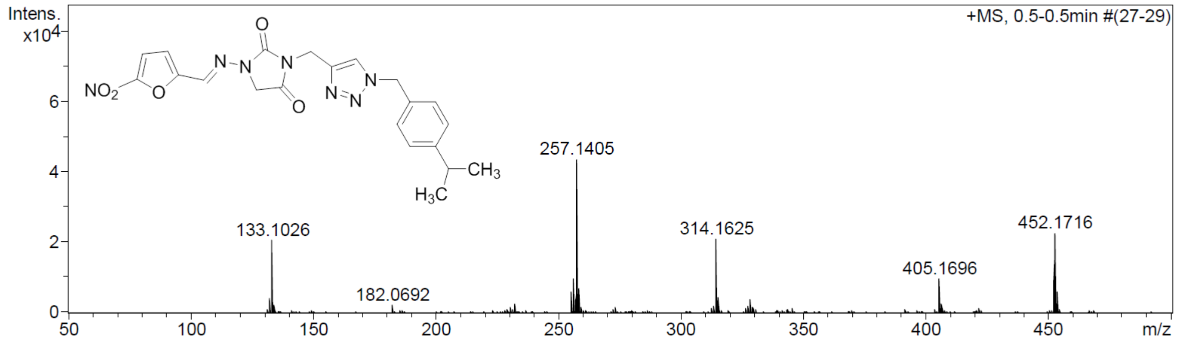

HPLC Purity

2022/03/31 08:45:05 Page 1 / 1

Analysis Report

SHIMADZU LabSolutions

<Sample Information>

Sample Name : David NDa S13
Sample ID : David NDa S13
Data Filename : David NDa S13_016.lcd
Method Filename : PURITY non-polar.lcm
Batch Filename : David NDa.lcb
Vial # : 1-14
Sample Type : Unknown
Injection Volume : 1 uL
Date Acquired : 29/03/2022 11:53:43
Acquired by : System Administrator
Date Processed : 29/03/2022 12:06:44
Processed by : System Administrator

<Chromatogram>

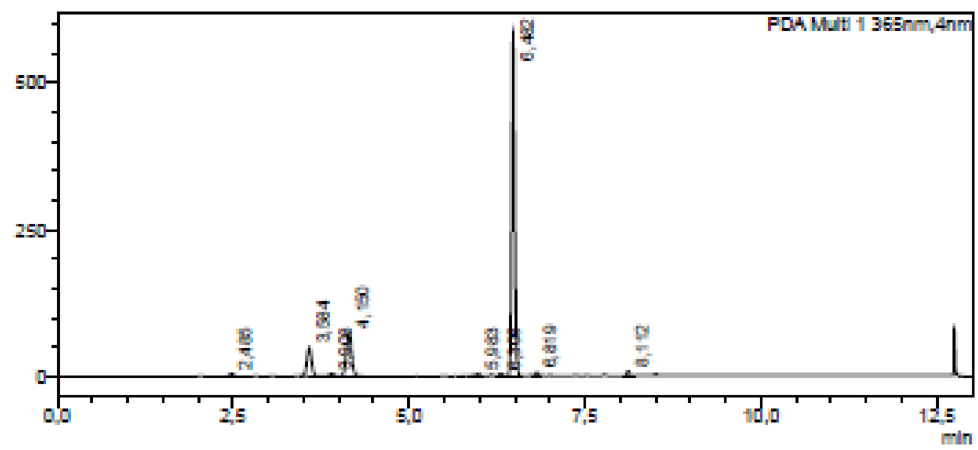

<Peak Table>

PDA Ch1 365nm

Peak#	Ret. Time	Area	Area%
1	2,485	18680	0,763
2	3,584	227649	9,295
3	3,908	21293	0,869
4	4,150	400417	16,349
5	5,983	16406	0,670
6	6,305	18814	0,768
7	6,482	1702946	69,530
8	6,819	21609	0,882
9	8,112	21419	0,875
Total		2449233	100,000

C:\LabSolutions\Data\Project1\PURITY\David NDa S13_016.lcd

(E)-10-{[5-(tert-Butyl)benzyl-1H-1,2,3-triazolyl]methyl}-1-{[(5-nitrofuran-2-yl)methylene]amino}imidazolidine-9,11-dione, ***14***

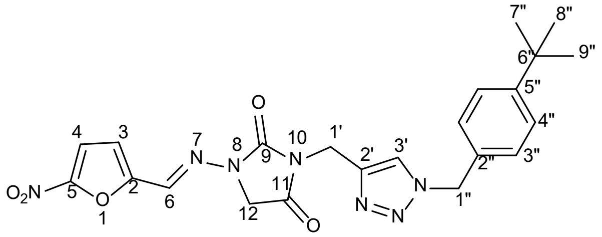

The reaction of **1** with 1-(azidomethyl)-4-(*tert*-butyl)benzene provided **14** as an orange powder, 484 mg (57%), mp: 143.5-145.4 °C (EtOH/H_2O). IR (ATR) ν_{max}/cm^{-1}: 3136 (C-H, alkene), 2960 (C-H, alkane), 1780 (C=O), 1720 (C=O), 1344 (C-N). ^{1}H NMR (600 MHz, DMSO) δ (ppm): 8.13 (s, 1H, H-6), 7.88 (s, 1H, H-3'), 7.80 (d, *J* = 3.9 Hz, 1H, H-4), 7.39 (d, *J* = 8.4 Hz, 2H, H-4"), 7.25 (d, *J* = 8.4 Hz, 2H, H-3"), 7.18 (d, *J* = 3.9 Hz, 1H, H-3), 5.53 (s, 2H, H-1"), 4.73 (s, 2H, H-1'), 4.47 (s, 2H, H-12), 1.25 (s, 9H, H-7"…-9"). ^{13}C NMR (151 MHz, DMSO) δ (ppm): 167.4 (C-11), 152.9 (C-9), 152.1 (C-5), 151.1 (C-2), 142.6 (C-2"), 133.5 (C-2'), 128.3 (C-3") 126.0 (C-4"), 124.0 (C-3'), 115.3 (C-4), 115.2 (C-3), 53.0 (C-1"), 48.6 (C-12), 31.5 (C-7",...-9"). Purity: 84%. HRMS *m/z* [M+H]$^{+}$: 466.1806 (calcd. for $C_{22}H_{24}N_7O_5^{+}$, 466.1839).

IR

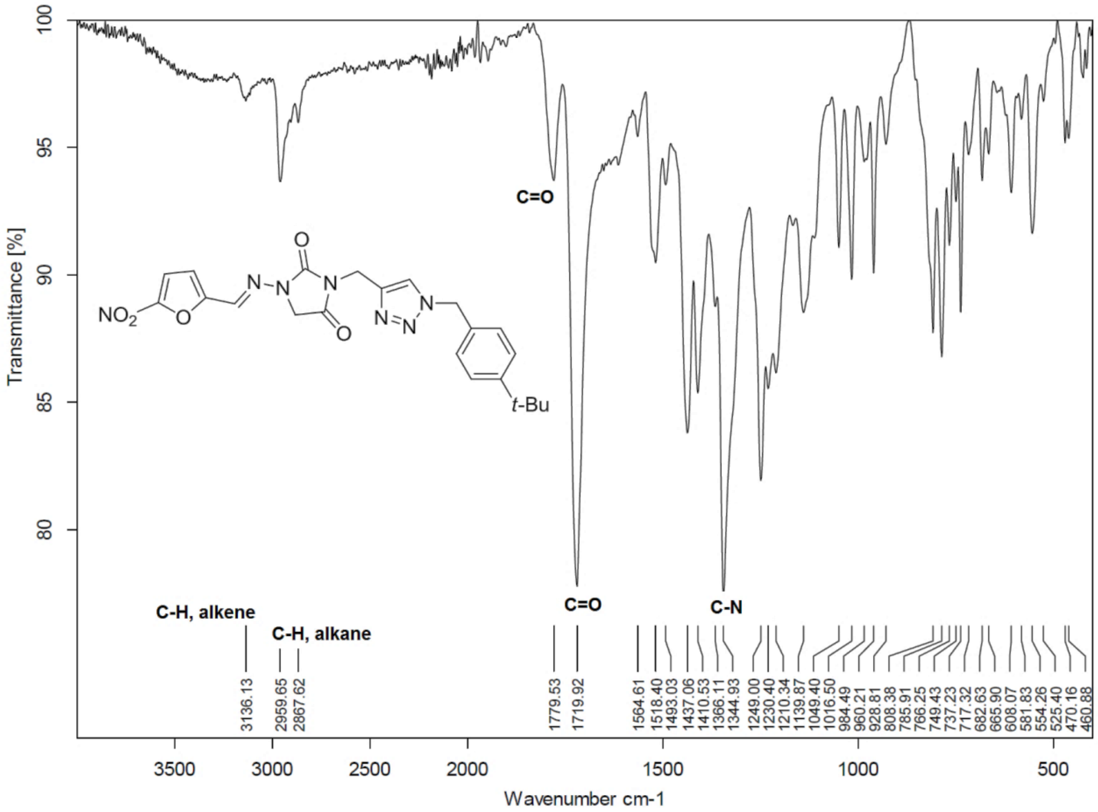

^{1}H-NMR

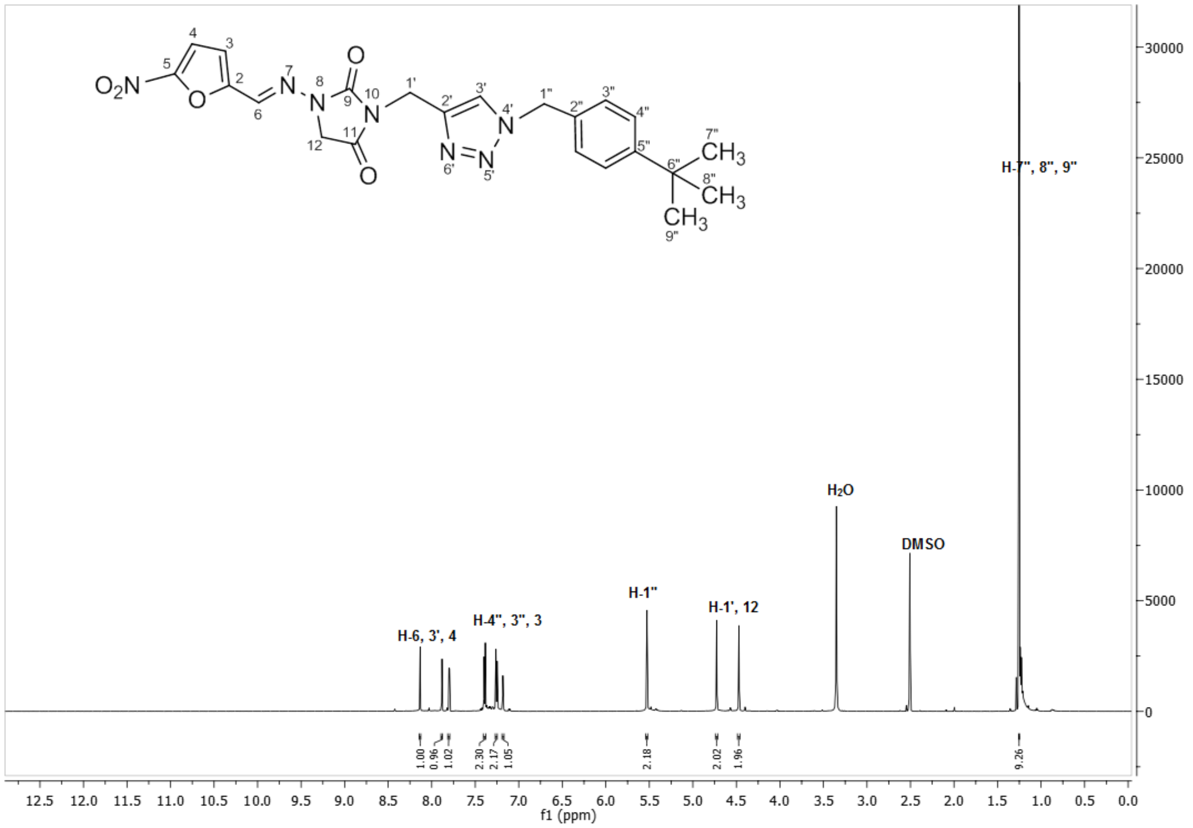

^{13}C-NMR

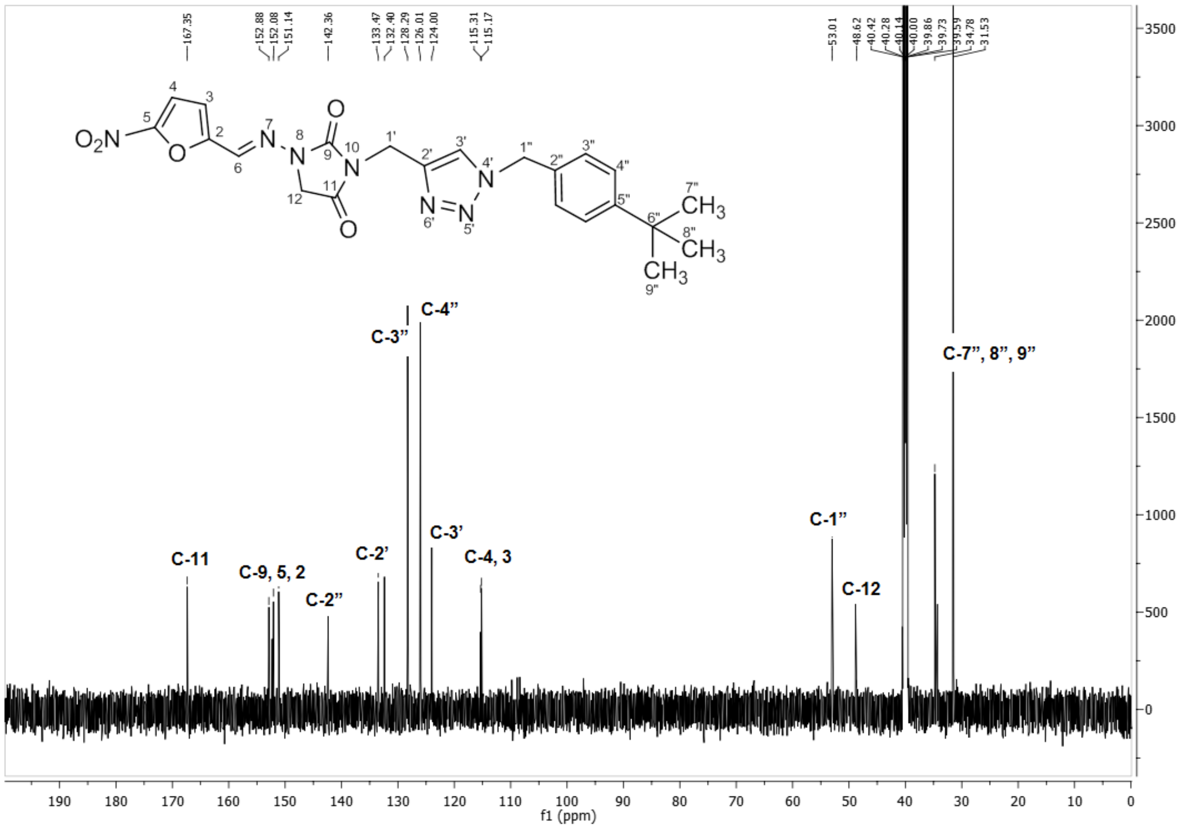

HRMS

Acquisition Parameter

Source Type	APCI	Ion Polarity	Positive	Set Nebulizer	1.6 Bar
Focus	Not active	Set Capillary	4500 V	Set Dry Heater	200 °C
Scan Begin	50 m/z	Set End Plate Offset	-500 V	Set Dry Gas	8.0 l/min
Scan End	1500 m/z	Set Collision Cell RF	100.0 Vpp	Set Divert Valve	Waste

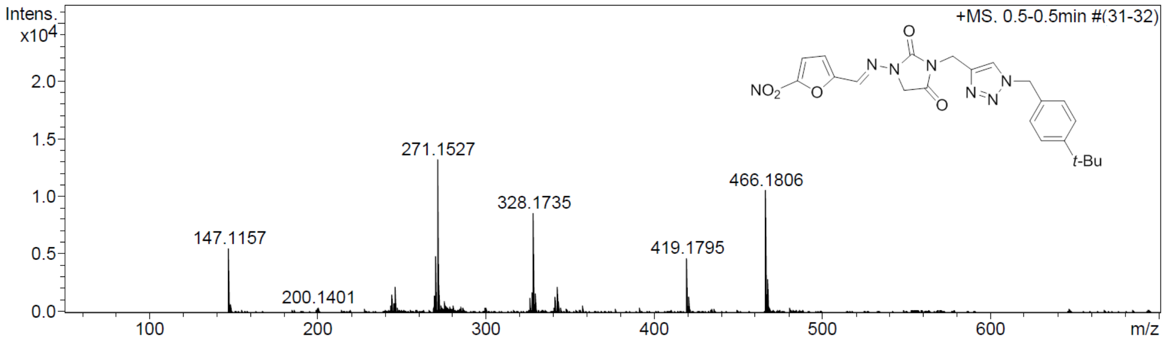

HPLC Purity

2022/03/31 08:47:03 Page 1 / 1

SHIMADZU LabSolutions

Analysis Report

<Sample Information>

Sample Name	: David NDa S14
Sample ID	: David NDa S14
Data Filename	: David NDa S14_017.lcd
Method Filename	: PURITY non-polar.lcm
Batch Filename	: David NDa.lcb
Vial #	: 1-15
Injection Volume	: 1 uL
Date Acquired	: 29/03/2022 12:07:04
Date Processed	: 29/03/2022 12:20:06

Sample Type	: Unknown
Acquired by	: System Administrator
Processed by	: System Administrator

<Chromatogram>

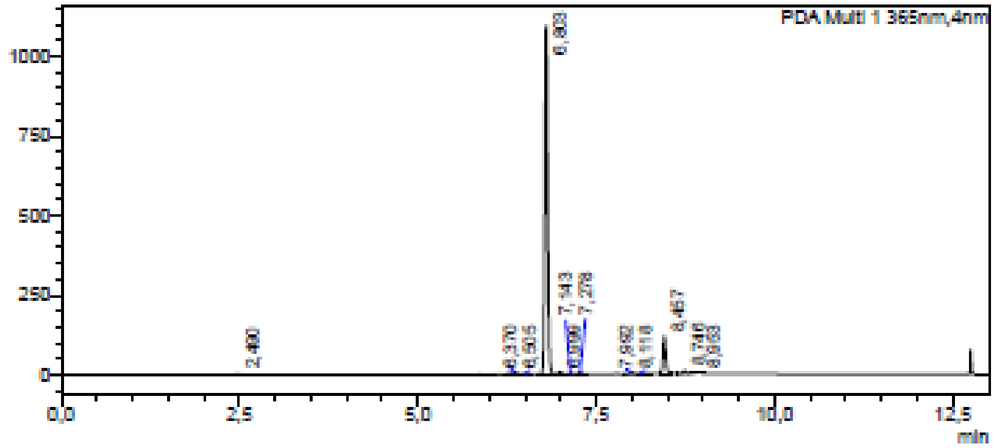

<Peak Table>

PDA Ch1 365nm

Peak#	Ret. Time	Area	Area%
1	2.490	8737	0.238
2	6.370	57074	1.553
3	6.505	10847	0.295
4	6.803	3094492	84.219
5	6.999	36221	0.986
6	7.143	14727	0.401
7	7.278	28561	0.777
8	7.992	22842	0.622
9	8.118	18617	0.507
10	8.457	322640	8.781
11	8.746	30433	0.828
12	8.953	29161	0.794
Total		3674353	100.000

C:\LabSolutions\Data\Project1\PURITY\David NDa S14_017.lcd

(E)-10-[(5-Bromobenzyl-1H-1,2,3-triazolyl)methyl]-1-{[(5-nitrofuran-2-yl)methylene]amino}imidazolidine-9,11-dione, ***15***

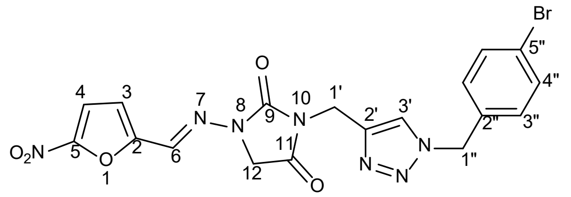

Intermediate **1** and 1-(azidomethyl)-4-bromobenzene afforded **15** as a dark orange powder, 431 mg (49%), mp: 171.7-172.3 °C (EtOH/H_2O). IR (ATR) ν_{max}/cm^{-1}: 3129 (C-H, alkene), 2923 (C-H, alkane), 1785 (C=O), 1713 (C=O), 1344 (C-N). ^{1}H NMR (600 MHz, DMSO) δ (ppm): 8.19 (s, 1H, H-6), 7.88 (s, 1H, H-3'), 7.80 (d, *J* = 3.9 Hz, 1H, H-4), 7.58 (d, *J* = 8.4 Hz, 2H, H-4"), 7.28 (d, *J* = 8.4 Hz, 2H, H-3"), 7.18 (d, *J* = 3.9 Hz, 1H, H-3), 5.60 (s, 2H, H-1"), 4.74 (s, 2H, H-1'), 4.48 (s, 2H, H-12). ^{13}C NMR (151 MHz, DMSO) δ (ppm): 167.4 (C-11), 152.9 (C-9), 152.2 (C-2), 152.1 (C-5), 142.4 (C-2"), 131.6 (C-2'), 131.5 (C-3"), 127.6 (C-4"), 124.4 (C-3'), 122.3 (C-5"), 115.3 (C-4), 115.2 (C-3), 52.2 (C-1"), 49.1 (C-12). Purity: 93%. HRMS *m/z* [M+H]$^+$: 488.0309 (calcd. for $C_{18}H_{15}BrN_7O_5^+$, 488.0318).

IR

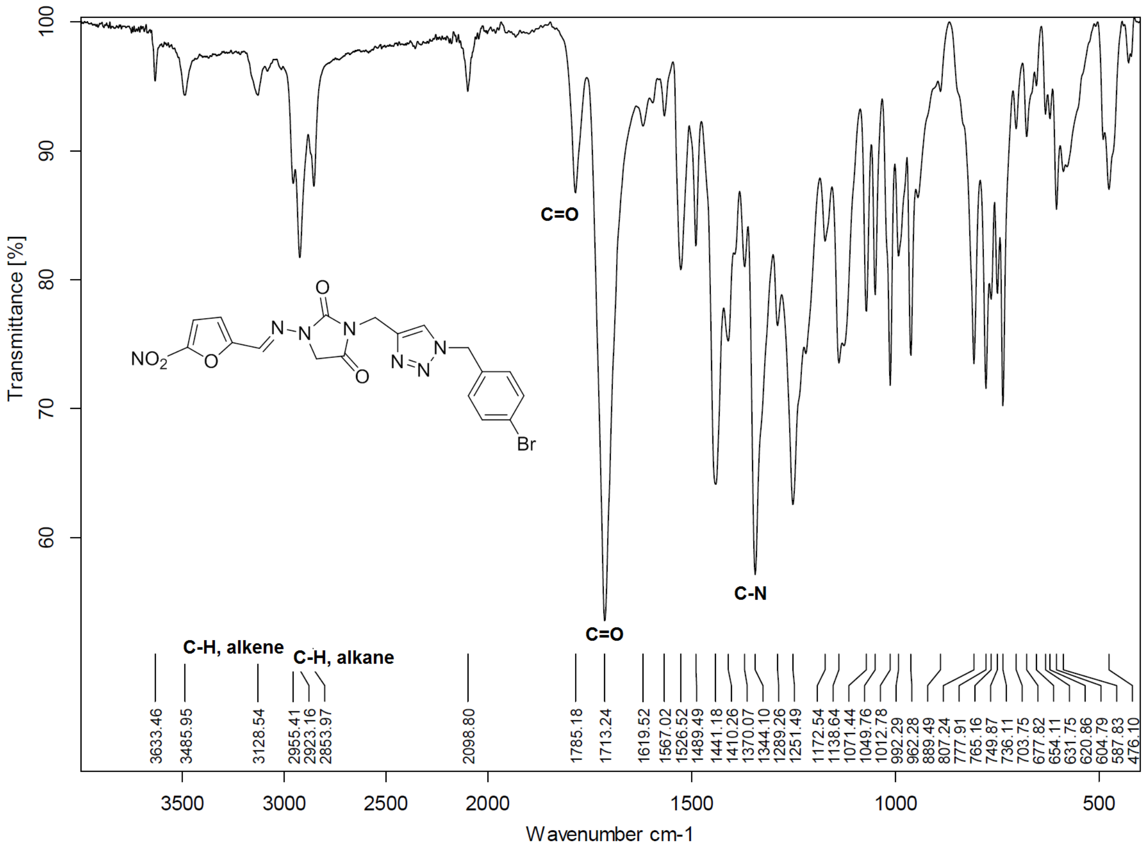

^{1}H-NMR

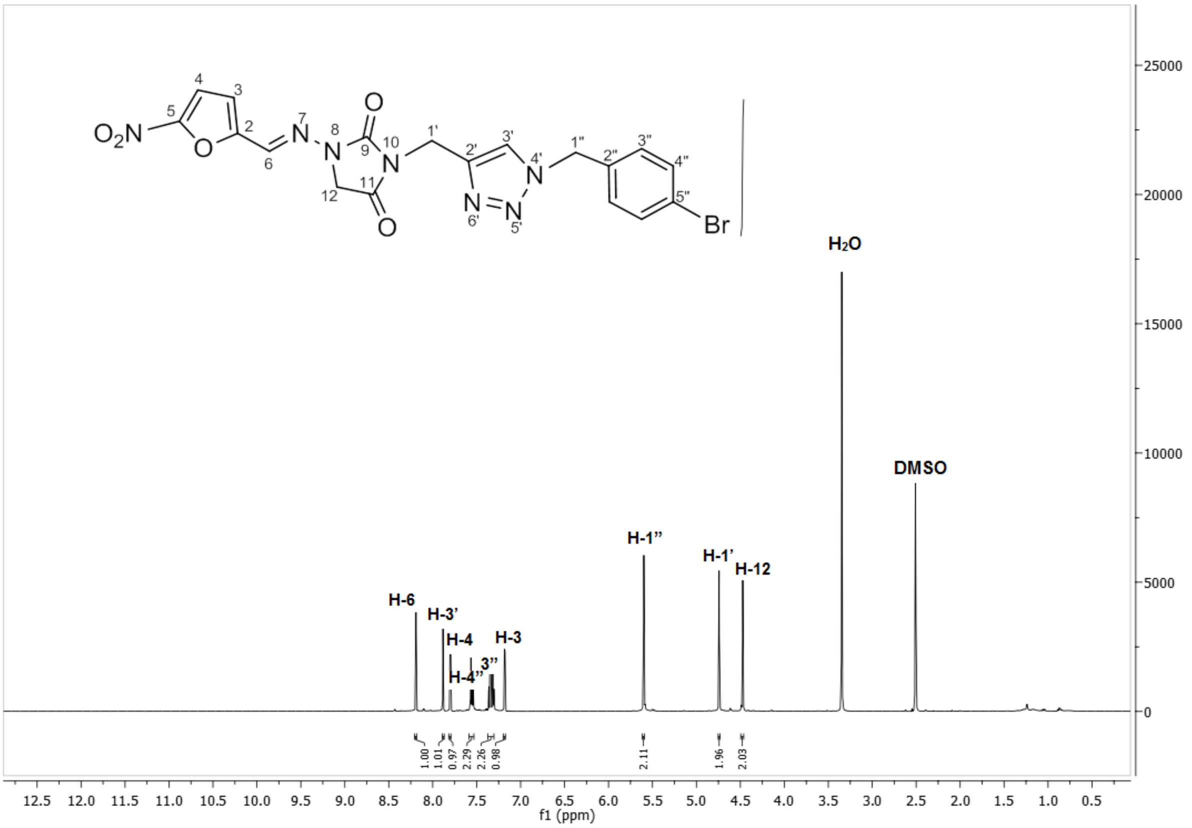

^{13}C-NMR

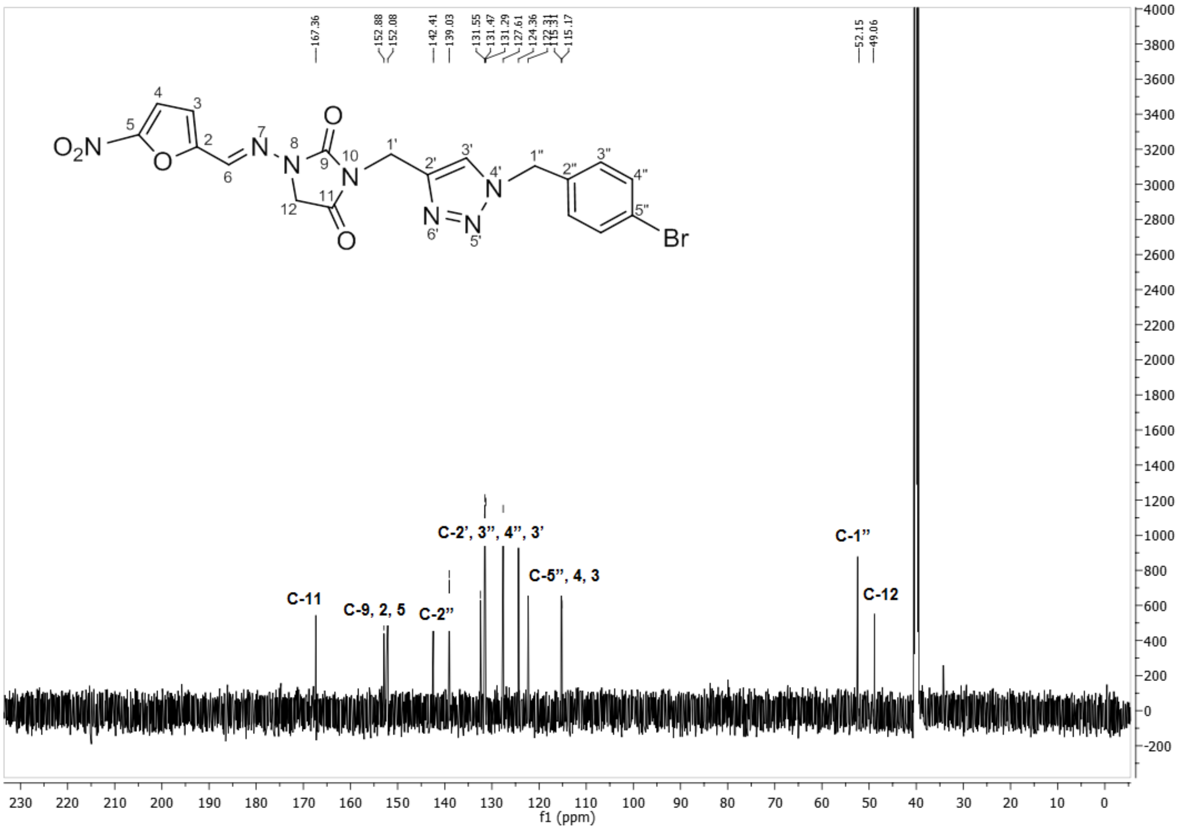

HRMS

Acquisition Parameter

Source Type	APCI	Ion Polarity	Positive	Set Nebulizer	1.6 Bar
Focus	Not active	Set Capillary	4500 V	Set Dry Heater	200 °C
Scan Begin	50 m/z	Set End Plate Offset	-500 V	Set Dry Gas	8.0 l/min
Scan End	1500 m/z	Set Collision Cell RF	100.0 Vpp	Set Divert Valve	Waste

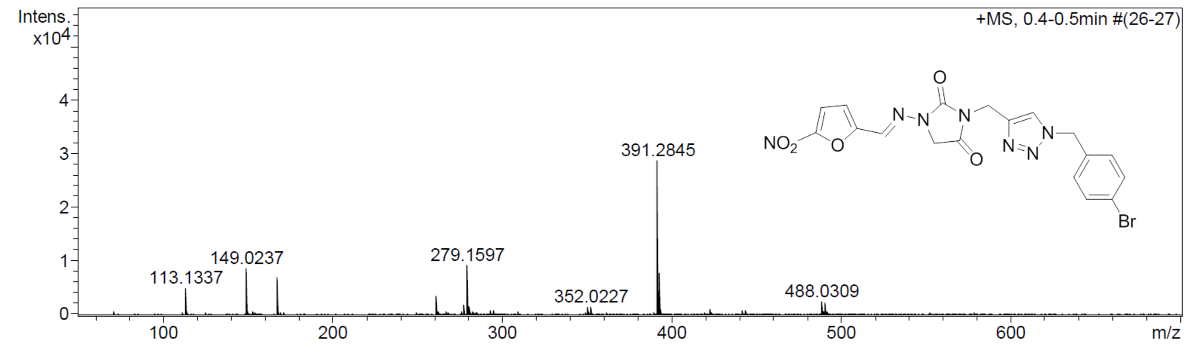

HPLC Purity

Analysis Report

SHIMADZU LabSolutions

<Sample Information>

Sample Name	: David NDa S15		
Sample ID	: David NDa S15		
Data Filename	: David NDa S15_018.lcd		
Method Filename	: PURITY non-polar.lcm		
Batch Filename	: David NDa.lcb		
Vial #	: 1-16	Sample Type	: Unknown
Injection Volume	: 1 uL		
Date Acquired	: 29/03/2022 12:20:26	Acquired by	: System Administrator
Date Processed	: 29/03/2022 12:33:27	Processed by	: System Administrator

<Chromatogram>

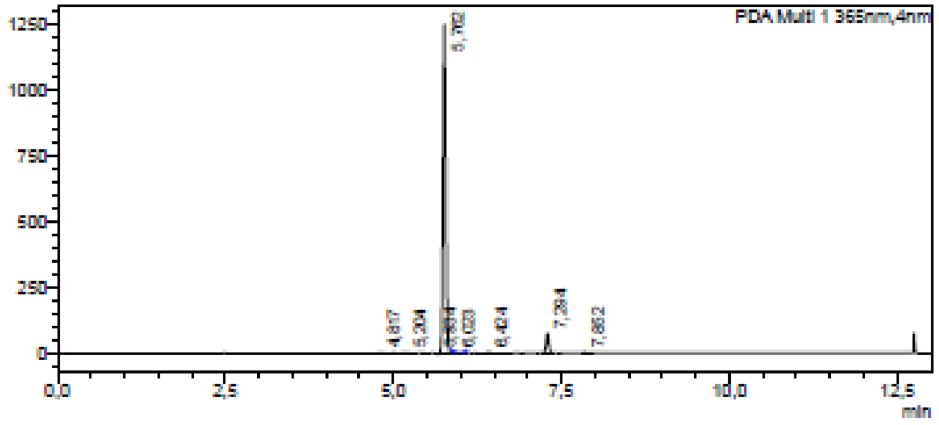

<Peak Table>

PDA Ch1 365nm

Peak#	Ret. Time	Area	Area%
1	4,817	13720	0,333
2	5,204	20946	0,509
3	5,762	3816040	92,739
4	5,934	16415	0,399
5	6,023	10744	0,261
6	6,424	15974	0,388
7	7,294	210946	5,127
8	7,852	10022	0,244
Total		4114807	100,000

C:\LabSolutions\Data\Project1\PURITY\David NDa S15_018.lcd

(E)-10-[(5-Bromo-7-fluorobenzyl-1H-1,2,3-triazolyl)methyl]-1-{[(5-nitrofuran-2-yl) methylene]amino}imidazolidine-9,11-dione, ***16***

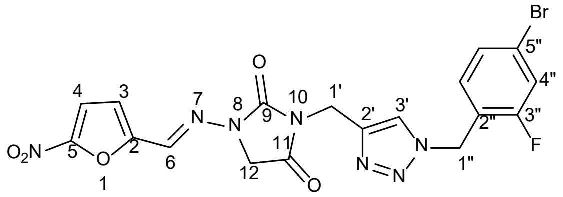

The reaction of **1** with 4-(azidomethyl)-1-bromo-2-fluorobenzene yielded **16** as a dark orange powder, 554 mg (60%), mp: 175.1-175.8 °C (EtOH/H_2O). IR (ATR) ν_{max}/cm^{-1}: 3135 (C-H, alkene), 1778 (C=O), 1720 (C=O), 1343 (C-N). ^{1}H NMR (600 MHz, DMSO) δ (ppm): 8.13 (s, 1H, H-6), 7.88 (s, 1H, H-3'), 7.80 (d, *J* = 3.9 Hz, 1H, H-4), 7.63 (d, *J* = 8.2, 1H, H-6"), 7.47 (dd, *J* = 8.2, 1.8 Hz, 1H, H-4"), 7.32 (d, *J* = 8.2 Hz, 1H, H-7"), 7.18 (d, *J* = 3.9 Hz, 1H, H-3), 5.63 (s, 2H, H-1"), 4.73 (s, 2H, H-1'), 4.47 (s, 2H, H-12). ^{13}C NMR (151 MHz, DMSO) δ (ppm): 167.3 (C-11), 161.3 (C-3"), 159.7 (C-9), 152.9 (C-2), 152.3 (C-5), 142.3 (C-2"), 132.8 (C-7"), 132.4 (C-6), 128.6 (C-6"), 124.3 (C-3'), 122.8 (C-5"), 115.3 (C-4), 115.2 (C-3), 48.8 (C-1"), 46.7 (C-12). Purity: 93%. HRMS *m/z* [M+H]$^{+}$: 506.0239 (calcd. for $C_{18}H_{14}BrFN_7O_5^{+}$, 506.0224).

IR

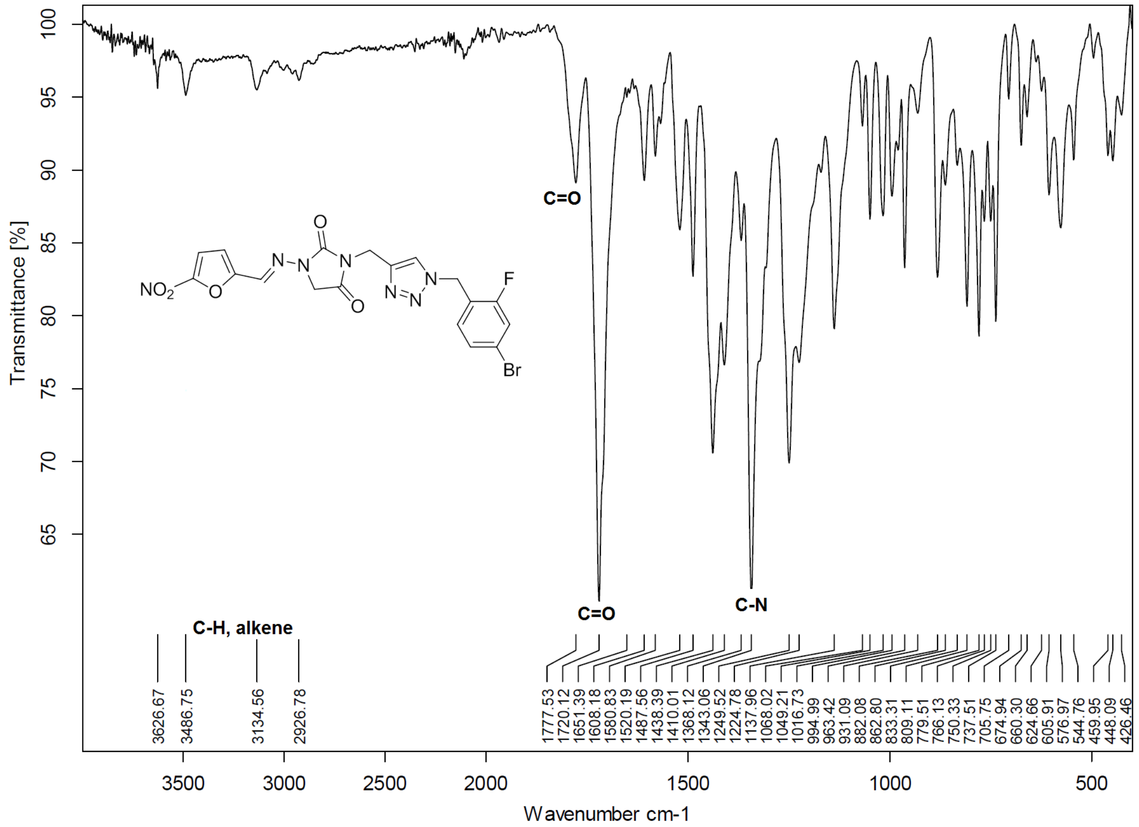

^{1}H-NMR

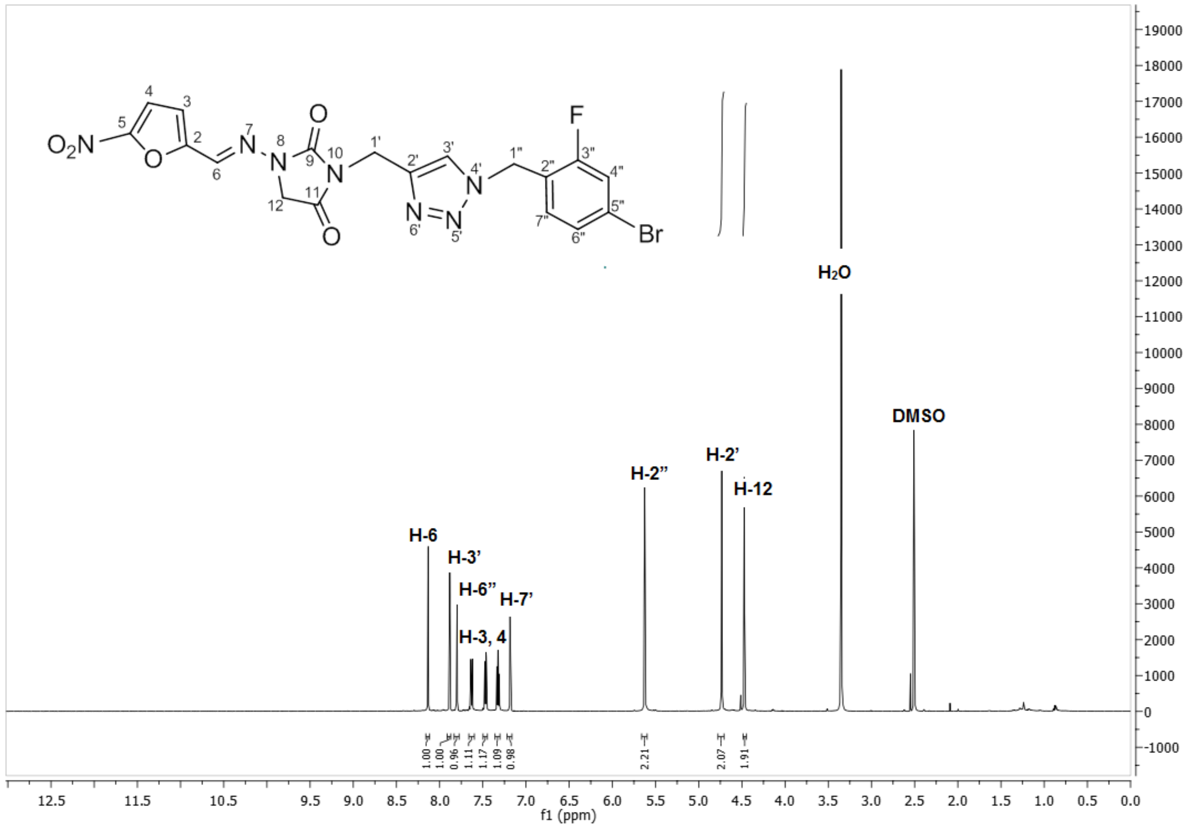

^{13}C-NMR

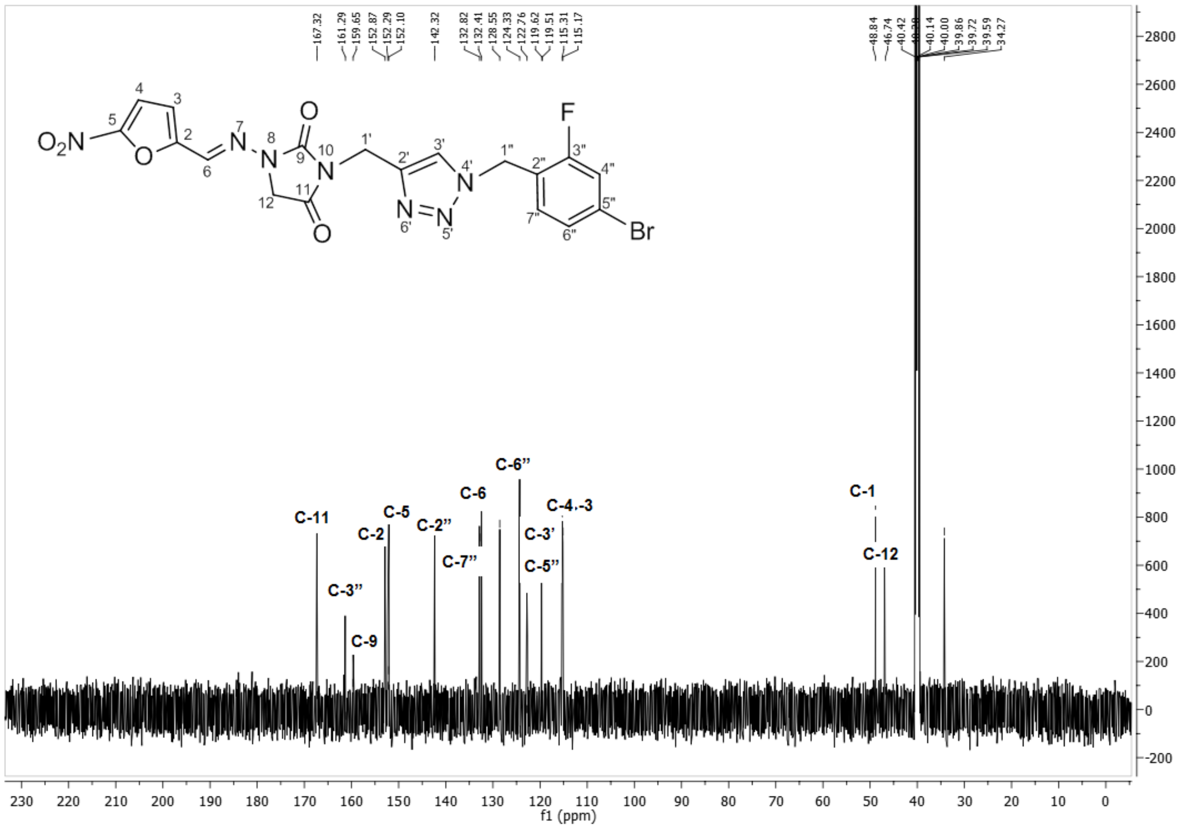

HRMS

Acquisition Parameter

Source Type	APCI	Ion Polarity	Positive	Set Nebulizer	1.6 Bar
Focus	Not active	Set Capillary	4500 V	Set Dry Heater	200 °C
Scan Begin	50 m/z	Set End Plate Offset	-500 V	Set Dry Gas	8.0 l/min
Scan End	1500 m/z	Set Collision Cell RF	100.0 Vpp	Set Divert Valve	Waste

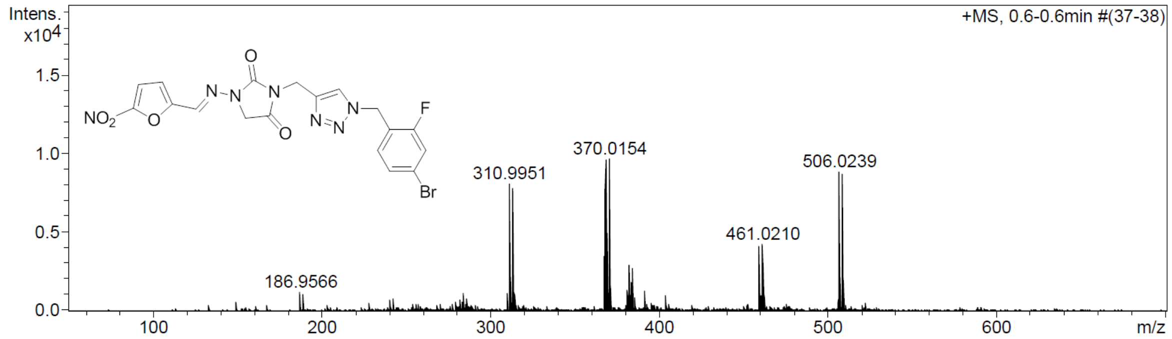

HPLC Purity

SHIMADZU LabSolutions Analysis Report

<Sample Information>

Sample Name	: David NDa S16		
Sample ID	: David NDa S16		
Data Filename	: David NDa S16_019.lcd		
Method Filename	: PURITY non-polar.lcm		
Batch Filename	: David NDa.lcb		
Vial #	: 1-17	Sample Type	: Unknown
Injection Volume	: 1 uL		
Date Acquired	: 29/03/2022 12:33:48	Acquired by	: System Administrator
Date Processed	: 29/03/2022 12:46:50	Processed by	: System Administrator

<Chromatogram>

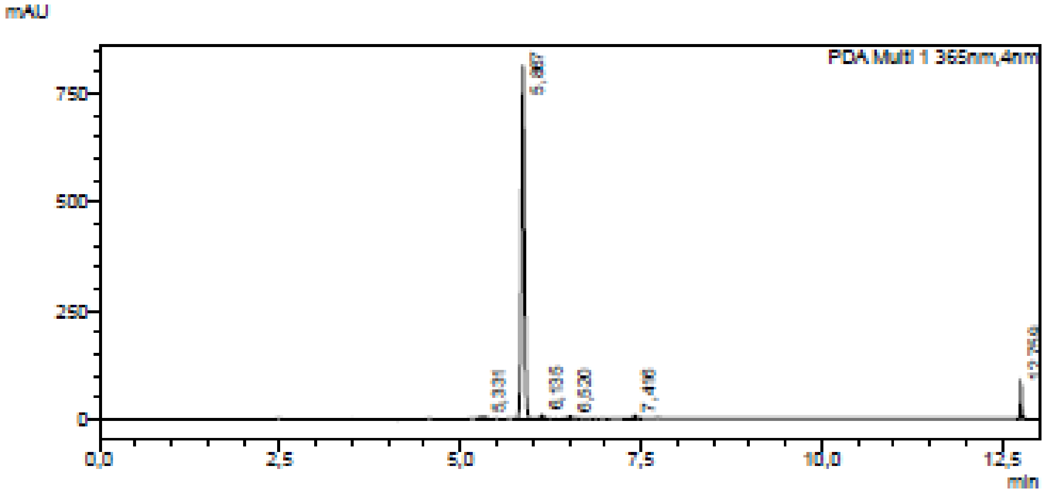

<Peak Table>

PDA Ch1 365nm

Peak#	Ret. Time	Area	Area%
1	5,331	33463	1,262
2	5,867	2466026	93,013
3	6,135	29885	1,127
4	6,520	21182	0,799
5	7,416	22792	0,860
6	12,759	77933	2,939
Total		2651281	100,000

C:\LabSolutions\Data\Project1\PURITY\David NDa S16_019.lcd

(E)-10-[(5-Fluorobenzyl-1H-1,2,3-triazolyl)methyl]-1-{[(5-nitrofuran-2-yl)methylene] amino}imidazolidine-9,11-dione, ***17***

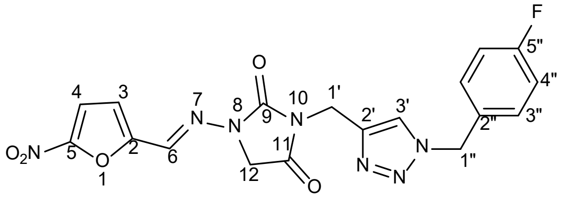

The reaction of **1** with 1-(azidomethyl)-4-fluorobenzene yielded **17** as a dark orange powder 374 mg (48%), mp: 184.4-185.4 °C (EtOH/H_2O). IR (ATR) ν_{max}/cm^{-1}: 2921 (C-H, alkane), 1783 (C=O), 1713 (C=O), 1343 (C-N). ^{1}H NMR (600 MHz, DMSO) δ (ppm): 8.15 (s, 1H, H-6), 7.88 (s, 1H, H-3'), 7.80 (d, *J* = 3.9 Hz, 1H, H-4), 7.40 (dd, *J* = 8.7, 5.5 Hz, 2H, H-3"), 7.21 (t, *J* = 8.9 Hz, 2H, H-4",), 7.18 (d, *J* = 3.9 Hz, 1H, H-3), 5.57 (s, 2H, H-1"), 4.73 (s, 2H, H-1'), 4.46 (s, 2H, H-12). ^{13}C NMR (151 MHz, DMSO) δ (ppm): 167.4 (C-11), 161.6 (C-5"), 152.9 (C-9), 152.3 (C-2), 152.1 (C-5), 142.4 (C-2"), 132.4 (C-2'), 130.9 (C-3"), 130.8 (C-4"), 124.1 (C-3'), 116.2 (C-5"), 115.3 (C-4), 115.2 (C-3), 52.5 (C-1"), 48.8 (C-12). Purity: 92%. HRMS *m/z* $[M+H]^+$: 428.1113 (calcd. for $C_{18}H_{15}FN_7O_5^+$, 428.1119).

IR

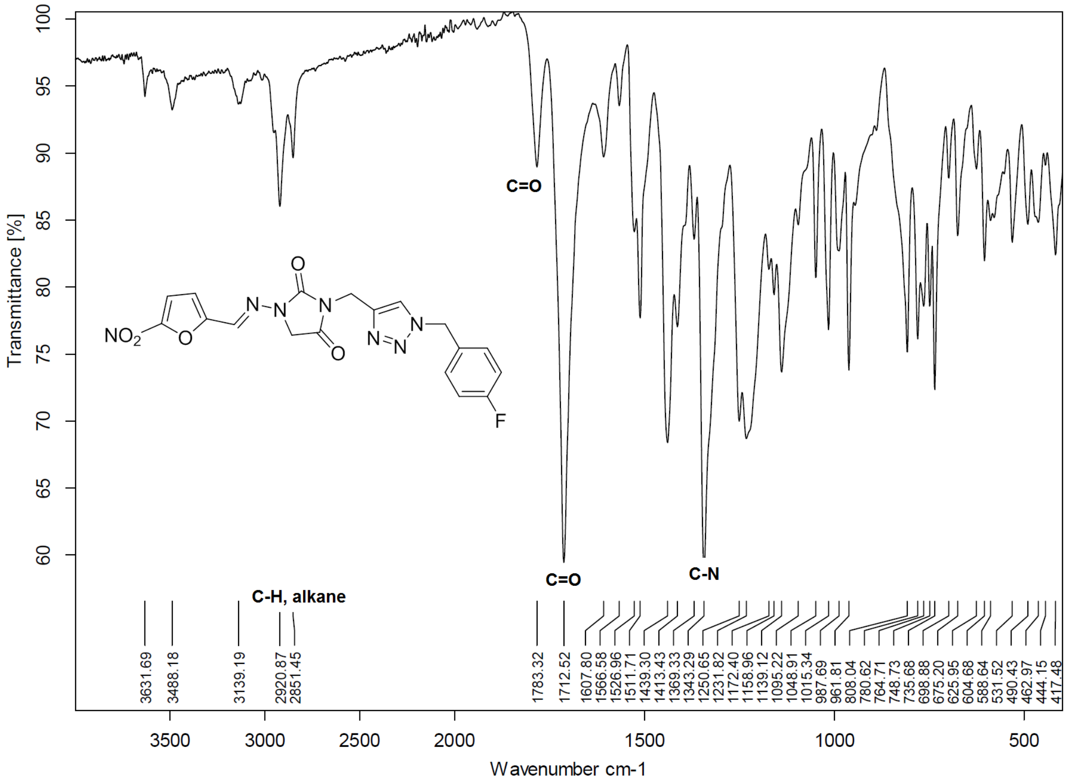

^{1}H-NMR

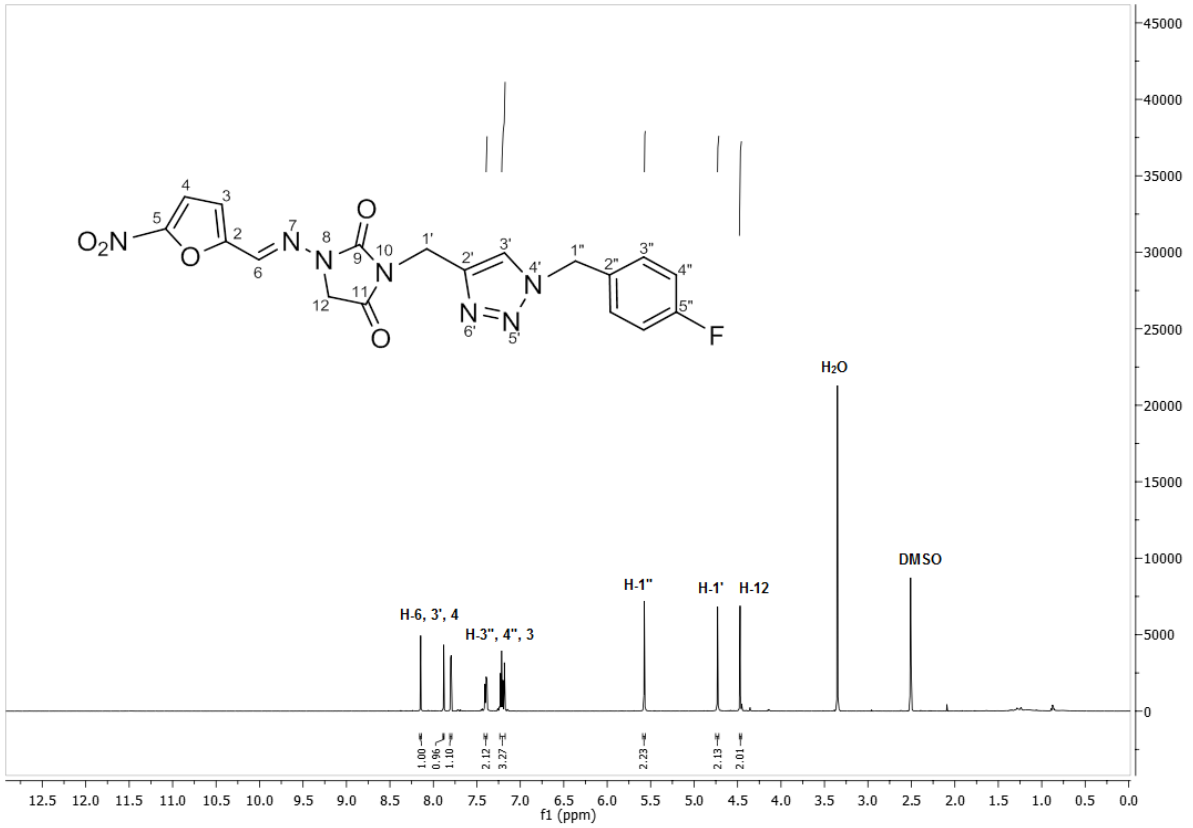

^{13}C-NMR

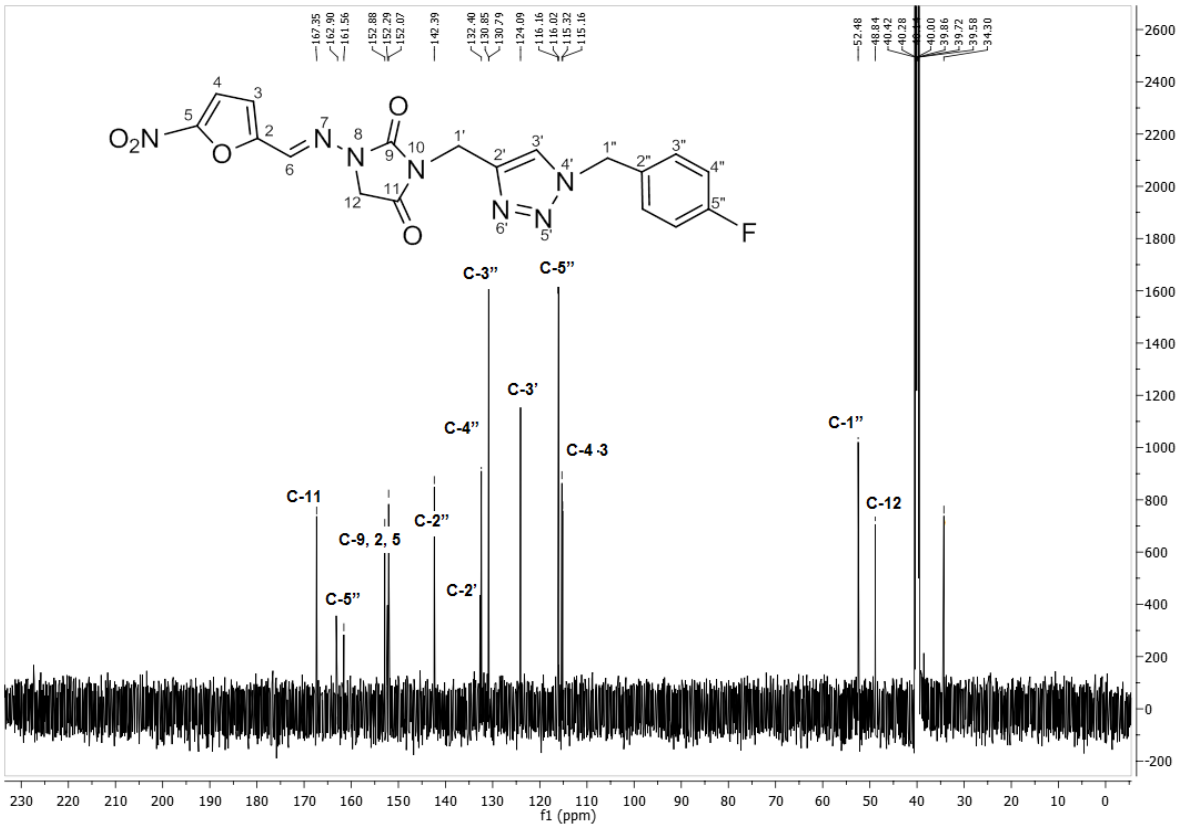

HRMS

Acquisition Parameter

Source Type	APCI	Ion Polarity	Positive	Set Nebulizer	1.6 Bar
Focus	Not active	Set Capillary	4500 V	Set Dry Heater	200 °C
Scan Begin	50 m/z	Set End Plate Offset	-500 V	Set Dry Gas	8.0 l/min
Scan End	1500 m/z	Set Collision Cell RF	100.0 Vpp	Set Divert Valve	Waste

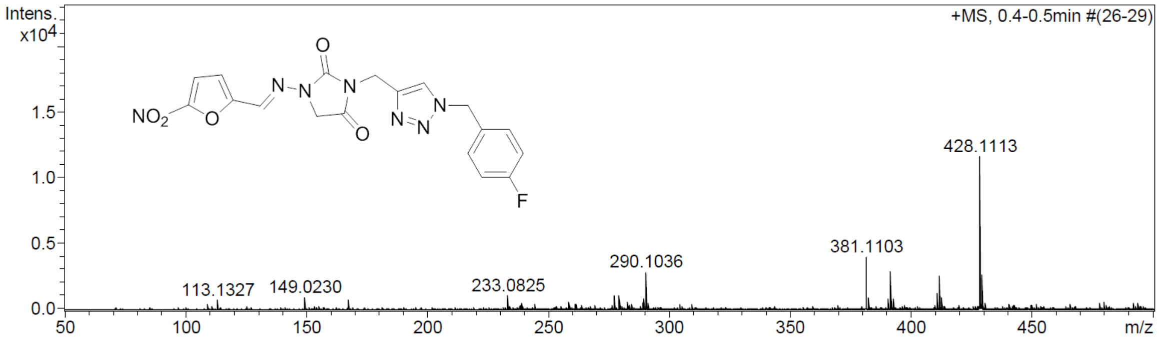

HPLC Purity

SHIMADZU LabSolutions

Analysis Report

<Sample Information>

Sample Name	: David NDa S17		
Sample ID	: David NDa S17		
Data Filename	: David NDa S17_020.lcd		
Method Filename	: PURITY non-polar.lcm		
Batch Filename	: David NDa.lcb		
Vial #	: 1-18	Sample Type	: Unknown
Injection Volume	: 1 uL		
Date Acquired	: 29/03/2022 12:47:10	Acquired by	: System Administrator
Date Processed	: 29/03/2022 13:00:11	Processed by	: System Administrator

<Chromatogram>

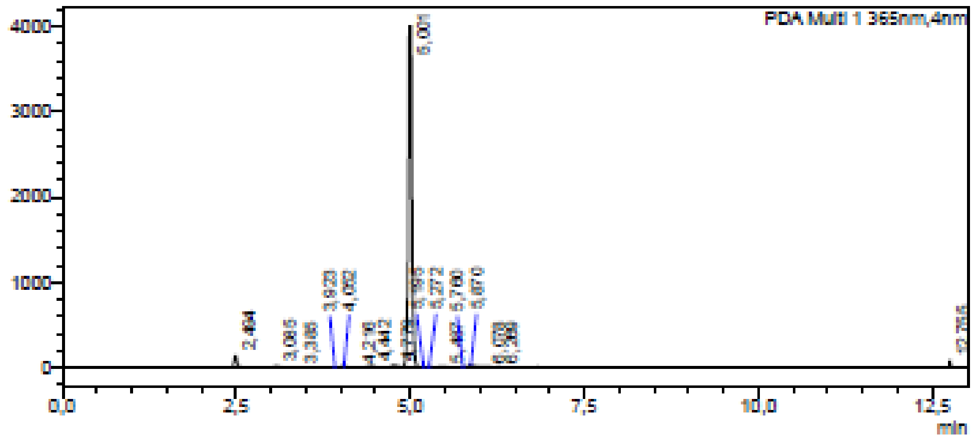

<Peak Table>

PDA Ch1 365nm

Peak#	Ret. Time	Area	Area%
1	2,494	419486	2,620
2	3,085	52571	0,328
3	3,385	21283	0,133
4	3,923	15838	0,099
5	4,052	77401	0,483
6	4,216	26829	0,168
7	4,442	134236	0,838
8	4,779	124742	0,779
9	5,001	14758706	92,170
10	5,195	20165	0,126
11	5,272	17984	0,112
12	5,487	39026	0,244
13	5,760	15374	0,096
14	5,870	170236	1,063
15	6,078	25241	0,158
16	6,269	16789	0,105
17	12,755	76653	0,479
Total		16012559	100,000

C:\LabSolutions\Data\Project1\PURITY\David NDa S17_020.lcd

(E)-10-[(5-Nitrobenzyl-1H-1,2,3-triazolyl)methyl]-1-{[(5-nitrofuran-2-yl)methylene]amino}imidazolidine-9,11-dione, ***18***

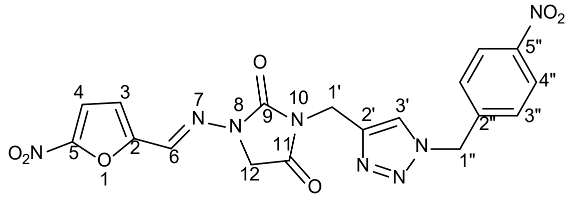

The reaction of **1** with 1-(azidomethyl)-4-nitrobenzene yielded **18** as a dark yellow powder, 249 mg (30%), mp: 181.2-181.8 °C (EtOH/H_2O). IR (ATR) ν_{max}/cm^{-1}: 2918 (C-H, alkane), 1785 (C=O), 1729 (C=O), 1340 (C-N). ^{1}H NMR (600 MHz, DMSO) δ (ppm): 8.39 (s, 1H, H-6), 8.37 (s, 1H, H-3'), 8.30 (d, *J* = 8.6 Hz, 2H, H-4"), 7.83 (d, *J* = 8.6 Hz, 2H, 3"), 7.80 (d, *J* = 3.9 Hz, 1H, H-4), 7.19 (d, *J* = 3.9 Hz, 1H, H-3), 5.57 (s, 2H, H-1"), 4.73 (s, 2H, H-1'), 4.29 (s, 2H, H-12). ^{13}C NMR (151 MHz, DMSO) δ (ppm): 166.9 (C-11), 152.3 (C-9), 152.00 (C-2), 144.8 (C-5"), 142.6 (C-2"), 132.6 (C-6), 126.8 (C-3"), 124.8 (C-4"), 124.6 (C-3'), 115.5 (C-4), 115.1 (C-3), 57.28 (C-1"), 48.6 (C-12). Purity: not determined. HRMS *m/z* [M+H]$^+$: 455.1000 (calcd. for $C_{18}H_{15}N_8O_7^+$, 455.1064).

IR

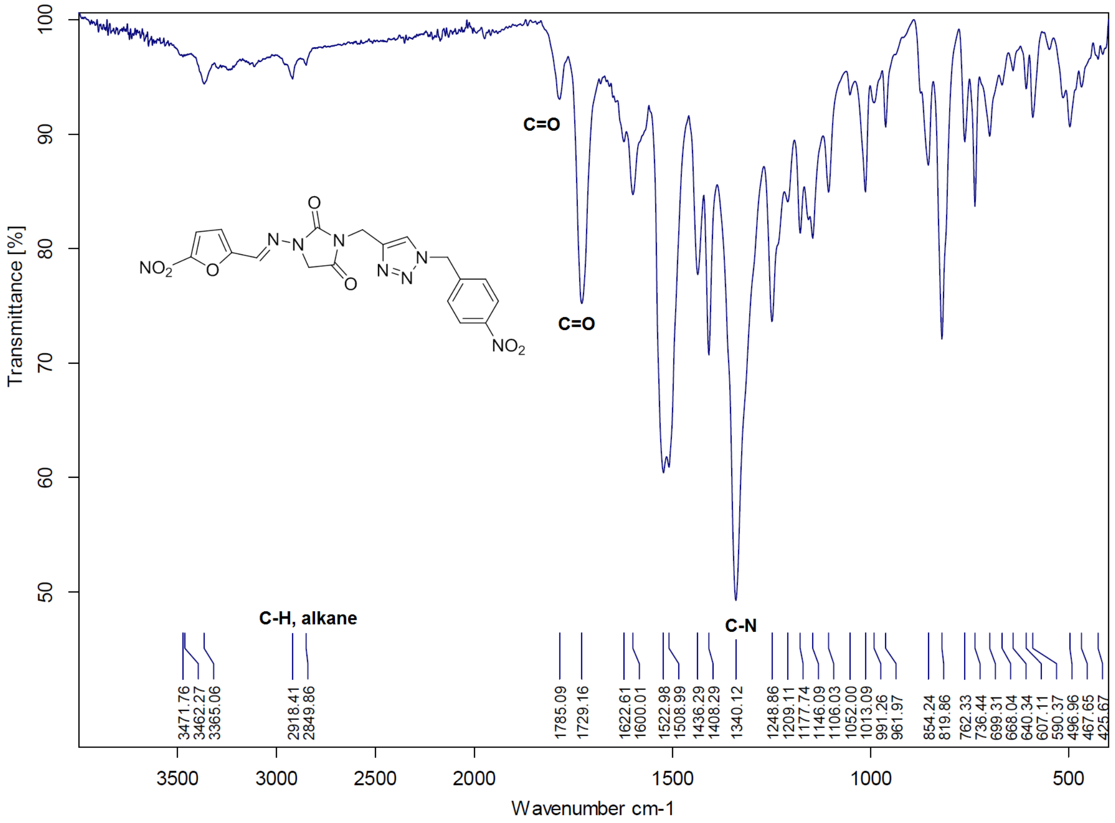

^{1}H-NMR

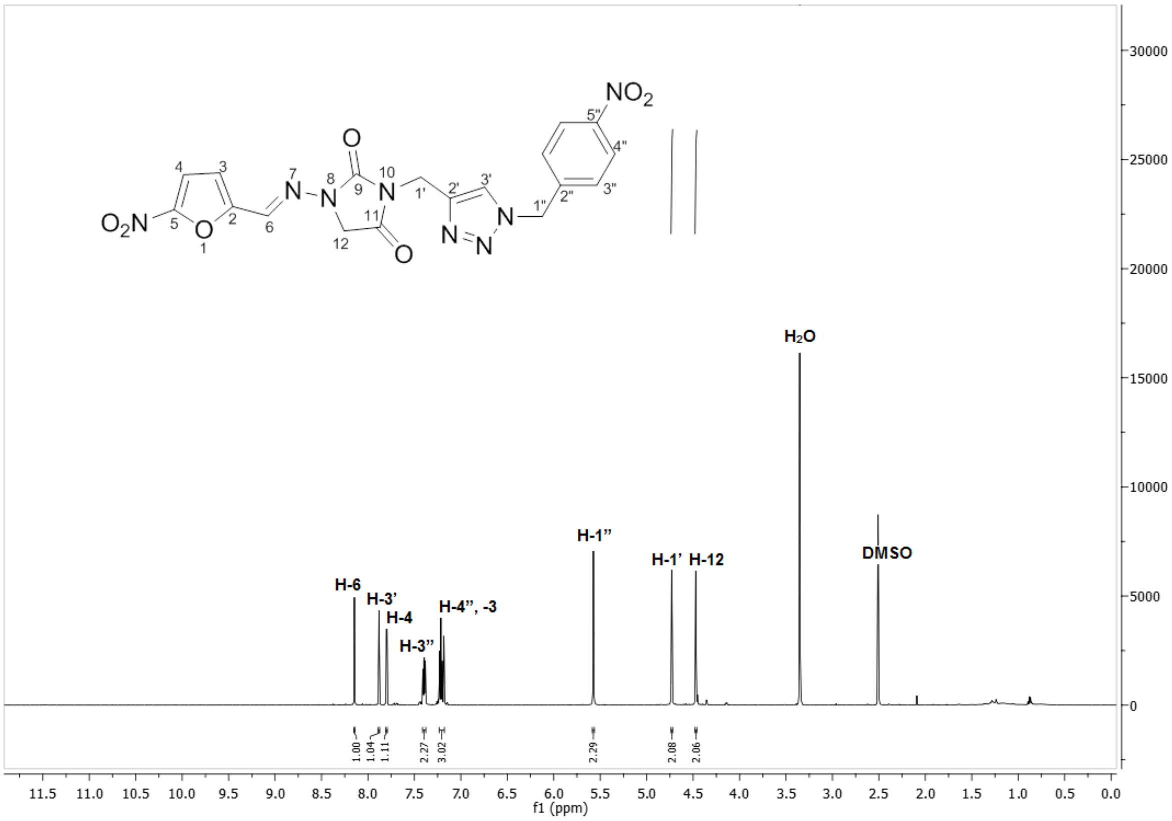

^{13}C-NMR

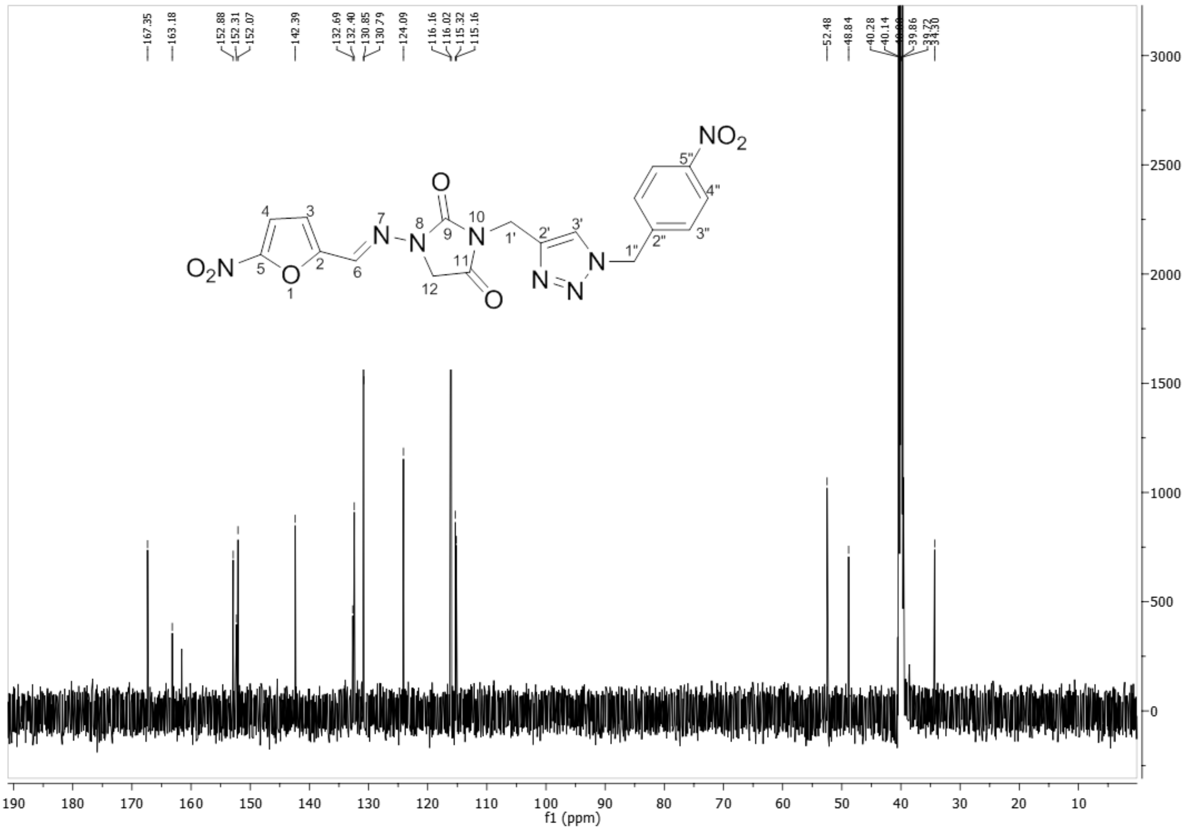

HRMS

Acquisition Parameter

Source Type	APCI	Ion Polarity	Positive	Set Nebulizer	1.6 Bar
Focus	Not active	Set Capillary	4500 V	Set Dry Heater	200 °C
Scan Begin	50 m/z	Set End Plate Offset	-500 V	Set Dry Gas	8.0 l/min
Scan End	1500 m/z	Set Collision Cell RF	100.0 Vpp	Set Divert Valve	Waste

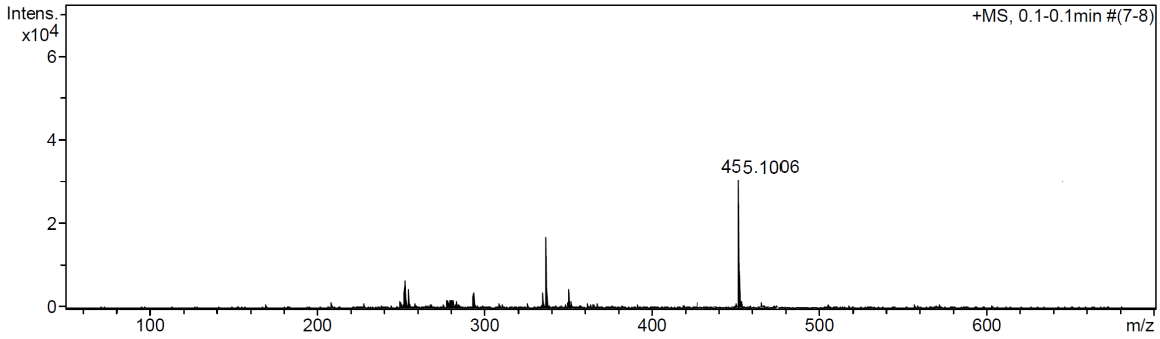

(E)-10-[(4-Bromobenzyl-1H-1,2,3-triazolyl)methyl]-1-{[(5-nitrofuran-2-yl)methylene]amino}imidazolidine-9,11-dione, ***19***

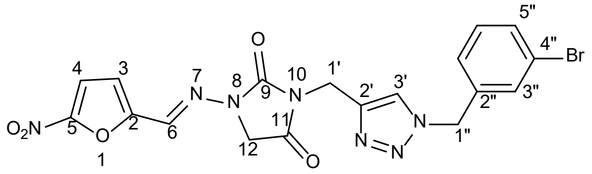

The reaction of **1** with 1-(azidomethyl)-3-bromobenzene yielded **20** as a fine dark orange powder, 546 mg (62%), mp: 188.0-188.7 °C (EtOH/H_2O). IR (ATR) $\tilde{\nu}_{max}$/cm^{-1}: 3134 (C-H, alkene), 2924 (C-H, alkane), 1783 (C=O), 1715 (C=O), 1343 (C-N). ^{1}H NMR (600 MHz, DMSO) δ (ppm): 8.19 (s, 1H, H-6), 7.88 (s, 1H, H-3'), 7.80 (d, *J* = 3.9 Hz, 1H, H-4), 7.58 – 7.53 (m, 2H, H-5", -3"), 7.37 – 7.29 (m, 2H, H-6", 7"), 7.18 (d, *J* = 3.9 Hz, 1H, H-3), 5.59 (s, 2H, H-1"), 4.74 (s, 2H, H-1'), 4.48 (s, 2H, H-12). ^{13}C NMR (151 MHz, DMSO) δ (ppm): 167.4 (C-11), 152.9 (C-9), 152.1 (C-2, -5), 142.4 (C-2"), 139.0 (C-3"), 131.6 (C-6), 131.5 (C-6"), 131.3 (C-5"), 127.6 (C-7"), 124.4 (C-3'), 122.3 (C-4"), 115.3 (C-4), 115.2 (C-3), 52.4 (C-1"), 48.9 (C-12), Purity 93%. HRMS *m/z* [M+H]$^+$: 490.0334 (calcd. for $C_{19}H_{15}BrN_7O_5^+$, 490.0298).

IR

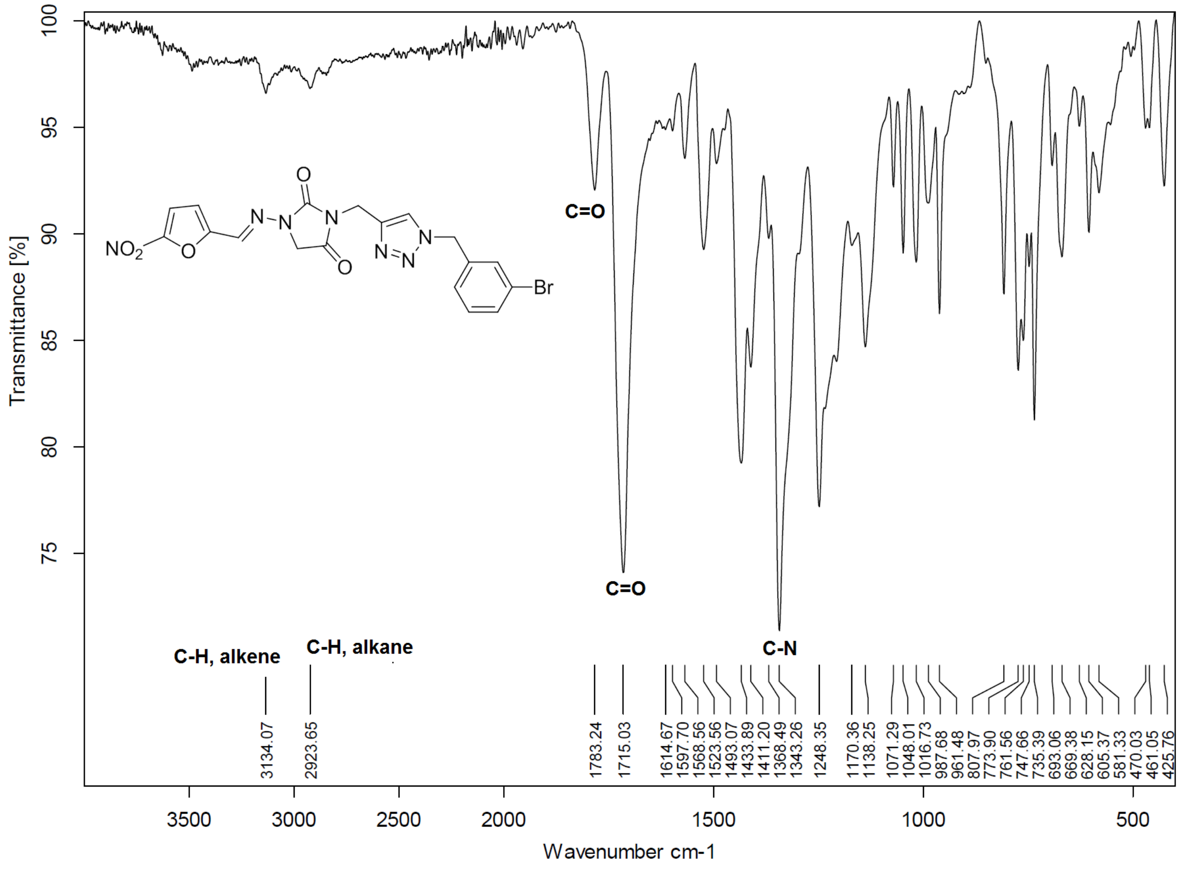

^{1}H-NMR

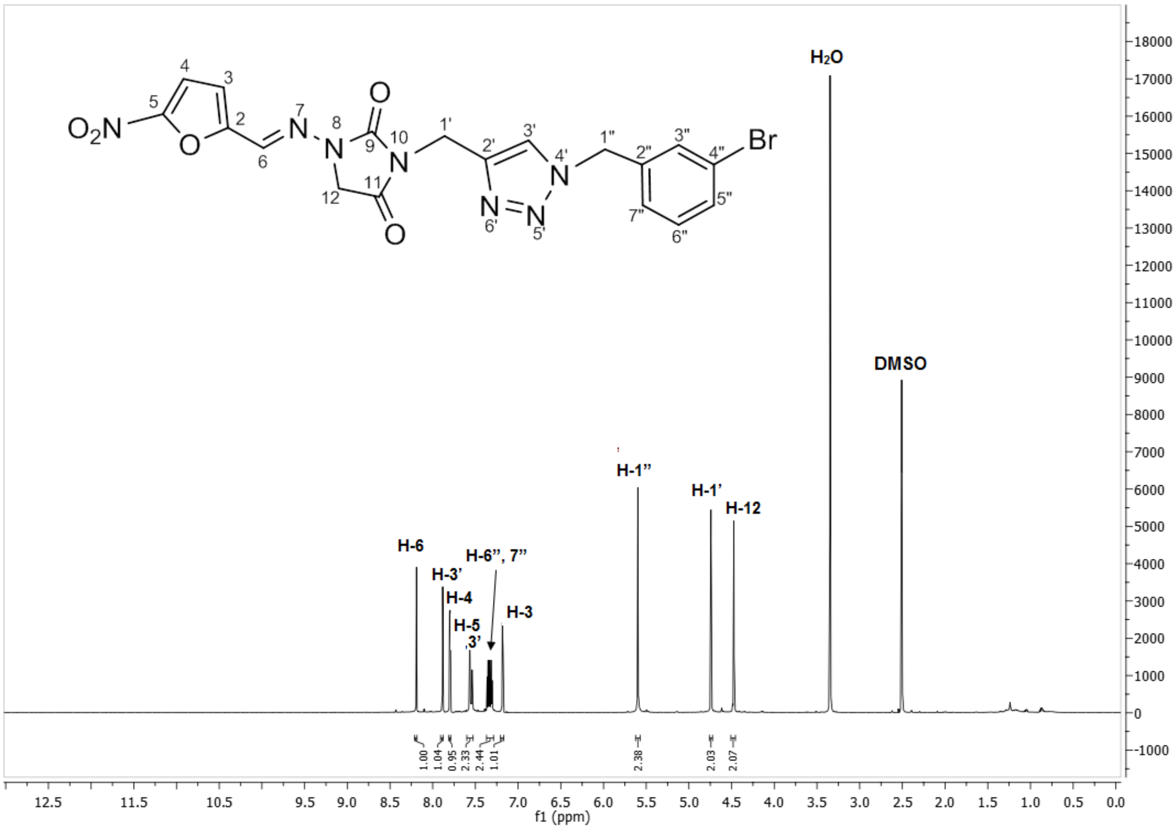

^{13}C-NMR

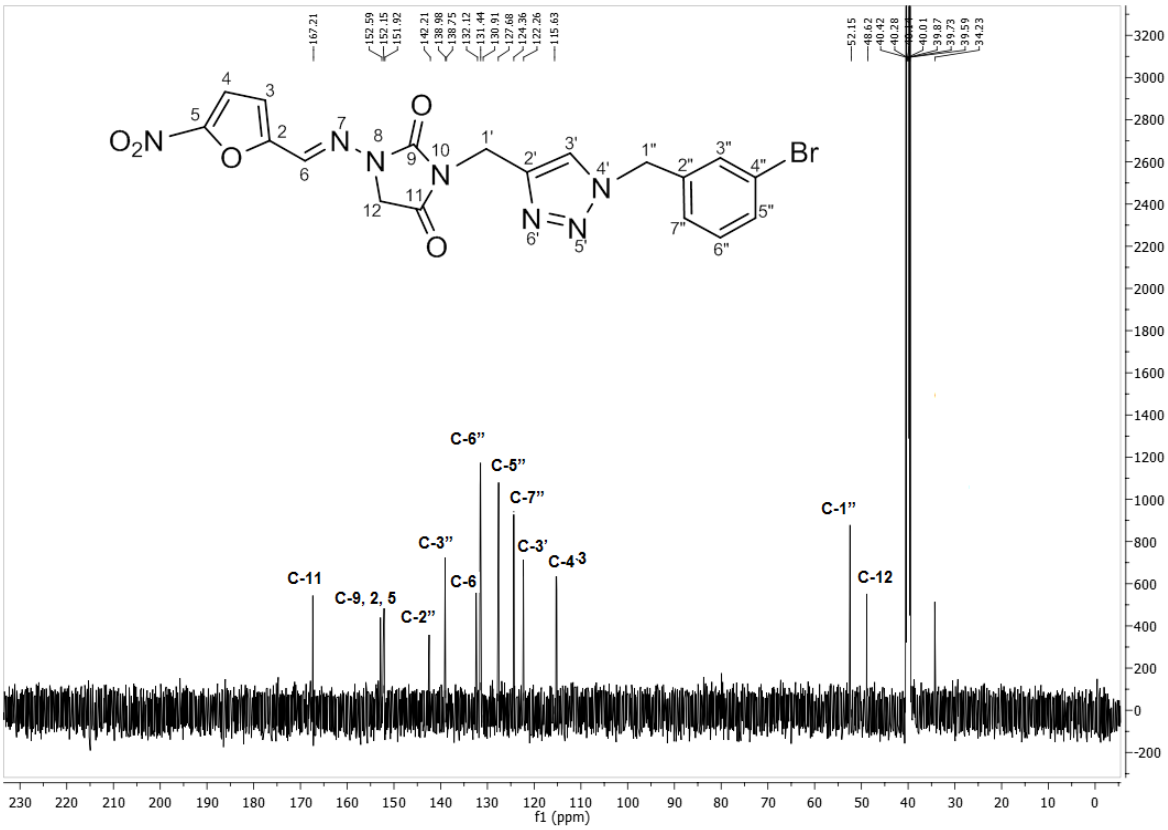

HRMS

Acquisition Parameter

Source Type	APCI	Ion Polarity	Positive	Set Nebulizer	1.6 Bar
Focus	Not active	Set Capillary	4500 V	Set Dry Heater	200 °C
Scan Begin	50 m/z	Set End Plate Offset	-500 V	Set Dry Gas	8.0 l/min
Scan End	1600 m/z	Set Collision Cell RF	80.0 Vpp	Set Divert Valve	Waste

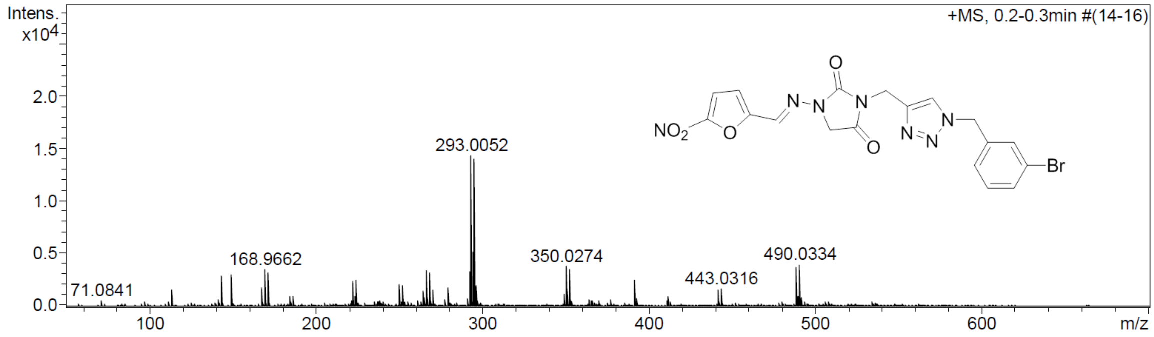

HPLC Purity

2022/03/31 08:57:19 Page 1 / 1

Analysis Report

<Sample Information>

Sample Name	: David NDa S19		
Sample ID	: David NDa S19		
Data Filename	: David NDa S19_022.lcd		
Method Filename	: PURITY non-polar.lcm		
Batch Filename	: David NDa.lcb		
Vial #	: 1-20	Sample Type	: Unknown
Injection Volume	: 1 uL		
Date Acquired	: 29/03/2022 13:13:52	Acquired by	: System Administrator
Date Processed	: 29/03/2022 13:26:54	Processed by	: System Administrator

<Chromatogram>

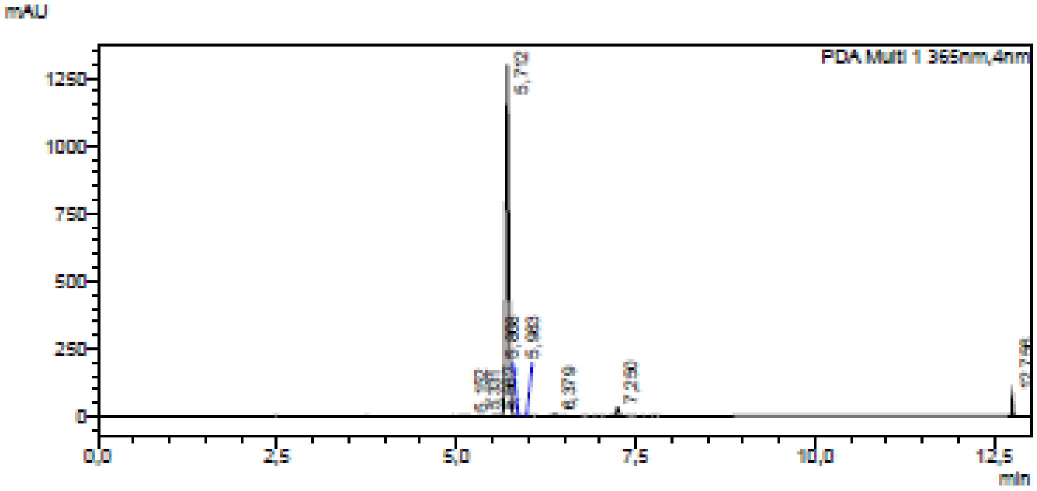

<Peak Table>

PDA Ch1 365nm

Peak#	Ret. Time	Area	Area%
1	5,152	25157	0,592
2	5,331	15694	0,369
3	5,562	20367	0,479
4	5,712	3963568	93,209
5	5,868	14398	0,339
6	5,983	13917	0,327
7	6,379	30419	0,715
8	7,250	91758	2,158
9	12,756	77080	1,813
Total		4252357	100,000

C:\LabSolutions\Data\Project1\PURITY\David NDa S19_022.lcd

(E)-10-[(4-Trifluoromethylbenzyl-1H-1,2,3-triazolyl)methyl]-1-{[(5-nitrofuran-2-yl) methylene]amino}imidazolidine-9,11-dione, ***20***

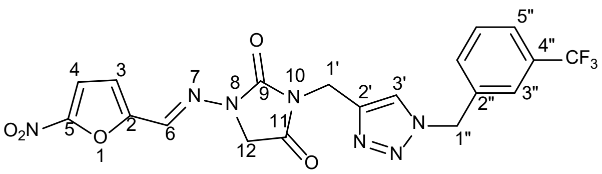

The reaction of **1** with 1-(azidomethyl)-3-(trifluoromethyl)benzene yielded **19** as a dark orange powder, 453 mg (52%), mp: 185.4-186.2 °C (EtOH/H_2O). IR (ATR) ν_{max}/cm^{-1}: 3133 (C-H, alkene), 2924 (C-H, alkane), 1785 (C=O), 1721 (C=O), 1326 (C-N). ^1H NMR (600 MHz, DMSO) δ (ppm): 8.23 (s, 1H, H-6), 7.88 (s, 1H, H-3'), 7.80 (d, *J* = 3.9 Hz, 1H, H-4), 7.73 – 7.62 (m, 4H, H-3", 6", 5", 7"), 7.19 (d, *J* = 3.9 Hz, 1H, H-3), 5.71 (s, 2H, H-1"), 4.75 (s, 2H, H-1'), 4.48 (s, 2H, H-12). ^{13}C NMR (151 MHz, DMSO) δ (ppm): 167.3 (C-11), 161.3 (C-3"), 159.7 (C-9), 152.9 (C-2), 152.3 (C-5), 142.3 (C-2"), 132.8 (C-7"), 132.4 (C-6), 128.6 (C-6"), 124.3 (C-3'), 122.8 (C-5"), 115.3 (C-4), 115.2 (C-3), 48.8 (C-1"), 46.7 (C-12). Purity: 85%. HRMS *m/z* $[M+H]^+$: 478.1034 (calcd. for $C_{19}H_{15}FN_7O_5^+$, 478.1087).

IR

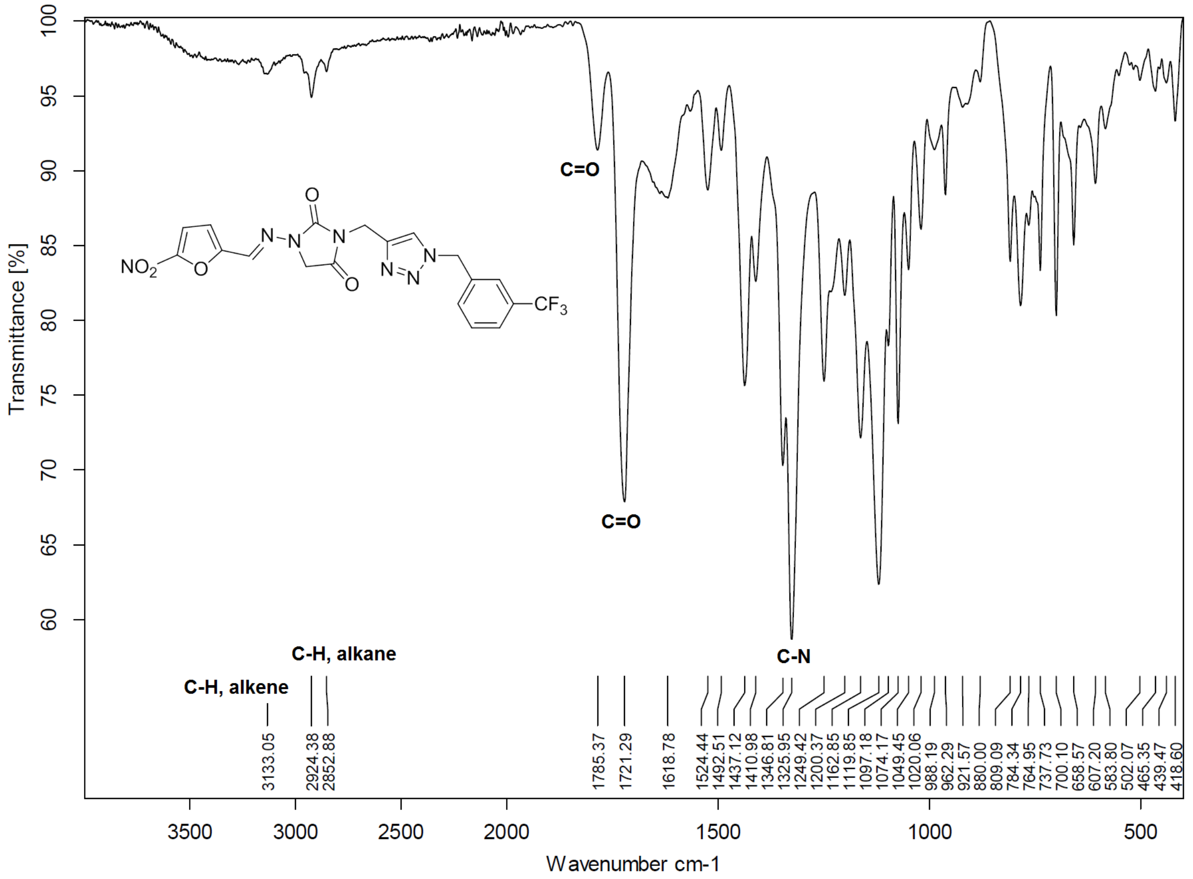

^{1}H-NMR

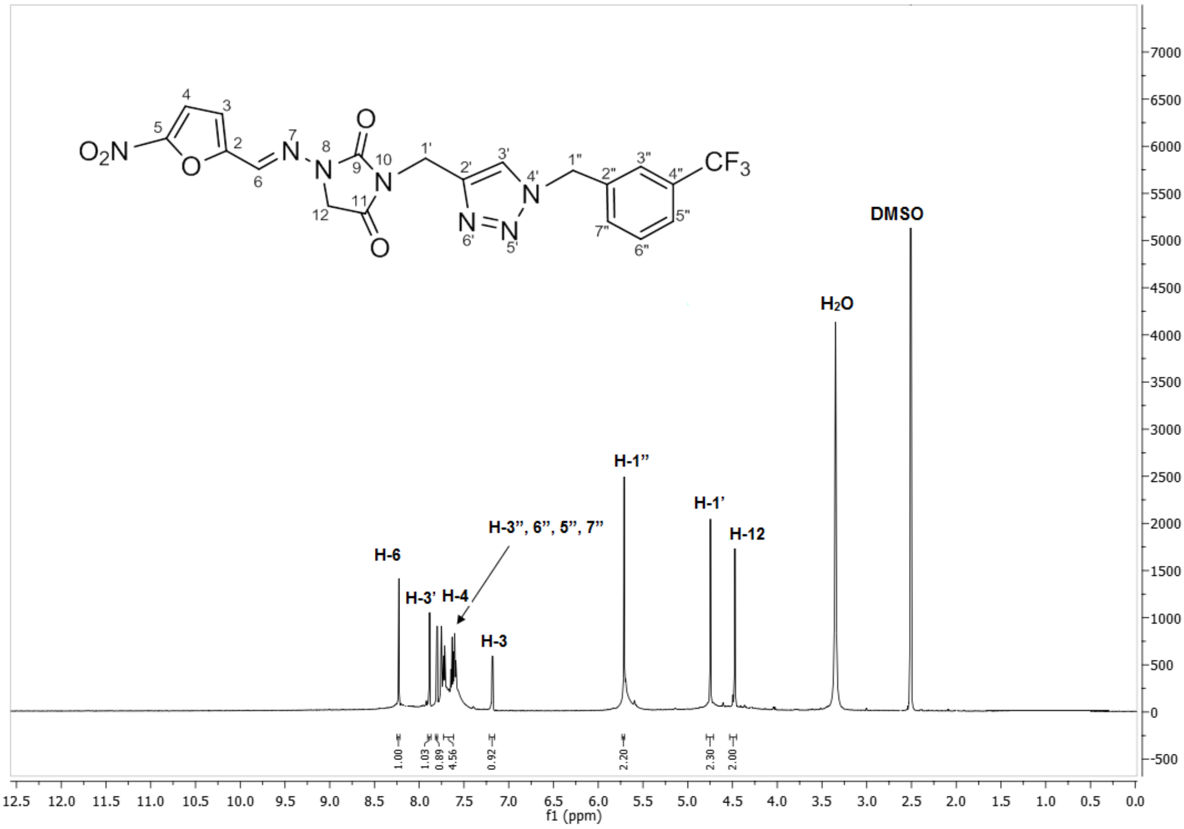

^{13}C-NMR

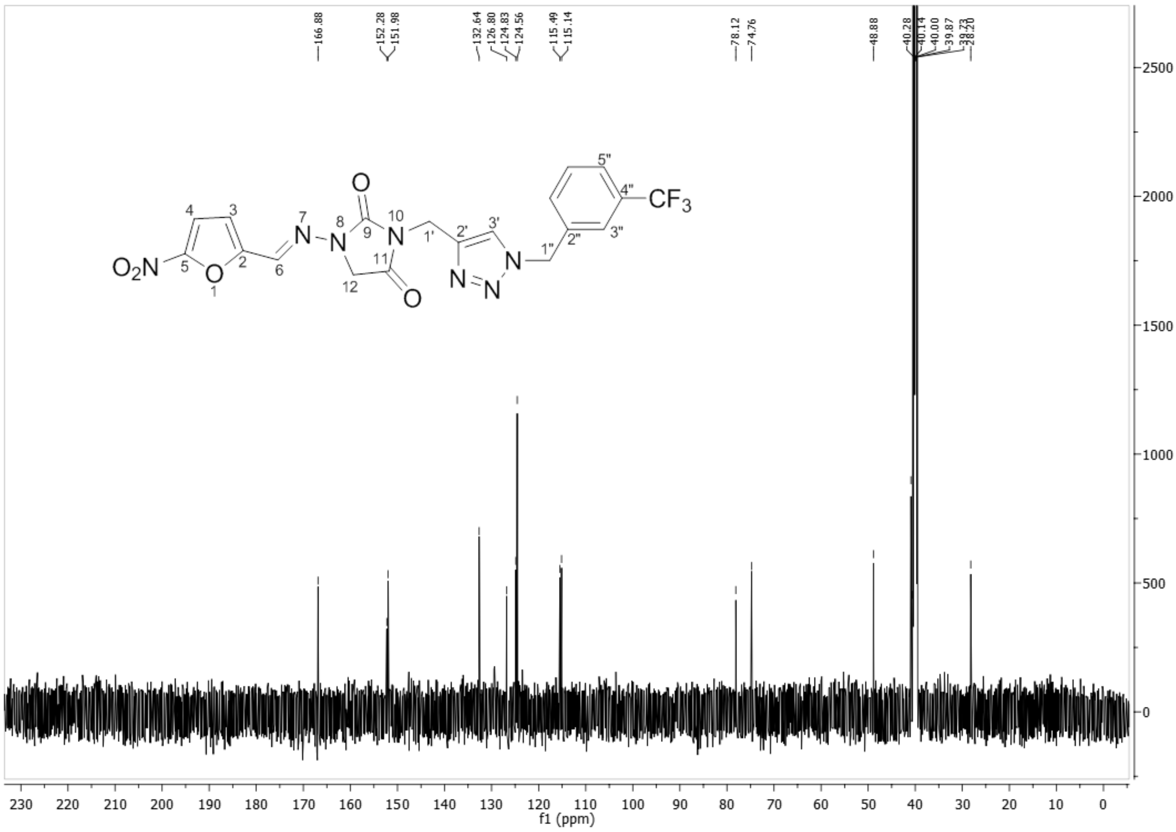

HRMS

Acquisition Parameter

Source Type	APCI	Ion Polarity	Positive	Set Nebulizer	1.6 Bar
Focus	Not active	Set Capillary	4500 V	Set Dry Heater	200 °C
Scan Begin	50 m/z	Set End Plate Offset	-500 V	Set Dry Gas	8.0 l/min
Scan End	1500 m/z	Set Collision Cell RF	100.0 Vpp	Set Divert Valve	Waste

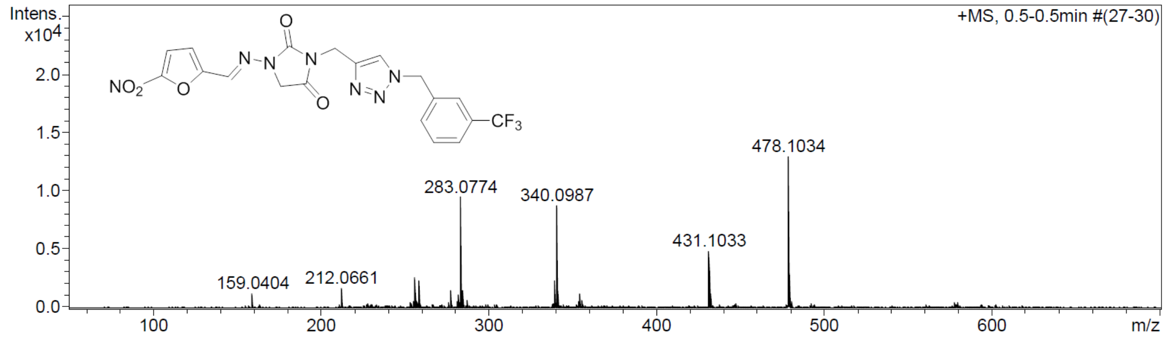

HPLC Purity

Analysis Report

<Sample Information>

Sample Name	: David NDa S20		
Sample ID	: David NDa S20		
Data Filename	: David NDa S20_023.lcd		
Method Filename	: PURITY non-polar.lcm		
Batch Filename	: David NDa.lcb		
Vial #	: 1-21	Sample Type	: Unknown
Injection Volume	: 1 uL		
Date Acquired	: 29/03/2022 13:27:13	Acquired by	: System Administrator
Date Processed	: 29/03/2022 13:40:14	Processed by	: System Administrator

<Chromatogram>

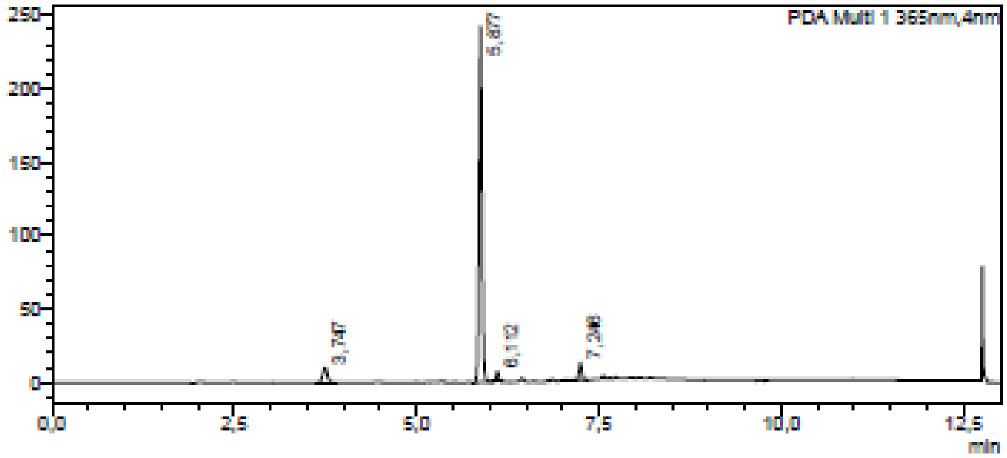

<Peak Table>

PDA Ch1 365nm

Peak#	Ret. Time	Area	Area%
1	3,747	43156	5,344
2	5,877	714554	88,485
3	6,112	18816	2,330
4	7,246	31018	3,841
Total		807545	100,000

In vitro biological assays

Antipromastigote assay

The antipromastigote activity of synthesized compounds was evaluated using a modified, resazurin-based method of Kulshrestha *et al.* [1] and Siqueira-Neto *et al.* [2] and on three *Leishmania* strains. *Leishmania donovani* (strains 1S (MHOM/SD/62/1S) and 9515 (MHOM/IN/95/9515)) and *L. major* (strain IR-173 (MHOM/IR/-173)) promastigotes were cultured in M199 with Hank's salts and 0.68 mM L-glutamine (Sigma Aldrich) supplemented with 50 U/mL Penicillin/Streptomycin solution (Lonza), 4.2 mM sodium bicarbonate, 25 mM Hepes, 10% fetal bovine serum, 0.25% hemin, 0.1% biotin, and 10 mM adenine (Sigma Aldrich) and the pH adjusted to 7.3 – 7.4. The promastigotes were maintained at 25 °C.

For the resazurin assay, logarithmic phase promastigotes (1.25 x 10^6 cells/mL, final volume 100 µL/well) were seeded in 96 well plates (Nunc, Thermofisher Scientific) in the presence of: (i) 10 µM of compound for activity screening or (ii) 7 two-fold dilution concentrations of 10 µM compounds for IC_{50} determination. Amphothericin B (10 µM) served as the standard drug and growth medium without parasites served as the blank. The plates were incubated for 48 hours at 25 ˚C in humidified atmosphere. After incubation, 50 µL of resazurin solution (0.01% in PBS) was added to each well and the plates were further incubated at 25 ˚C in the dark for 24 hours. Absorbance was measured at 570 nm and 600 nm using the Thermofisher Scientific GO Multiscan plate reader. Data analysis was performed for each biological replicate using SkanIt 4.0 Research Edition software. Background absorbance of resazurin (600 nm) was subtracted from the absorbance values of resorufin (570 nm). The mean absorbance calculated and the percentage growth inhibition and cell viability were determined by the following equations:

$$\text{Growth inhibition \%} = \frac{(\Delta\ \text{Abs neg control} - \Delta\ \text{Abs blank}) - (\Delta\ \text{Abs sample} - \Delta\ \text{Abs blank})}{(\Delta\ \text{Abs neg control} - \Delta\ \text{Abs blank})} \times 100$$

$$\text{Cell viability \%} = \frac{(\Delta\ \text{Abs sample} - \Delta\ \text{Abs blank})}{(\Delta\ \text{Abs neg control} - \Delta\ \text{Abs blank})} \times 100$$

All compounds were first screened for >70% growth inhibition at 10 µM [2] and qualifying compounds were further used for IC_{50} determinations. The IC_{50} and Z-score were determined for each compound's three biological replicates using the cell viability % values and GraphPad Prism 5. The mean IC_{50} of the biological replicates with standard deviation (SD) served as the final IC_{50} of each compound.

Cytotoxicity

African green monkey kidney epithelial (Vero) cells (Cellonex, South Africa) were cultured and the basal cytotoxicity of the synthesized compounds with antileishmanial and/or anticancer activity was evaluated using the resazurin assay, as described in Mangwegape *et al.* [3] Emetine served as the standard cytotoxic drug. Further use of reference drugs, controls, data analysis, calculations and IC_{50} determinations were identical to that of the antileishmanial assay.

The IC_{50} and Z-score were determined for each qualifying compound's biological replicate using GraphPad Prism 5. For the final IC_{50} of each compound, the mean IC_{50} of the biological replicates were calculated with standard deviation (SD).

References

1. A. Kulshrestha, V. Bhandari, R. Mukhopadhyay, V. Ramesh, S. Sundar, L. Maes, J. C. Dujardin, S. Roy and P. Salotra, *Parasitology research*, 2013, **112**, 825-828.
2. J. L. Siqueira-Neto, O. Song, H. Oh, J. Sohn, G. Yang, J. Nam, J. Jang, J. Cechetto, C. B. Lee, S. Moon, A. Genovesio, E. Chatelain, T. Christophe and L. H. Freitas-Junior, *PLoS Negl Trop Dis*, 2010, **4**, e675-e675.
3. D. K. Mangwegape, N. H. Zuma, J. Aucamp and D. D. N'Da, *Archives des Pharmazie*, 2021, **354**, e2000280.
